# Long non-coding RNAs display higher natural expression variation than protein-coding genes in healthy humans

**DOI:** 10.1186/s13059-016-0873-8

**Published:** 2016-01-29

**Authors:** Aleksandra E. Kornienko, Christoph P. Dotter, Philipp M. Guenzl, Heinz Gisslinger, Bettina Gisslinger, Ciara Cleary, Robert Kralovics, Florian M. Pauler, Denise P. Barlow

**Affiliations:** CeMM Research Center for Molecular Medicine of the Austrian Academy of Sciences, Lazarettgasse 14, AKH BT 25.3, 1090 Vienna, Austria; Present Address: Institute of Science and Technology Austria, Lab Building East, Am Campus 1, A-3400 Klosterneuburg, Austria; Department of Internal Medicine I, Division of Hematology and Blood Coagulation, Medical University of Vienna, Vienna, Austria; Present Address: Piso 23, Av. Santa Fe No 481, Lomas de Santa Fe, 05349, D.F. Mexico

**Keywords:** lncRNAs, expression variation, lncRNA identification, human genome annotation, granulocytes, transcriptome, natural variation, lncRNA features

## Abstract

**Background:**

Long non-coding RNAs (lncRNAs) are increasingly implicated as gene regulators and may ultimately be more numerous than protein-coding genes in the human genome. Despite large numbers of reported lncRNAs, reference annotations are likely incomplete due to their lower and tighter tissue-specific expression compared to mRNAs. An unexplored factor potentially confounding lncRNA identification is inter-individual expression variability. Here, we characterize lncRNA natural expression variability in human primary granulocytes.

**Results:**

We annotate granulocyte lncRNAs and mRNAs in RNA-seq data from 10 healthy individuals, identifying multiple lncRNAs absent from reference annotations, and use this to investigate three known features (higher tissue-specificity, lower expression, and reduced splicing efficiency) of lncRNAs relative to mRNAs. Expression variability was examined in seven individuals sampled three times at 1- or more than 1-month intervals. We show that lncRNAs display significantly more inter-individual expression variability compared to mRNAs. We confirm this finding in two independent human datasets by analyzing multiple tissues from the GTEx project and lymphoblastoid cell lines from the GEUVADIS project. Using the latter dataset we also show that including more human donors into the transcriptome annotation pipeline allows identification of an increasing number of lncRNAs, but minimally affects mRNA gene number.

**Conclusions:**

A comprehensive annotation of lncRNAs is known to require an approach that is sensitive to low and tight tissue-specific expression. Here we show that increased inter-individual expression variability is an additional general lncRNA feature to consider when creating a comprehensive annotation of human lncRNAs or proposing their use as prognostic or disease markers.

**Electronic supplementary material:**

The online version of this article (doi:10.1186/s13059-016-0873-8) contains supplementary material, which is available to authorized users.

## Background

Long non-protein coding RNAs (lncRNAs) have emerged as a fundamental new layer of genomic information in diverse species [1]. They are considered to participate primarily in mRNA gene regulation [2–5] and to play roles in development and disease [6–8]. LncRNAs may be medically relevant as prognostic factors, disease markers, and drug targets [9–13]. To date, it is known that lncRNA genes are abundant in the genomes of human ([14], http://www.gencodegenes.org/stats.html), mouse ([15, 16], http://www.gencodegenes.org/mouse_stats.html), other vertebrates [17–20], plants [21], and simple model organisms such as *C. elegans* [22] and yeast [23, 24]. Although large numbers of lncRNAs have been identified, they have not yet been completely annotated in any organism. Human lncRNAs annotated by the GENCODE project comprise the largest public dataset containing 15,877 lncRNA genes (version 21: http://www.gencodegenes.org/stats/archive.html#a21). Many human annotation projects use cell lines [25], however, some also use primary tissues [14, 26]. An incomplete annotation may arise from two known features of lncRNAs - low abundance and tight tissue-specificity [14, 25]. Notably, lncRNA annotations differ not just between tissues, but also between closely related cell types [27, 28]. Thus, a comprehensive map of all lncRNA genes in the human genome would require systematic and deep analysis of all human body cell types. A recent attempt to define the human lncRNA landscape used several thousand normal and malignant samples and identified almost 47,000 new lncRNA genes [29], supporting earlier predictions that lncRNAs may outnumber protein-coding genes in human [30].

Relatively small numbers of mammalian lncRNAs have been assigned a function. A new functional lncRNA database lists only 181 human transcripts (http://www.lncrnadb.org/, [31]). While it is possible that some lncRNA transcription is a consequence of the local chromatin state [32–34], the gap between annotation and proven functionality reflects the considerable challenges in the analysis of non-coding compared to coding transcripts [35–39]. A deeper knowledge of lncRNAs as a transcript class has followed from genome-wide characterizations of their biology and genomic features with mRNAs as a reference point (reviewed in [30, 34, 40]). Both types of transcripts are transcribed by RNAPII, possess histone modifications typical of active or inactive genes and can be spliced, capped, and polyadenylated (reviewed in [41]). However, in addition to the basic lack of an open reading frame and functional translation [42], some studies have identified characteristics that differentiate lncRNAs from mRNAs. In comparison to mRNAs, lncRNAs are generally found to be more lowly-expressed, show higher tissue-specificity and be enriched in the nucleus [14, 25]. Many lncRNAs initiate from enhancer-like promoters that lack H3K4me3 histone modifications typical of standard mRNA promoters [28, 43], or from repetitive transposable elements normally absent from standard mRNA promoters [44]. In terms of genome and biology features, lncRNAs are usually shorter with fewer exons and show inefficient co-transcriptional splicing [45] and reduced stability [46]. They also show low sequence conservation and evolve faster than mRNAs [47–49].

One lncRNA feature not yet fully investigated in comparison to mRNAs that may influence identification and functional characterization is their natural expression variation. Protein-coding and lncRNA expression and transcript structure have been shown to be dependent on genetic variation in the human lymphoblastoid cell line (LCL) collection [50–52]. Analysis of protein-coding gene expression in whole human blood shows expression variation attributable to inter-individual (for example, age, BMI) and lifestyle (fasting status, smoking) differences, and technical issues such as sampling time, collection and preparation [53, 54]. In this study we use human primary granulocytes, a relatively pure cell type routinely obtained in clinics from healthy individuals and potentially useful diagnostically, to assess natural variability of lncRNA expression. We first prepared an RNA-seq dataset from 10 healthy individuals to define a human granulocyte transcriptome, not previously available. From this we annotated 6,249 lncRNA transcripts arising from 1,323 previously reported and 268 novel lncRNA loci. We show that examining granulocytes from multiple donors allows the identification of less well expressed, less efficiently spliced, and more granulocyte-specific lncRNAs. We then estimated lncRNA expression reproducibility and variability in granulocyte RNA-seq data from seven healthy individuals sampled in three replicates with approximately 1-month intervals. This inter- and intra-individual comparison demonstrated that although lncRNA expression is reproducible between replicates from the same individual, it is significantly more variable between individuals compared to mRNAs. Analysis of multiple tissues from the GTEx project [55] and lymphoblastoid cell lines from the GEUVADIS project [50] supports this conclusion and also shows that higher natural expression variability compared to protein-coding genes is a general feature of lncRNAs. Using the latter dataset we show that natural expression variability markedly influences lncRNA identification as the number of identified lncRNAs increases with the number of donors analyzed and does not reach saturation even with 120 donors. Together, the data show that high expression variability of lncRNAs is an important general feature, which not only additionally distinguishes them from mRNAs, but also will make it necessary to consider the number of individuals in strategies to comprehensively annotate and assign putative functions to lncRNAs in the human genome.

## Results

### Defining the human granulocyte lncRNA transcriptome

**Fig. 1:**
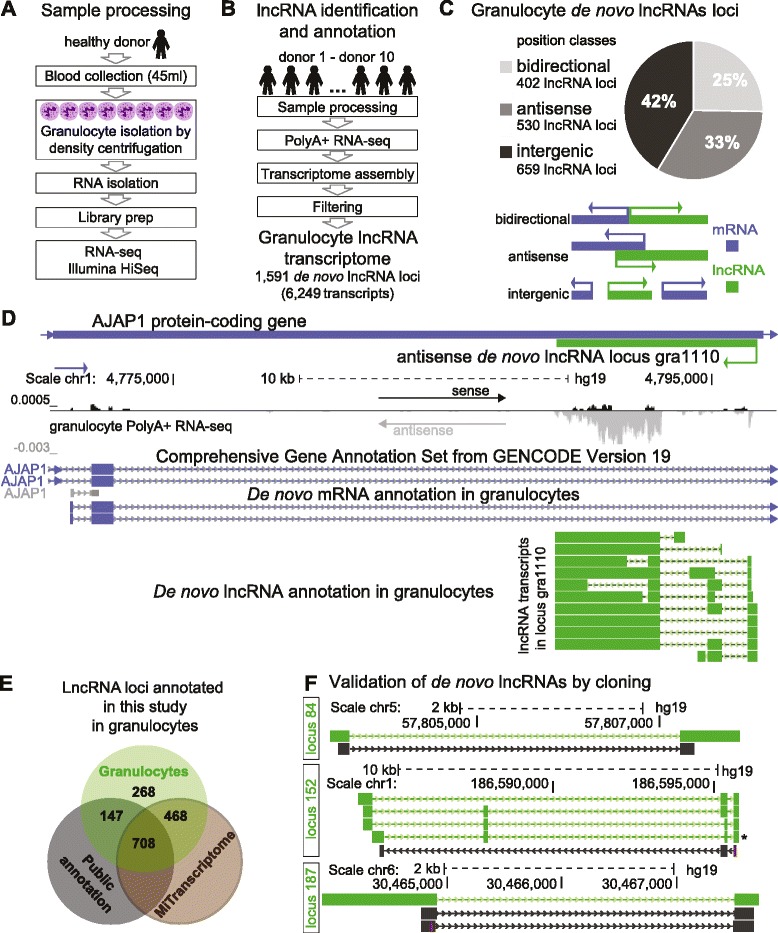
Defining the lncRNA transcriptome of human primary granulocytes. **a** Sample processing overview. **b** LncRNA identification overview. Granulocyte PolyA+ RNA-seq data from 10 donors was used for transcriptome assembly and filtered to create an annotation with 1,591 lncRNA loci containing 6,249 lncRNA transcripts (Additional file 1: Figures S1-3). **c** Positional classification of lncRNA loci relative to the nearest protein-coding gene. Twenty-five percent (402) are bidirectional (light gray), 33 % (530) are antisense (medium gray), and 42 % (659) are intergenic (dark gray). Positional classes are illustrated underneath (blue: protein-coding gene, green: lncRNA). **d** Example of a novel granulocyte antisense lncRNA locus. Top: 3' part of *AJAP1* protein-coding gene (blue) and the novel antisense *gra1110* lncRNA locus (green). Underneath: normalized to read number RNA-seq signal from sample D2-2_pa_100ss (Additional file 2B); GENCODE-v19 protein-coding genes (blue lines) and *de novo* annotated mRNAs (blue) and lncRNAs (green) showing lncRNA transcripts in locus *gra1110* (Additional files 3, 4, and 6). **e** Overlap of granulocyte *de novo* lncRNA annotations (green) with commonly used public lncRNA annotations (gray) (RefSeq: 8,236 lncRNA transcripts, GENCODE-v19: 23,898 lncRNA transcripts, Cabili [14]: 21,630 lncRNA transcripts) and the ‘MiTranscriptome’ annotation (brown) [29]. **f** Validation of granulocyte *de novo* lncRNAs by cloning. Three *de novo* lncRNA loci (84, 152, 187) are shown (see also Additional file 1: Figures S4-S8). Top to bottom for each: scale and chromosome, *de novo* lncRNA transcript annotation in each locus (green isoforms), cloning result (black lines) showing BLAT alignment of the Sanger sequenced cloned cDNA

To annotate lncRNAs in human granulocytes we collected samples from five male and five female healthy individuals of varying ages under standardized sampling conditions and sequenced polyadenylated (PolyA+) RNA (Fig. 1a, b, Additional file 1: Figure S1A and Supplemental Methods, Additional file 2A). Ribosome-depleted RNA-seq, used for expression and splicing efficiency analysis, was performed for seven donors (4 male donors, 3 female donors) at three time points. To annotate lncRNAs we aligned the PolyA+ RNA-seq data with STAR [56] to obtain 757 million uniquely-mapped reads of which 187.6 million were spliced (Additional file 2B, C) and performed *de novo* transcriptome assembly using Cufflinks and Cuffmerge [57]. The term ‘*de novo*’ is used for transcripts/loci identified in this transcriptome assembly pipeline. Only multi-exonic transcripts longer than 200 base pairs (bp) were retained and several filtering steps applied to remove potential assembly artifacts (Additional file 1: Figure S1). We next extracted multi-exonic transcripts overlapping exons annotated as protein-coding in GENCODE-v19 [58] and RefSeq [59] and used them later to generate a *de novo* protein-coding granulocyte mRNA annotation. We discarded annotated GENCODE-v19 pseudogene transcripts. To remove potential protein/peptide-coding transcripts, we estimated transcript protein-coding capability using RNAcode [60] and CPC [61]. We adjusted the criteria for the output of the protein-coding potential estimation pipeline (RNAcode score <18, CPC score <1.6) by analyzing well-known lncRNAs (Additional file 2D). We validated these criteria by applying the pipeline to the above public annotations; this identified the majority of annotated lncRNAs as non-protein-coding, whereas the majority of mRNAs were identified as protein-coding (Additional file 1:Figure S1E). To avoid confusion in later expression analysis we removed all lncRNAs overlapping a protein-coding gene in sense direction (for example, intronic lncRNAs) from our analysis. The final *de novo* lncRNA granulocyte annotation comprised 1,591 lncRNA loci (Additional file 3) expressing 6,249 lncRNA transcripts (Additional file 4) with a mean of 3.9 transcript isoforms per locus, consistent with previous observations [14]. *De novo* lncRNA transcripts contained 13,058 unique exons from 5,612 non-overlapping exonic regions. Protein-coding mRNAs were *de novo* annotated in preference to using the public annotations to avoid technical bias when comparing lncRNAs to mRNAs and to assess the quality of our annotation (Additional file 1: Figure S2). The *de novo* granulocyte mRNA annotation comprised 10,092 mRNA loci (Additional file 5) expressing 132,864 transcripts (Additional file 6) with a mean of 13.2 transcripts per locus, consistent with previous observations [62]. We assigned *de novo* annotated lncRNAs into three position-based classes relative to the nearest protein-coding gene (Fig. 1c). The majority of lncRNA loci (42 % comprising 659 loci) are intergenic, while 33 % (530 loci) are antisense and 25 % (402) are bidirectional. Figure 1d shows an example of a *de novo* annotated antisense lncRNA locus (green lines) absent from public databases.

### Identification of new lncRNA loci and isoforms

We compared our granulocyte *de novo* lncRNA annotation to the most commonly used public annotations: GENCODE-v19 (23,898 lncRNA transcripts) [58], RefSeq (8,236 lncRNA transcripts) [59], and Cabili *et al.* (21,630 lncRNA transcripts) [14] and found that 46 % (736) of granulocyte *de novo* lncRNA loci were not present in public annotations, while 54 % (855) had a full or partial sense overlap with a publicly annotated lncRNA. Exon comparison with the three public annotations showed that we identified 5,694 new unique exons from 2,986 non-overlapping exonic regions. This shows that granulocytes have a specific lncRNA landscape that needs to be defined prior to granulocyte transcriptome analysis. To further assess the novelty of the annotated granulocyte *de novo* lncRNA loci we examined the MiTranscriptome lncRNA annotation based on 7,256 RNA-seq libraries from different human tissues, tumors, and cell lines [29]. Together, this shows that while 83 % of the lncRNA loci identified in this study can be found in one of the four above lncRNA annotations, 268 (17 %) are not found (Fig. 1e). To test the reliability of our granulocyte *de novo* lncRNA annotation we first determined that over 80 % of transcripts were supported by at least one exonic overlap with a spliced EST (human ESTs, UCSC table browser) (Additional file 1: Figure S3A). Second, the MiTranscriptome lncRNA annotation [29] provided an additional validation as 78 % of our granulocyte *de novo* annotated lncRNAs were supported by an exonic overlap with a spliced MiTranscriptome lncRNA (Additional file 1: Figure S3B) with a median of 51 % exonic coverage of granulocyte *de novo* lncRNAs by MiTranscriptome lncRNAs (Additional file 1: Figure S3C). Public lncRNAs annotations had less overlap with our annotation (Additional file 1: Figure S3B) and showed poorer exonic coverage (Additional file 1: Figure S3C) and thus provided support for fewer of our granulocyte *de novo* lncRNA transcripts. In contrast, *de novo* mRNAs were well covered by public mRNA annotations and MiTranscriptome (Additional file 1: Figure S3B, D), indicating that the poor lncRNA coverage may arise from incomplete annotation of this transcript type in public annotations. Last, we used exon-spanning RT-PCR to test granulocyte *de novo* annotated lncRNA splice junctions (Additional file 2E). We confirmed 42 out of 46 tested junctions from 22 granulocyte lncRNA loci. We also cloned lncRNA transcripts from 18 granulocyte *de novo* lncRNA loci not present in public annotations, to confirm their full-length exon structure, continuity, and chromosome position (Additional file 1: Figures 1F, S4-S8 and Additional file 2F). Cloned sequences were deposited in GENBANK (Additional file 2G). In summary, we created a reliable lncRNA transcriptome annotation in healthy human granulocytes that identifies 1,591 lncRNA loci of which 17 % had not previously been described. Furthermore, we demonstrate that granulocyte *de novo* lncRNAs in contrast to mRNAs are incompletely represented in public annotations.

### Non-mRNA-like features that may confound lncRNA annotation

**Fig. 2:**
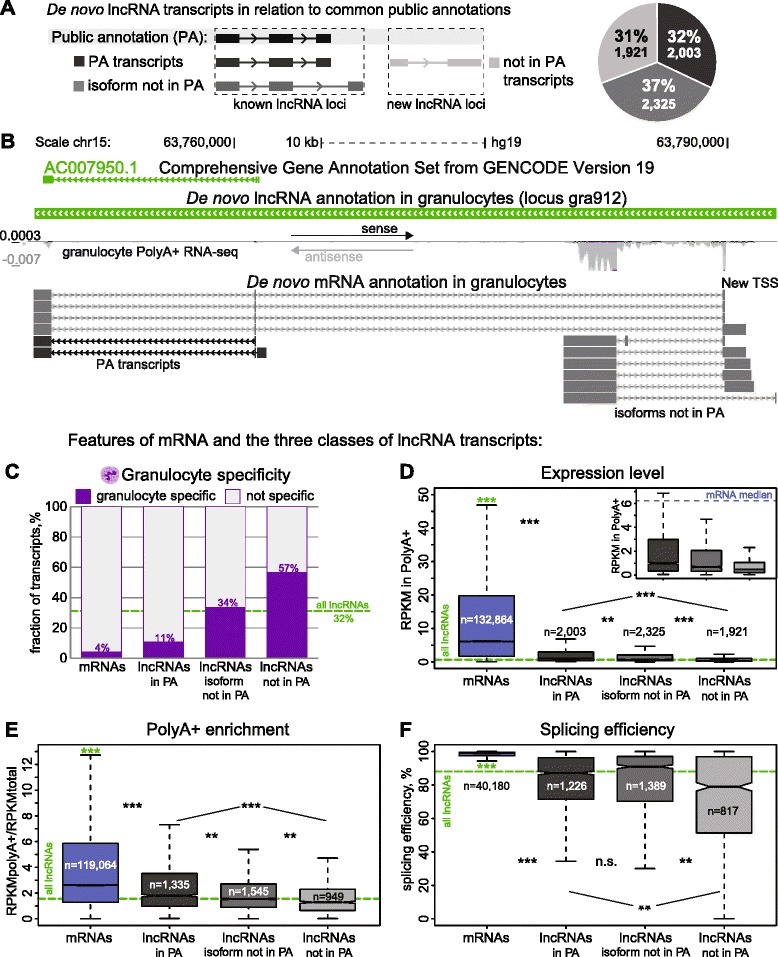
LncRNAs not in public annotations show less mRNA-like features. **a** Distribution of 6,249 granulocyte *de novo* annotated lncRNA transcripts according to coverage by three commonly used public annotations (PA): RefSeq, GENCODE-v19, Cabili [14, 58, 59]. Known lncRNA loci contain two transcript types: ‘PA transcripts’ that show full exonic overlap with an annotated lncRNA transcript (32 %, 2,003 transcripts, dark gray), or ‘isoform not in PA’ transcripts, that can share exons but contain one or more additional exons not present in public annotation (37 %, 2,331 transcripts medium gray). New lncRNA loci: contain 1,921 ‘not in PA’ transcripts (31 % of lncRNA transcripts identified in granulocytes, light gray). **b** An example of a publicly-annotated lncRNA locus (GENCODE-v19 AC007950.1) that contains additional upstream exons not in PA, from sample D2-2_pa_100ss (Additional file 2B). The annotation identifies locus *gra912* (thick green bar). The annotated lncRNA isoforms of locus *gra912* with alternative transcription start sites (TSS) are shown underneath as gray lines (the shorter PA transcript is shown in black for comparison). **c** Granulocyte-specificity analysis. Bar plot shows the percentage of granulocyte-specific (purple) and not-specific (light gray) transcripts *de novo* annotated in granulocytes. Each bar shows the percentage of granulocyte-specific transcripts for each transcript class while the dashed green line shows the percentage for all lncRNAs together. **d** Average expression level (RPKM) in granulocyte PolyA+ RNA-seq samples used for annotation. The median values are: all mRNA transcripts (blue): 6.14, all lncRNA transcripts (green dashed line): 0.65, lncRNA transcripts ‘in PA’ (dark gray): 1.00, lncRNA transcripts ‘isoform not in PA’ (medium gray): 0.68, lncRNA transcripts ‘not in PA’ (light gray): 0.47. **e** PolyA+ enrichment of *de novo* granulocyte annotated transcripts calculated as a ratio between abundance of a transcript in PolyA+ RNA and abundance in total ribosome-depleted RNA. Transcript abundance (RPKM) is averaged among all PolyA+ RNA-seq samples or all total RNA-Ribosomal depleted RNA-seq samples. Transcripts not detected in total RNA-seq data (average RPKM <0.2) were not analyzed. The median values are: all mRNA transcripts (blue): 2.62, all lncRNA transcripts (dashed green line): 1.56, lncRNA transcripts ‘in PA’ (dark gray): 1.80, lncRNA transcripts ‘isoform not in PA’ (medium gray): 1.54, lncRNA transcripts ‘not in PA’ (light gray): 1.29. **f** Splicing efficiency of *de novo* granulocyte annotated transcripts. Only transcripts with average RPKM >0.2 in 21 ribosomal-depleted RNA-seq samples were analyzed and the efficiency of the most efficiently-spliced site in each transcript is plotted. The median values are: all mRNA transcripts: 99.02 %, all lncRNA transcripts: 88.13 %, lncRNA transcripts ‘in PA’: 87.18 %, lncRNA transcripts ‘isoform not in PA’: 90.90 %, lncRNA transcripts ‘not in PA’: 77.97 %. Remarks to boxplots **d**, **e**, and **f**: the box plot displays the full population but *P* values are calculated using Mann–Whitney U test on equalized population sizes. *0.001 < *P* < 10^-5^, **10^-5^ < *P* < 10^-10^, ***P < 10^-16^. Green asterisks indicate the significance of the difference between mRNAs and all lncRNAs (only the median level is plotted as a dashed green line). Outliers are not displayed

As a basis to investigate why our granulocyte *de novo* annotation identified novel lncRNAs we classified them (Fig. 2a) according to existing public annotations (PA) as new lncRNA loci formed by ‘not in PA’ transcripts, or, as ‘known lncRNA loci’ formed by transcripts sharing all exons (PA transcripts) or sharing at least one exon (isoform not in PA, see example in Fig. 2b representing a novel isoform inside a publicly annotated lncRNA locus). The distribution was uniform with 32 % (2,003) ‘PA transcripts’, 37 % (2,235) ‘isoform not in PA’ and 31 % (1,921) ‘not in PA transcripts’. We examined these three lncRNA classes for four known lncRNA features (tissue-specific expression, low expression level, PolyA+ enrichment, and splicing efficiency), which could reduce their identification in RNA-seq data compared to mRNAs.

To examine tissue-specificity we used publicly available RNA-seq data from 34 human cell types (ENCODE project (https://www.encodeproject.org), Illumina Human Body Map Project (http://www.ebi.ac.uk/gxa/experiments/E-MTAB-513) (Additional file 2H). These data were aligned as in Fig. 1b and expression levels calculated for *de novo* annotated granulocyte transcripts. A transcript was considered granulocyte-specific if its expression in granulocytes was at least three-fold higher than in all other cell types. We found granulocyte-specific expression of 32.5 % (1,927) *de novo* annotated lncRNA transcripts and 4 % of *de novo* annotated mRNA transcripts (Fig. 2c, Additional file 1: Figure S9A). This trend was also observed for granulocyte-specific expression over the whole locus, indicating it is not an artifact of the greater number of mRNA isoforms in the *de novo* annotation (Additional file 1: Figures S9B and S10). The same analysis performed for GENCODE-v19 transcripts that are annotated from multiple sample types shows a decreased percentage of lncRNAs (9.0 %) and mRNAs (1.5 %) identified as granulocyte-specific, but a similarly large difference (six-fold) between the two transcript types (Additional file 1: Figure S9C). Analysis of tissue-specific expression performed separately for the three lncRNA transcript classes described above, shows that ‘in PA’ lncRNAs were more similar to GENCODE-v19 transcripts being depleted for granulocyte-specific transcripts compared to the bulk population (dashed green line, Fig. 2c), while ‘not in PA’ and ‘isoform not in PA’ transcripts showed equal or increased granulocyte-specificity.

Expression level is another feature strongly differentiating lncRNAs and mRNAs. We calculated RPKMs of granulocyte *de novo* lncRNA and mRNA transcripts in the PolyA+ data used for the *de novo* annotation, which showed that lncRNA transcripts are 10-fold less abundant than mRNAs (0.65/6.14, respectively; Fig. 2d). We noted that lncRNA/mRNA expression difference was slightly reduced (seven-fold median difference) when analyzing ribosomal-depleted datasets, indicating lncRNA under-representation in PolyA+ RNA (Additional file 1: Figure S11A). Comparing the three lncRNA transcript classes showed that ‘in PA’ transcripts display highest expression and ‘not in PA’ have lowest expression among the three classes in both PolyA+ (see inset, Fig. 2d) and ribosomal-depleted (Additional file 1: Figure S11F) data.

The third feature that may influence lncRNA identification is their reduced polyadenylation efficiency, as this would lower abundance in the PolyA+ fraction usually used for transcript identification. Given our above observation of poorer lncRNA representation in PolyA+ versus ribosome-depleted datasets, we compared transcript abundance in these granulocyte datasets to estimate the enrichment of lncRNAs and mRNAs in the PolyA+ fraction (Fig. 2e). While mRNAs showed a median 2.6-fold enrichment, lncRNAs showed a significantly lower median 1.6-fold enrichment (dashed green line, Fig. 2e). We tested if this difference was influenced by low lncRNA expression levels by splitting transcripts into expression bins (Additional file 1: Figure S12A). This showed that independently of absolute expression levels, lncRNAs show significantly lower PolyA+ enrichment compared to mRNAs. Comparing the three lncRNA transcript classes demonstrated that ‘not in PA’ and ‘isoform not in PA’ transcripts showed significantly lower PolyA+ enrichment than ‘in PA’ transcripts (Fig. 2e).

Inefficient splicing is a fourth feature likely to reduce full-length lncRNA transcripts in the PolyA+ fraction. We used granulocyte ribosomal-depleted RNA-seq to calculate the splicing efficiency of every splice site in lncRNA and mRNA transcripts and defined transcript splicing efficiency as that of its most efficiently processed splice site (Additional file 1: Figure S13A, B). This shows that splicing is significantly less efficient for lncRNAs compared to mRNAs with a median splicing efficiency of 88.13 % (dashed green line, Fig. 2f) and 99.02 %, respectively. This splicing efficiency difference is independent of expression level and also persists at the locus level, that is, independently of the transcripts number per locus (Additional file 1: Figures S12B and S13C). The inefficient splicing of lncRNAs is supported by the experimental validation of lncRNA spliced products described above, which identified abundant unspliced isoforms together with spliced isoforms (see examples in Additional file 1: Figures S5B, S5C, S7A, and S13B, E). Comparing the three lncRNA transcript classes showed that ‘not in PA’ transcripts have lower splicing efficiency than the bulk population analysis (Fig. 2f). The similar splicing efficiency in classes ‘isoform not in PA’ and ‘in PA’ arises from transcripts sharing some splice sites. The reduced splicing of lncRNAs ‘not in PA’ was confirmed by analysis on the locus level (Additional file 1: Figure S13D).

**Fig. 3:**
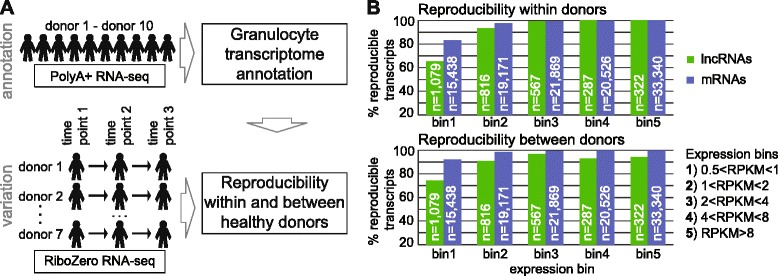
Reproducibility of *de novo* lncRNA and mRNA expression. **a** Study overview. Top: the granulocyte *de novo* transcriptome annotation was generated from 10 healthy donors. Bottom: seven donors were sampled at three time points spaced by ≥1 month (Additional file 2A) and RNA was sequenced to assess intra-individual (using three time points from one donor) and inter-individual (using samples from seven different donors) expression reproducibility. **b** Granulocyte intra-individual (top) and inter-individual (bottom) expression reproducibility for *de novo* annotated lncRNAs (green) and mRNAs (blue). Transcripts detectable (RPKM >0.2) at each of three time points or not detected (RPKM <0.2) at any time point in all seven donors show intra-individual reproducibility. Transcripts detectable in each of seven donors (average RPKM of three replicates >0.2) show inter-individual reproducibility. Five expression bins were used: (1) 0.5 < RPKM ≤1; (2) 1 < RPKM ≤2; (3) 2 < RPKM ≤4; (4) 4 < RPKM ≤8; and (5) RPKM >8 (n = transcript number per bin). Chromosomes X, Y were discarded

In addition to these four RNA biology features, we examined four genomic features. This showed that compared to mRNAs, lncRNAs transcripts have significantly fewer exons, their transcription starts are less CG-rich but more repeat-rich, and their exons contain more repeats (Additional file 1: Figures S11B-E and S12C). With the exception of the median exon number, these features were more extreme in ‘not in PA’ and ‘isoform not in PA’ lncRNAs than in the class of ‘in PA’ lncRNAs. Together this shows that new granulocyte lncRNAs identified in this study have less mRNA-like features that further distinguish them from mRNAs compared to the bulk lncRNA population. To support this claim we performed the same analysis for MiTranscriptome mRNAs and lncRNAs [29], which also shows that lncRNAs not in public annotations have less mRNA-like features (Additional file 1: Figures S14 and S15). Thus we show that features such as tight tissue-specificity and low expression, reduced enrichment in PolyA+ selected RNA and reduced splicing efficiency, not only distinguish lncRNAs from mRNAs, but by reducing their representation in the analyzed transcriptome make their identification more challenging.

### LncRNAs are reproducibly expressed within one donor but vary between donors

We next investigated reproducibility of lncRNA expression in healthy individuals to assess if this could also influence the lncRNA discovery. To estimate expression reproducibility within or between donors, we examined expression in granulocytes from seven donors sampled at three time points spaced by at least 1 month (Fig. 3a, Additional file 2A). These 21 samples were subject to ribosome-depleted RNA-seq (Additional file 2B) aligned with STAR and expression levels were determined of all *de novo* annotated lncRNAs and mRNAs. We first tested if annotated transcripts were reproducibly expressed within one donor, that is, the three time points for each donor should show consistent lncRNA expression (RPKM >0.2) or absence (RPKM ≤0.2) of expression (Fig. 3b top). This analysis was performed separately for transcripts with different expression levels. Expression levels for each donor were calculated by averaging RPKMs from the three time points and a transcript was placed into a bin according to its maximal expression level among the seven donors. We counted the number of reproducibly-expressed transcripts and found that lncRNAs are less reproducible in bins 1 and 2, but above RPKM >2 almost all *de novo* annotated lncRNAs and mRNAs (99–100 %) were reproducibly detected within one donor. In contrast, comparing expression between the seven donors showed consistent lower expression reproducibility of lncRNAs compared to mRNAs (Fig. 3b, bottom). In the three highest expression bins, mRNAs showed 100 % reproducibility while lncRNA transcripts only reached 95 %. In summary, this qualitative analysis shows that, above an expression threshold (RPKM >2), lncRNAs are as reproducibly expressed as mRNAs within replicates from one donor. However, lncRNAs show less reproducible expression than mRNAs between different donors.

### LncRNAs show high expression variability between donors

**Fig. 4:**
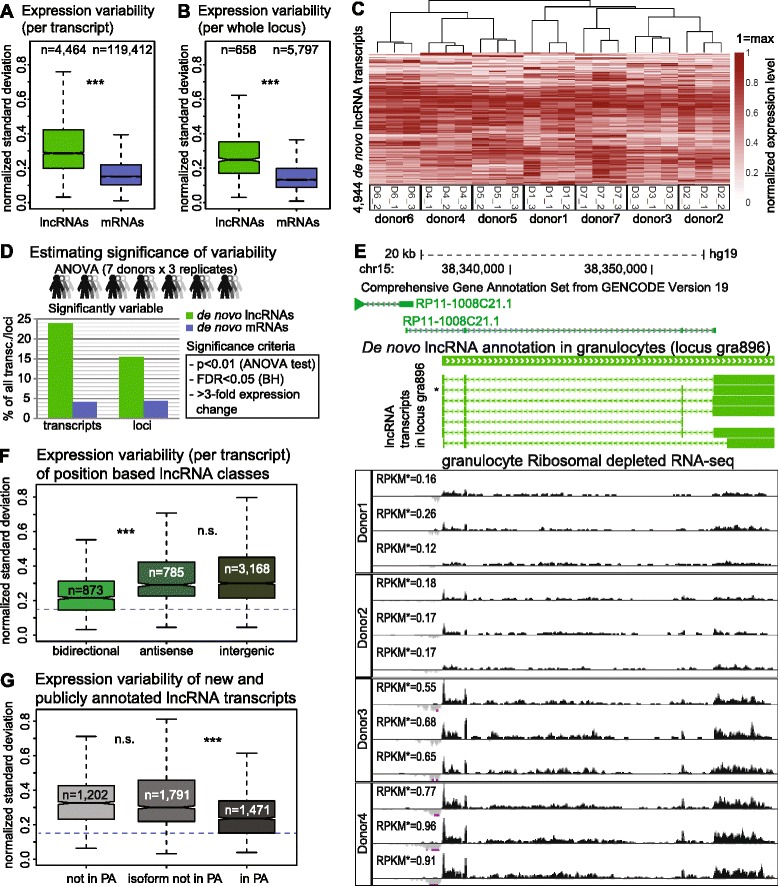
LncRNAs are more variably expressed than mRNAs. **a**, **b** Genome wide inter-individual variability (normalized standard deviation between expression of each transcript/locus in granulocytes from seven donors) of *de novo* granulocyte lncRNA (green) and mRNA (blue) transcripts (**a**) and loci (**b**). Donor expression level is averaged from three replicates (***P <10^–16^). Median values: lncRNA transcripts: 0.29, mRNA transcripts: 0.15, lncRNA loci: 0.26, mRNA loci: 0.15. **c** LncRNA inter-individual expression variability allows correct clustering (normalized level among seven donors) of three time points per donor. Maximum transcript expression among all 21 samples is set to 1 (red), minimum is 0 (white). Clustering was performed using *pheatmap* function in R (clustering_distance_rows = ‘euclidean’, clustering_distance_cols = ‘correlation’). Only transcripts detected (RPKM >0.2) in at least one of the total RNA-seq samples were analyzed. Chromosomes X, Y were discarded. **d** Significance of granulocyte *de novo* lncRNA and mRNA expression variability in seven donors assessed by ANOVA test (the three time points are used as replicates). Bars show the percentage of significantly variable lncRNA (green) and mRNA (blue) transcripts (left) and loci (right). Criteria for calling a transcript/locus ‘significantly variable’: ANOVA test *P* value <0.01, FDR (Benjamini-Hochberg correction) <0.05, fold change between highest and lowest expression in seven donors >3. Only transcripts/loci with RPKM >0.2 in at least one donor are included. Chromosomes X and Y were discarded from the analysis. Total number analyzed: lncRNA transcripts 4,464, mRNA transcripts 119,412, lncRNA loci 658, mRNA loci 5,797. **e** Example of a significantly variable transcript from lncRNA locus *gra896.* Top: an alternative *gra896* TSS overlaps the publicly-annotated lncRNA RP11-1008C21.1 locus. Underneath: normalized total RNA-seq signal for three replicates of four donors scaling from -0.001 (reverse strand, light gray) to 0.004 (forward strand, black). Calculated expression level of the annotated lncRNA transcript marked with * is shown for each RNA-seq track. Significance result for this transcript among seven donors: ANOVA test *P* = 10^–7^, FDR (Benjamini-Hochberg) = 10^–6^, expression fold change = 5.2). **f** Bidirectional lncRNA transcripts show reduced expression variability. Boxplots show inter-individual variability of lncRNA transcripts split according to their position relative to protein-coding genes as in Fig. 1c. Median normalized standard deviation values: bidirectional: 0.22, antisense: 0.29, intergenic: 0.30. Dashed blue line indicates median expression variability of all *de novo* mRNA transcripts. **g** Inter-individual expression variability is lower for known ‘in PA’ lncRNA transcripts compared to those newly annotated in granulocytes (‘not in PA’ and ‘isoform not in PA’). Median normalized standard deviation values: ‘not in PA’: 0.33, ‘isoform not in PA’: 0.30, ‘in PA’: 0.24. Dashed blue line indicates median expression variability of all *de novo* mRNA transcripts. Remarks to boxplots **a**, **b**, **c**, **g**: Transcripts/loci not expressed (RPKM <0.2) in any of seven donors (total RNA-seq data) and data from chromosomes X, Y were discarded and outliers are not displayed. The box plot displays the full population but *P* value is calculated using Mann–Whitney U test on equalized sample size. n.s. not significant, ***P <10^–16^

We quantitated the extent of expression variability between the seven donors by calculating the standard deviation of granulocyte *de novo* lncRNA and mRNA expression (Fig. 4a, b). As RPKM is a parametric value and ranges from 0.2 (the used expression cutoff) to several thousand, we normalized standard deviation of expression for each gene between donors by the mean of expression among the seven donors (thus calculating the value also known as the coefficient of variation). We performed this analysis calculating variability of expression for each transcript separately (Fig. 4a), and expression of the whole locus disregarding identified exon structures (Fig. 4b, Additional file 1: Figure S10). Both analyses showed that lncRNAs display significantly higher variability than mRNAs (*P* <10^–16^). LncRNA and mRNA expression variability between donors (inter-individual) was significantly higher than between the replicates from one donor (intra-individual). In addition, both inter- and intra-individual expression variability of lncRNAs exceeded that of mRNAs (Additional file 1: Figure S16). The high inter-individual variability of lncRNA expression allowed unsupervised clustering of the three time point samples according to each of the seven donors (Fig. 4c), that validates their use as replicates.

LncRNA expression is generally lower than that of mRNAs (Fig. 2d, Additional file 1: Figure S11A), which could bias the expression variability analysis, as lower expression will correlate with higher normalized standard deviation values. We controlled for this by distributing transcripts and loci into expression bins (Additional file 1: Figure S17). This showed that while variability anti-correlates with expression level for both lncRNAs and mRNAs, lncRNAs analyzed at the transcript or loci level show consistently more expression variability than mRNAs, independent of absolute expression level. We additionally plotted expression variability against mean expression between all donors for lncRNA and mRNA transcripts and loci (Additional file 1: Figure S18A, B). This showed that the trend lines of the anti-correlation between variability and expression level are clearly distinct for lncRNAs and mRNAs at both transcript and loci level, with lncRNAs displaying higher variability. Thus, high natural expression variability is not an artifact of the general low expression of lncRNAs. To identify the number of lncRNA and mRNA transcripts and loci significantly variable between donors we applied an ANOVA test (*aov* function in R [63]) to expression values in all the 21 (that is, seven donors sampled three times) ribosomal depleted RNA-seq samples. We find that 23.9 % (1,069) of lncRNA transcripts but only 4.2 % of mRNA transcripts are differentially expressed between the seven donors (transcripts RPKM >0.2, Fig. 4d). This trend persisted when applying an ANOVA test to expression over whole loci (Fig. 4d, 15.5 % and 4.4 % for lncRNA and mRNA loci, respectively). Importantly, this difference between lncRNAs and mRNAs was persistent when analyzing different expression bins (Additional file 1: Figure S19A). Figure 4e shows an example of a significantly variable lncRNA expressed from chromosome 15. Among the four displayed tracks donors 3 and 4 show higher expression, consistent among three replicates, while donors 1 and 2 show low expression consistent among replicates. Since 25 % of *de novo* annotated lncRNAs are bidirectional and likely share a promoter with an mRNA (Fig. 1c), we examined if this class resemble mRNAs in having less expression variability. Figure 4f shows that bidirectional lncRNA transcripts more closely resembled mRNAs and were significantly less variable than antisense or intergenic lncRNAs and this trend was also observed in all expression bins and over the whole locus (Additional file 1: Figure S20A-C).

### Publicly annotated lncRNAs show less expression variability

To further confirm high lncRNA expression variability and to investigate its impact on lncRNA identification, we analyzed expression variability of publicly annotated (Additional file 1: Figure S21A, B) and of MiTranscriptome (Additional file 1: Figure S22A, B) lncRNAs and mRNAs in our granulocyte RNA-seq data. All annotations confirmed high lncRNA expression variability compared to mRNAs. However, the extent of the lncRNA/mRNA difference was reduced when analyzing public annotations compared to the MiTranscriptome annotation and our *de novo* granulocyte annotation, which both identified numerous novel lncRNAs. We then estimated expression variability separately for the three lncRNA classes described in Fig. 2a, and found that transcript types ‘not in PA’ and ‘isoform not in PA’ showed significantly higher variability between the seven donors, compared to ‘in PA’ transcripts (Fig. 4g) and this trend was observed in all expression bins (Additional file 1: Figure S23A) and also when analyzing expression over whole locus for ‘new’ and ‘known’ lncRNA loci (Additional file 1: Figure S23B, C). To test this further, we analyzed expression variability of MiTranscriptome lncRNAs classified according to their presence in public annotations (as described in Additional file 1: Figure S14D). This showed that ‘not in PA’ and ‘isoform not in PA’ MiTranscriptome lncRNAs displayed higher expression variability (Additional file 1: Figure S22C), consistent with results for the *de novo* granulocyte lncRNA annotation. Together this supports our arguments above, that lncRNAs not in public annotations have less mRNA-like features.

### A list of robustly or variably expressed lncRNAs in human primary granulocytes

Following the discovery of high intra- and inter-individual expression variability of lncRNAs we sought to generate a list of robustly expressed and variably expressed granulocyte lncRNAs as a resource. To generate the robustly expressed list we filtered 6,249 lncRNA transcripts in our annotation (that is, the set of transcripts that ‘can be’ expressed in granulocytes) to identify those detected (RPKM >0.2) in all replicate samples from seven donors. This gave a robustly expressed annotation of 2,490 transcripts from 393 lncRNA loci (Additional file 7A). We applied stricter criteria and required a higher level of expression (RPKM >1) in every sample to produce another annotation of ‘well-expressed robust’ lncRNAs in granulocytes with 817 transcripts from 115 lncRNA loci (Additional file 7B). A list of significantly variably expressed (defined as in Fig. 4d) lncRNAs with 1,069 transcripts from 214 lncRNA loci is provided in Additional file 8.

### LncRNAs expression variability in lymphoblastoid cell lines (LCL)

**Fig. 5:**
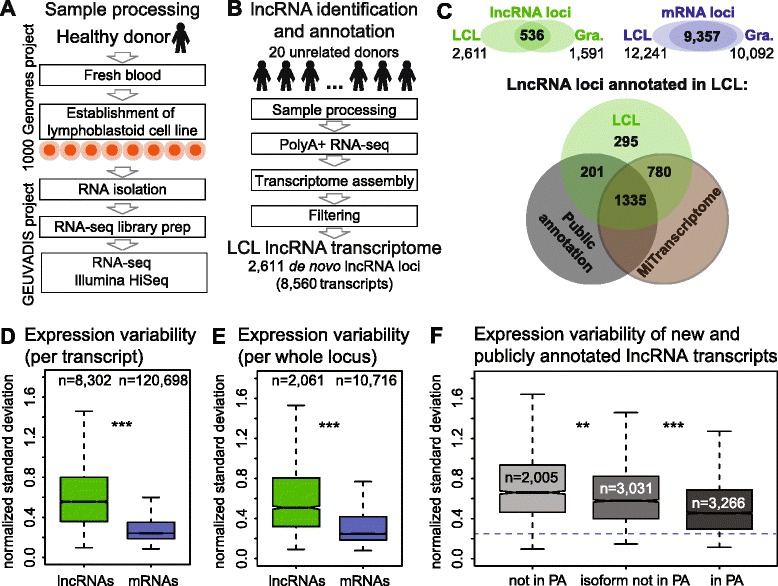
GEUVADIS RNA-seq data confirm increased lncRNA expression variability. **a** Sample processing overview: 462 lymphoblastoid cell lines (LCL) established from healthy donors by EBV transformation were processed by the GEUVADIS RNA-seq Project [50]. **b** LncRNA identification overview. We picked 20 unrelated donors (total of 522 million uniquely mapped reads) from 462 donors and processed the raw RNA-seq data through the same pipeline used to annotate lncRNAs in granulocytes (Additional file 1: Figure S24). The resulting LCL lncRNA transcriptome contained 2,611 lncRNA loci formed by 8,560 lncRNA transcripts. **c** Top: overlap between LCL and granulocyte *de novo* transcriptome annotations created in the study. A total of 536 of 2,611 LCL lncRNA loci overlap granulocyte loci. A total of 9,357 of 12,241 LCL *de novo* mRNA loci overlap granulocyte loci. Bottom: overlap of *de novo* lncRNA annotation in LCL with commonly used public annotations (PA): RefSeq, GENCODE-v19, and Cabili [14, 58, 59], and the MiTranscriptome annotation [29] identifies 295 new lncRNA loci. Of these, only 18 loci overlap the *de novo* lncRNA granulocyte annotation. **d, e** LncRNAs show higher expression variability than mRNAs in LCL. The boxplots show inter-individual variability of LCL lncRNA (green) and mRNA (blue) transcripts (**d**) and loci (**e**). Inter-individual variability is estimated by calculating standard deviation between expression of each transcript/locus in 462 donors normalized to the mean expression. Both transcripts and loci variability is significantly (***P <10^–16^) different between lncRNAs and mRNA. Median values: lncRNA transcripts: 0.56, mRNA transcripts: 0.24, lncRNA loci: 0.51, mRNA loci: 0.25. **f** Inter-individual expression variability is higher for newly annotated lncRNA transcripts in LCL. Boxplot shows inter-individual expression variability of LCL lncRNA transcripts split according to coverage by public annotations (PA), which is higher for ‘not in PA’ and ‘isoform not in PA’ lncRNA transcripts compared to ‘in PA’. Median normalized standard deviation values: not in PA: 0.66, isoform not in PA: 0.58, in PA: 0.46. Blue dashed line indicates median expression variability of all *de novo* mRNA transcripts in (**d**). Remarks to boxplots **d**, **e**, **f**: transcripts or loci not expressed (RPKM <0.2) in any of the 462 donors were discarded. The box plot displays the full population but *P* value is calculated using Mann–Whitney U test on equalized sample size (**P <10^–10^, ***P <10^–16^). Data from chromosomes X, Y were discarded and outliers are not displayed

To test our finding of high lncRNA expression variability in an independent cell type and with larger donor numbers, we analyzed GEUVADIS project data (http://www.geuvadis.org/web/geuvadis/rnaseq-project [50]) consisting of PolyA+ non-stranded RNA-seq of lymphoblastoid cell lines (LCL) sampled once from 462 healthy individuals of various ages from five population groups (Fig. 5a) (ENA http://www.ebi.ac.uk/ena/data/view/ERR188021-ERR188482). Since LCL are a different cell type to granulocytes, we created a *de novo* LCL annotation via our pipeline (Additional file 1: Figure S24A). From the list of 462 samples, we used RNA-seq data from 20 unrelated donors (2 female donors and 2 male donors from each population with a total of 522 (26.1 million reads/donor) million uniquely mapped reads and 177.8 million spliced reads) grouped into five pools (Additional file 2I). The resulting LCL lncRNA transcriptome consisted of 2,611 lncRNA loci (Additional file 9) formed by 8,560 lncRNA transcripts (Additional file 10) with a mean of 3.3 transcripts per locus (Fig. 5b). The lncRNA transcripts contained 17,009 unique exons from 9,379 non-overlapping regions. We also annotated 12,241 *de novo* mRNA loci formed by 124,799 transcripts, with a mean of 10.1 transcript per locus. The overlap of LCL and granulocyte *de novo* lncRNA transcriptomes comprised only 536 loci (21 %) whereas the *de novo* mRNA transcriptomes overlapped by 9,357 loci (76 %), which is consistent with lncRNA high tissue-specificity (Fig. 5c). The increase in lncRNA loci number from 1,591 in granulocytes, to 2,611 in LCL may reflect increased transcriptional activity of LCL compared to primary granulocytes or the two-fold increase in donor number used for annotation (see data below). Comparison of the LCL *de novo* lncRNA annotation to public annotations and MiTranscriptome showed that 2,316 (89 %) of LCL lncRNA loci are covered by the four lncRNA annotations while 295 (11 %) are not found (Fig. 5c). The LCL annotation quality was verified in a similar manner as for the granulocyte annotation (Additional file 1: Figure S24B-G). LncRNA classification by coverage from public annotations shows that 1,536 are known loci containing 3,363 (39 %) ‘in PA’ while 3,111 (36 %) are ‘isoform not in PA’ transcripts, and 1,075 are new loci formed by 2,086 (25 %) ‘not in PA’ transcripts (Additional file 1: Figure S25). Exon comparison showed that *de novo* lncRNA annotation in LCL contained 6,113 unique exons not present in public annotations from 4,150 non-overlapping exonic regions. Similar to granulocytes, LCL lncRNA transcripts not in public annotations show less mRNA-like features (Additional file 1: Figure S26).

We used the LCL *de novo* annotation to calculate the RPKM of every transcript and locus in all 462 donors. An ANOVA test could not be applied due to the absence of donor replicates, but inter-individual variability was calculated from the normalized standard deviation of expression between all donors. Comparing lncRNAs to mRNAs showed that lncRNAs are significantly more variable both when calculating expression of transcripts or over whole loci (Fig. 5d, e). We controlled for expression level by distributing transcripts or loci to expression bins as described above and found that except for bin1 transcripts, lncRNAs were significantly more variable in expression than mRNAs (Additional file 1: Figure S27). To complete the comparison with the granulocyte data, we found LCL bidirectional lncRNAs to be significantly less variable than intergenic lncRNAs in all expression bins (Additional file 1: Figure S28). In addition, LCL *de novo* lncRNAs not covered by public annotations (‘not in PA’ transcripts) show significantly more expression variation than publicly annotated transcripts (Fig. 5f, Additional file 1: Figure S29). This analysis of an independent cell type with an independent sample collection and processing method from a larger number of donors supports our finding of high inter-individual lncRNA expression variability.

### LncRNA expression variability is increased in multiple human tissues

**Fig. 6:**
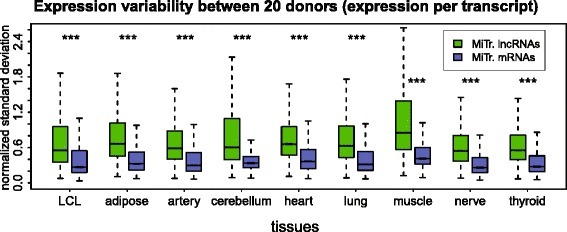
GTEx RNA-seq data show increased lncRNA expression variability in multiple human tissues. Inter-individual variability of multi-exonic MiTranscriptome lncRNA (green) and mRNA (blue) transcripts analyzed in GTEx RNA-seq dataset [64]. Twenty donors per tissue are analyzed (Additional file 2J). Standard deviation is normalized to the mean expression among all 20 analyzed donors for each tissue. Only transcripts expressed in the given tissue in at least one donor (RPKM >0.2) are displayed. Number of transcripts in each box from left to right: LCL (lncRNAs: 28,571; mRNAs: 102,449), adipose (lncRNAs: 38,060; mRNAs: 113,688), artery (lncRNAs: 29,965; mRNAs: 108,082), cerebellum (lncRNAs: 44,912; mRNAs: 115,039), heart (lncRNAs: 32,827; mRNAs: 111,564), lung (lncRNAs: 39,909; mRNAs: 117,901), muscle (lncRNAs: 31,507; mRNAs: 106,099), nerve (lncRNAs: 39,167; mRNAs: 115,038), and thyroid (lncRNAs: 40,099; mRNAs: 116,206). Median expression values from left to right: LCL: 0.55, 0.27, adipose: 0.66, 0.32, artery: 0.59, 0.30, cerebellum: 0.60, 0.33, heart: 0.66, 0.36, lung: 0.63, 0.31, muscle: 0.85, 0.41, nerve: 0.54, 0.26, and thyroid: 0.56, 0.27. The box plots display the full population but *P* values are calculated using Mann–Whitney U test on equalized sample size (***P <10^–16^). Data from chromosomes X, Y were discarded and outliers are not displayed

The above analysis shows high lncRNA expression variability relative to mRNAs in a primary human cell type (granulocytes) as well as in cell lines immortalized from lymphocytes. To test if this is a general phenomenon in human tissues, we obtained access to the GTEx project RNA-seq data [55, 64]. We downloaded RNA-seq data for nine human tissues: LCL, adipose, artery, cerebellum, heart, lung, muscle, nerve, and thyroid from 20 individuals per tissue (Additional file 2J). We used the MiTranscriptome transcript annotation derived from multiple tissue types [29], to calculate lncRNA and mRNA expression in GTEx samples and then estimated expression variability as described above using 20 donors per tissue (Fig. 6). This shows that lncRNAs are significantly more variable than mRNAs in all the analyzed tissues. We performed a binned expression control as described above and found that, apart from bin 1 that showed inconsistent results in two tissues, all nine tissues showed a significant increase of lncRNA expression variability independent of expression level (Additional file 1: Figure S30). Together with the above data on granulocytes and LCLs, this demonstration of increased lncRNA expression variability relative to mRNAs in multiple human tissues indicates that it is a general phenomenon inherent to all human tissues and a new lncRNA feature.

### Increased expression variability affects lncRNA identification

**Fig. 7:**
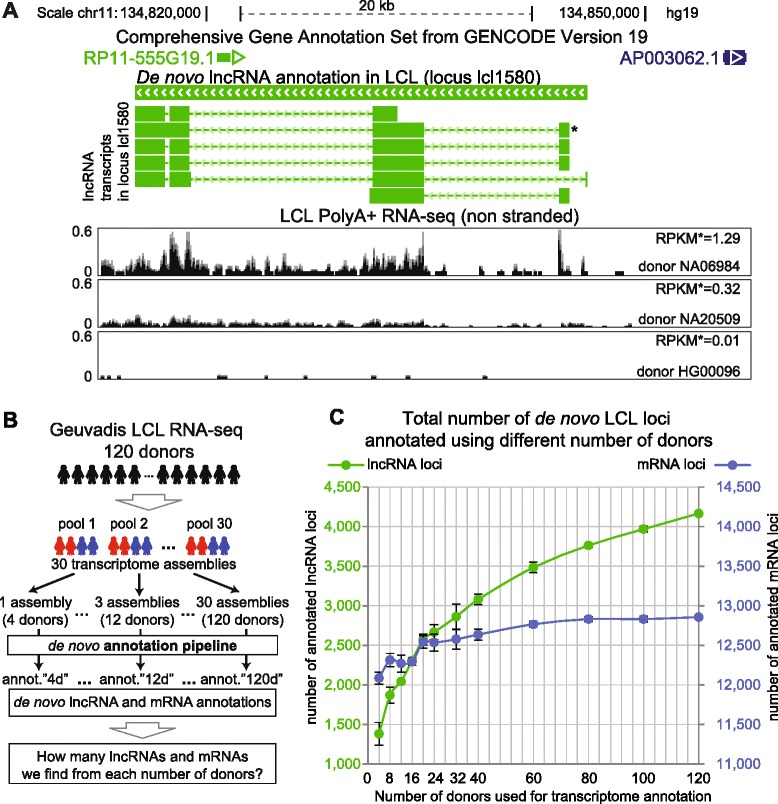
Increasing donor number identifies more lncRNA loci. **a** Example of a highly variable LCL lncRNA locus lcl1580 not in public annotations. GENCODE-v19 annotates lncRNA RP11-555G19.1 and protein coding gene AP003062.1 transcribed in antisense direction to lcl1580 (top). Normalized non-strand-specific PolyA+ RNA-seq signal for three donors is displayed (scaling from 0 to 0.6). RPKM of the *transcript isoform is shown for each sample. **b** Analysis overview. GEUVADIS project LCL RNA-seq data from 120 donors was used to create 30 data pools (each with 100 million reads from two female (red) and two male (blue) donors) and to assemble 30 transcriptomes (Methods). An increasing number of assemblies (corresponding to from 4 to up to 120 donors) was merged to serve as input into the *de novo* lncRNA and mRNA identification pipeline (Additional file 1: Figure S1A). This created a series of LCL *de novo* lncRNA and mRNA annotations from an increasing number of donors. **c** LCL *de novo* lncRNA (green) and mRNA (blue) loci number annotated using increased donor number. Left: Y-axis for lncRNA loci (green). Right: Y-axis for mRNA loci (blue). The range of values is set to 3,500 on both Y-axes. Maximum number of lncRNA / mRNA loci annotated (at 120 donors): 4,166 / 12,857. Error bars: standard deviation of loci number between three replicates of random picking for each number of assemblies used (Additional file 11C)

We demonstrated above the high lncRNA inter-individual expression variability in diverse human tissues (Figs. 4a, b, 5d, e and 6) as well as the increased expression variability of novel compared to known lncRNAs (Figs. 4g and 5f, Additional file 1: Figure S22C). We asked if this expression variability feature could influence lncRNA identification. Figure 7a shows an example of a highly variably expressed *de novo* annotated LCL lncRNA not covered by public annotations (but identified with different exon models in [29]) that is well expressed (RPKM >1) in one out of 462 donors in the GEUVADIS project dataset, expressed at a low level (RPKM >0.2) in 93 donors and not detected (RPKM <0.2) in the remaining 368 donors. It is likely that such a lncRNA has a low chance of discovery when analyzing few individuals. We hypothesized that adding more individuals to the identification pipeline may increase the chance of identifying highly variably expressed lncRNAs. At the same time, given the relatively low inter-individual expression variability of mRNAs, we would expect to identify a relatively constant number of mRNA loci.

We tested this by *de novo* annotating lncRNAs and mRNAs from a variable number of individuals. We picked 120 GEUVADIS LCL donors (Fig. 7b, Additional file 11A), unified the data by sampling 25 million paired-end reads from each donor and created 30 pools, each with four donors (two male and two female donors) with a total of 100 (25 × 4) million reads. From the 30 pools we created 30 LCL *de novo* transcriptome assemblies using Cufflinks. We randomly picked 1, 2, 3, 4, 5, 6, 8, 10, 15, 20, 25 (using three replicates of random picking), and 30 assemblies, which corresponded to 4, 8, 12, 16, 20, 24, 32, 40, 60, 80, 100, and 120 donors, respectively, and applied Cuffmerge and the *de novo* transcriptome annotation pipeline to each group of assemblies (Additional file 1: Figure S31A, B and Additional file 11B). Only one pool (100 million reads) was fed at a time into the assembly pipeline, thus the sensitivity of Cufflinks was unchanged. In addition, assemblies but not reads were merged at this stage. Different number of assemblies fed into our annotation pipeline produced multiple lncRNA/mRNA annotations with different numbers of loci and transcripts. We plotted the number of mRNA and lncRNA loci (averaged from the three replicates described above) versus the number of donors used (Fig. 7c, Additional file 11C). This showed that while lncRNA loci number (green lines) grew three-fold with increasing donor number, from 1,382 loci obtained from four donors to 4,166 obtained from 120 donors, the number of mRNA loci (blue lines) shows a much lower level of increase from 12,085 (four donors) to 12,857 loci (120 donors). This supports the hypothesis that adding more individuals to the identification pipeline increases the number of lncRNA loci but not the number of mRNA loci.

In contrast to the loci analysis, the number of transcript isoforms increased with similar kinetics for both lncRNAs and mRNAs (approximately seven-fold increase from four to 120 donors; Additional file 1: Figure S31C). The difference between lncRNAs and mRNAs is that an increasing donor number allows identification of an increasing number of transcript isoforms inside a stable number of mRNA loci, while lncRNAs retain a low median number of transcripts per locus and increase the number of loci annotated in the genome (Additional file 1: Figure S31D). Note that we did not expect to find non-annotated mRNAs loci since the mRNA *de novo* identification pipeline was limited to annotated mRNA genes. If the analysis did identify non-annotated mRNA loci they would be recognized among lncRNA candidates that were filtered by the pipeline step that estimated transcript protein-coding capability. However, this step only removed a low-level increase from 83 (four donors) to 198 (120 donors) loci (Additional file 1: Figure S31E). The slight increase in mRNA loci number with increasing donor number (Fig. 7c) likely arises from high inter-individual expression variability of a small number of mRNAs in LCLs. The larger increase in lncRNA loci number also arises from identifying more highly variable annotated lncRNAs when analyzing more donors, but also potentially by identifying novel lncRNA loci.

Assembling transcriptomes from pools of 100 million paired-end read does not increase Cufflinks sensitivity (Additional file 1: Figure S31A), but including more donors into the identification pipeline naturally increased the number of transcriptome assemblies merged and therefore the total amount of the RNA-seq data analyzed (from 1 to 30 × 10^8^ sequencing reads). To control that this strategy did not only lead to the identification of marginally-expressed lncRNAs we plotted the RPKM of lncRNAs added to annotation with the addition of more donors (Additional file 1: Figure S32). This shows that median level of expression (in at least one donor used for identification) of newly-identified lncRNAs is RPKM of approximately 1, which means that 50 % of the newly-identified lncRNA transcripts are well-expressed (RPKM >1). This median level also does not decrease for transcripts that are only found with large donor numbers. In addition, we analyzed the dynamics of lncRNA identification with increasing the donor number in different expression bins (Additional file 1: Figure S33). This shows that lncRNAs from high-expression bins contribute substantially to the overall increase in lncRNA loci and transcript number. For example, four donor annotations identified 314 lncRNA transcripts initiating from 152 different loci in bin4 (that is, at least one donor used for identification expresses the transcript with 4 < RPKM < 8), while annotating from 120 donors identified 3,518 bin4-lncRNA transcripts initiating from 610 loci. Thus, while marginally-expressed lncRNAs are identified by adding more donors to the analysis, they only constitute a fraction of the newly-identified transcripts. Both controls show that identification of an increasing number of lncRNAs cannot be solely attributed to stochastic sampling sensitivity and identification of lowly-expressed transcripts, but likely arises from genuine expression variability between individuals.

We next asked if the lncRNA loci identified with increased donor numbers were new or known loci (as defined in Fig. 2a) and what were the dynamics of their identification. To do this we plotted the normalized number (the number of loci at 120 donors set to 100 %) of known (dark gray) and new (light gray) lncRNA loci versus donor number (Figure S34 in Additional file 1: Figure S34 and Additional file 11C). For comparison the same plot shows the dynamics of mRNA (dashed blue line) and all lncRNA (dashed green line) identification from the data in Fig. 7b. This shows that although the number of known lncRNA loci increases with donor number from 948.5 (four donors,) to 2,103 (120 donors), the number of novel lncRNA loci shows a more striking increase from 433.7 to 2063 loci (2.2-fold and 4.8-fold, respectively; Additional file 1: Figure S34) (note that non-integer loci numbers arise from averaging three replicates). While mRNA loci identification plateaued with four donors, the known lncRNA loci identification curve starts to plateau with >80 donors, but the new lncRNA identification curve does not plateau up to 120 donors.

Finally, we used the most comprehensive *de novo* annotation from 120 donors as a reference transcriptome to build a ‘donor saturation curve’ to test how well this annotation can be recreated using fewer individuals. We counted the number of reference 120 donor lncRNA and mRNA loci identified (defined by >50 % coverage, Additional file 1: Figure S35A, top, Additional file 11D) using a reduced number of donors. The resulting curve saturates for mRNAs, but does not saturate for lncRNAs even with 120 individuals. Only 27 % of lncRNA loci identified with 120 donors were identified using four donors, this increased to 50 % at 20 donors and thereafter continuing to rise. The difference between known and new lncRNA loci was consistent with observations in Additional file 1: Figure S34. We also assessed how well the exon structure of mRNAs and lncRNAs from the reference 120 donor annotation was recreated by annotations obtained using fewer donors (Additional file 1: Figure S35B). Median exonic coverage of mRNAs was above 90 % just using four donors, whereas lncRNAs require 80 donors to reach similar levels of exonic coverage. In summary, these analyses show that increasing the donor number will identify more lncRNA loci, however, the donor number required is vastly in excess of that required for mRNAs.

## Discussion

An appreciation of the need to define the lncRNA landscape of the whole human genome is increasing with the number of known lncRNAs genes and with an understanding of the unique qualities of their biology. Although the GENCODE annotation comprises the largest public dataset with 15,877 lncRNA genes (version 21: http://www.gencodegenes.org/stats/archive.html#a21), later studies that used several thousand normal and malignant samples from numerous individuals identified four-fold more lncRNA genes [29]. Why the number of lncRNAs continues to rise apparently in excess of protein-coding gene number, is not yet clear. In this study we set out to annotate the lncRNA transcriptome of freshly harvested human granulocytes with the goal of investigating lncRNA inter-individual expression variability and determining how this influences lncRNA identification.

The resulting human granulocyte transcriptome obtained from 10 healthy individuals identified 1,591 lncRNA loci with a mean of 3.9 transcripts per locus. The same granulocytes express approximately six-fold more mRNA loci each with approximately three-fold more transcripts. The reduced activity of lncRNA loci relative to protein-coding loci has been noted [14, 62]. Comparing the granulocyte *de novo* annotation to the most commonly used public annotations (GENCODEv19: 23,898 lncRNA transcripts [58], RefSeq: 8,236 lncRNA transcripts [59], Cabili: 21,630 lncRNA transcripts [14]) that together contain 19,762 non-overlapping lncRNA loci, shows that one-third of granulocyte *de novo* lncRNA transcripts are not present and one-third added a new isoform to public-annotated loci. A comparison with the recent massive MiTranscriptome lncRNA annotation containing 46,331 new lncRNA loci [29], showed that 268 granulocyte lncRNA loci (17 % of the annotated granulocyte lncRNA transcriptome) were not previously reported. With the caveat that different annotation pipelines may influence identification, this shows that human granulocytes have a specific lncRNA landscape that needs to be defined prior to transcriptome analysis, rather than relying on integrative lncRNA landscapes from multiple cell types.

The identification of numerous new human granulocyte lncRNA loci is surprising in view of the extremely large numbers present in public annotations or datasets. Because of this we investigated if specific lncRNA biology features contribute to their under-representation in public databases by assessing if they were more prominent in new loci or isoforms. We first investigated four known features, that is, very tight tissue-specific expression, lower expression level, inefficient enrichment in PolyA+ selected fractions, and inefficient splicing (reviewed in [30, 34, 40]). In each case we demonstrated a significant difference for these features between lncRNAs and mRNAs and, in addition, demonstrated that these features are more prominent in new lncRNA loci and transcript isoforms. For example, reports from different species show that lncRNAs compared to mRNAs have tight tissue-specific expression and also are generally more lowly expressed [14, 15, 17, 18, 25, 65]. We found that while only 4 % of mRNA transcripts display granulocyte-specific expression, 32 % of lncRNA transcripts, and 57 % of novel lncRNA transcripts were granulocyte-specific. Similarly, lncRNA expression levels were 10-fold less abundant than mRNAs, as reported in many species (see above references), however expression of novel ‘not in PA’ lncRNA transcripts was 13-fold less abundant. We could also show that lncRNA enrichment in the PolyA+ fraction relative to total ribosomal-depleted fraction was reduced compared to mRNAs (respective median enrichments of 1.6-fold and 2.6-fold) in agreement with findings that a proportion of lncRNAs are not polyadenylated [66] and that this reduction was 1.6-fold greater for novel ‘not in PA’ lncRNA transcripts. A relatively new feature reported for imprinted *cis*-repressor lncRNAs such as *Airn* and *Ube3a-ats* [67, 68] and for some lncRNAs in human K562 cells [45] that could also affect the abundance of full-length transcripts in PolyA+ RNA fractions, is inefficient splicing. We accessed splicing efficiency of lncRNAs and mRNAs in our granulocyte data and showed that compared to mRNAs, lncRNAs are less efficiently spliced with a broad distribution of splicing efficiency. Median lncRNA splicing efficiency was reduced by 10.9 % compared to mRNAs, however, novel lncRNA transcripts showed 22.9 % reduction. We confirmed the inefficient splicing of lncRNAs and the greater reduction in novel lncRNA using the independent MiTranscriptome annotation [29]. Together this analysis shows not only that lncRNAs share several non-mRNA-like biology features, but also that these features are more prominent in new lncRNA loci and transcript isoforms and thus are likely to reduce lncRNA representation in public annotations.

The last feature examined that could influence the incomplete representation of lncRNAs in public databases is that of natural expression variation. We used the granulocyte annotation with seven donors sampled at three time points separated by at least 1 month, to estimate the natural expression variability of lncRNAs relative to mRNAs. This analysis shows that lncRNA expression is unexpectedly highly variable among a population and, while relatively stable over time within an individual, lncRNA expression variation is significantly larger than that of mRNAs independent of expression level. We find that when considering all the 6,249 *de novo* annotated granulocyte lncRNA transcripts only 40 % (2,490) are robustly expressed, while 17 % (1,069) display significant inter-individual variable expression even within the small sample size of seven donors. Importantly, we show that high natural expression variability is not a consequence of the generally low expression of lncRNAs, as lncRNA transcripts/loci in all expression bins were more variable than mRNAs and also displayed higher percentage of significant inter-individual variable expression assessed by ANOVA test. The high inter-individual variability of lncRNA expression was unique enough to allow unsupervised grouping of replicates sampled over several months according to each of the seven donors. We verified high lncRNA inter-individual expression variability by demonstrating a similar difference for MiTranscriptome annotated transcripts expressed in granulocytes. We also analyzed an independent public RNA-seq lymphoblastoid cell dataset from GEUVADIS [50]. This LCL dataset derived from 462 donors displayed an overall higher median expression variability for both mRNAs and lncRNAs than the granulocyte dataset consisting of seven donors; however, the relative two-fold difference between lncRNAs and mRNAs loci and transcripts was similar. In each of the three above analyses we could show that novel lncRNA transcripts display higher expression variability than known lncRNA transcripts. Lastly, we demonstrated that high lncRNA inter-individual expression variability relative to mRNAs is a general phenomenon in human tissues, by analyzing multiple tissues from the GTEx project [64]. Interestingly, although we analyzed the same number of donors per tissue we found different absolute levels of lncRNA and mRNA expression variability, with skeletal muscle displaying the highest and LCL, nerve, and thyroid displaying the lowest variability level. As an important control, analyzing LCL in the GTEx dataset using the MiTranscriptome annotation showed similar levels of expression variability as that obtained by analyzing the GEUVADIS LCL dataset using our *de novo* LCL annotation. Overall, these expression variability analyses of public datasets, in additional to our granulocyte analysis presented here, confirm our conclusions and support the general nature of increased lncRNA natural expression variability compared to mRNAs.

Comparison of lncRNA and mRNA expression variability was performed as a small part of two previous studies. One LCL study analyzing splicing variability of protein-coding genes found a small number (183) of GENCODE lncRNAs with consistent higher expression variability than mRNAs, even in the absence of replicates [69]. The second study [55] reported a similar relative impact of inter-tissue and inter-individual variability to total variance in gene expression for highly expressed (median RPKM >2.5 among 1,641 analyzed samples comprising 43 body sites from 175 individuals) GENCODE-v12 lncRNAs and mRNAs. This implies, given the known increased inter-tissue variability of lncRNAs, that inter-individual variability of lncRNAs is also greater in its absolute value than that of mRNAs. This study additionally reported enrichment of lncRNAs among genes showing differential expression between individuals of different populations. Thus, the findings from both these studies are consistent with our demonstration here of higher natural expression variation of lncRNAs compared to mRNAs.

High lncRNA inter-individual expression variability highlights another striking biology feature that distinguishes lncRNAs from mRNAs. The finding that expression variability is more prominent in new lncRNA loci and reduced in reference lncRNA annotations also indicates it can influence identification. Thus public annotations based on limited numbers of human donors or derived from single animal or plant inbred strains, may have reduced representation of variably expressed lncRNAs. We demonstrate this with the GEUVADIS LCL RNA-seq data derived from one cell type, by showing that adding more donors to the analysis identifies more lncRNA genes in the human genome. The number of lncRNA loci increased continuously, with novel lncRNA showing a more striking increase than known lncRNAs. The MiTranscriptome study that used a donor number per tissue comparable to our LCL analysis [29] identified three-fold more novel lncRNAs than present in the three commonly used public databases (see above references). Our results also indicate that a granulocyte lncRNA annotation based on 10 donors, is most likely at the lower part of the donor saturation curve for this cell type. Moreover, our finding that the identification of novel lncRNA loci does not plateau even with 120 donors indicates that comprehensive annotation of lncRNAs in the human genome requires as many individuals as possible. The identification of high lncRNA intra- and inter-individual expression variability has implications for identifying lncRNAs and assessing their function and potential medical use. LncRNAs that lack consistent expression in some individuals are unlikely to be necessary for normal cell function, but may be functional in an age, environment, lifestyle, or disease related manner as shown for some protein-coding genes [54, 70]. At the same time, it cannot be assumed that a robustly expressed lncRNA has an important function in the cell type in which it is expressed. For example, the developmentally important *Airn* lncRNA retains robust expression after performing its silencing function [71]. Our results support the view that functional studies require an understanding of basic lncRNA biology in different individuals before they can be interpreted [36, 72].

The basis of increased inter-individual expression variation of lncRNAs relative to mRNAs is unknown. It may be relevant that, together with a lower conservation and faster evolution rate, human lncRNAs are recently evolved loci, harboring more SNPs than protein-coding genes [49, 73]. LncRNAs may also be more susceptible to environmental and lifestyle factors that contribute to mRNA expression variation [54]. Studies of protein-coding genes and lncRNAs in LCLs prepared from different population groups conclude that both expression strength and alternative splicing contribute to expression variability [50, 69, 74, 75]. How this contributes to differences in lncRNA and mRNA expression variability is not known. Bidirectional lncRNAs that likely share a promoter with a neighboring protein-coding gene are regulated similarly to neighboring protein-coding genes [76] and we show that compared to intergenic or antisense lncRNAs, expression variability of bidirectional lncRNAs is more similar but still greater, than that of mRNAs. Inter-individual alternative splicing may contribute as some lncRNA loci display unusually high alternative splicing and variable exon structures [77]. However, this is not supported by our observation that expression variation over the whole locus is similar to that of transcript isoforms. LncRNA genes are considered to be similar to mRNA genes as both are transcribed by RNAPII (reviewed in [30, 34]). However, details of their promoters or enhancers that could explain the five non-mRNA-like features highlighted here (tight tissue-specificity, low expression, inefficient PolyA+ selection, inefficient splicing, and high inter-individual expression variation) have not yet been investigated. Some potential gene regulatory features (chromatin-modification patterns, splicing signals) are similar for lncRNAs and mRNAs [14, 18, 25, 78]. Some publications identified non-mRNA-like features in lncRNAs while others stress mRNA-like features, particularly of intergenic lncRNAs [15, 46, 79–81] (reviewed in [30, 34, 40]). The analysis of healthy granulocytes presented here supports the view that a lncRNA subpopulation shows distinct non-mRNA-like features, which now includes high inter- and intra-individual expression variability. Non-mRNA-like features of lncRNAs may have use in their classification, as it is likely to be relevant for their function [82, 83]. We show here that in healthy granulocytes only 40 % (2,490) of lncRNA transcripts are robustly expressed, while 17 % (1,069) of lncRNA transcripts show significant variable expression. The biological significance of robust or variable expression is not yet clear and both classes of lncRNAs may be useful for some studies. However, explanations of lncRNAs in terms of their evolution and function or proposals of their use as biomarkers or therapeutic targets first require an understanding of the robustness of their expression in healthy tissues.

## Conclusions

We demonstrate here by analysis of human granulocyte RNA-seq data from multiple individuals that lncRNAs show unusually high natural expression variability compared to mRNAs. We use this dataset to generate a list of robustly and variably expressed granulocyte lncRNAs that will be of use in future applications. We also show that higher expression variability of lncRNAs is a general phenomenon inherent to diverse human tissues and cell lines that is of yet, unknown biological significance. High natural expression variability of lncRNAs, in addition to their tight tissue-specificity, low expression, inefficient PolyA+ selection, and inefficient splicing, identifies a set of five non-mRNA-like features that distinguish part of the lncRNA population from mRNAs and, also reduces their representation in reference annotations. We show that high inter-individual expression variability offers one explanation for the incomplete annotation of lncRNAs in many genomes. Our analysis shows that increasing the number of individuals analyzed will identify more lncRNA loci in the human genome, however, the donor number required is vastly in excess of that required for mRNAs. The finding of high expression variability of lncRNAs and its effect on identification provides novel guidelines for lncRNA annotation and additional considerations for design of functional studies and personalized medicine approaches.

## Methods

### Sample collection from healthy donors

Ten volunteers (five men, five women; age range: 27–62 years) without obvious disease were recruited to donate blood. Seven volunteers donated blood three times with gaps of 5 to 21 weeks (Additional file 2A). The remainder donated once only. Donors abstained from eating on the morning of donation; 45 mL of venous blood was collected between 10:00 and 11:00 into VACUETTE® Sodium Citrate Coagulation Tubes and processed immediately. Granulocytes were isolated using density gradient centrifugation and immediately used for RNA preparation either depleted for ribosomal RNA using the RiboZero rRNA removal kit Human/Mouse/Rat (Epicentre) or a polyA enriched using the TruSeq RNA Sample Prep Kit v2 (Illumina) (details in Additional file 1: Supplemental Methods).

### RNA-seq library preparation and read alignment

(a) Non-strand-specific libraries were prepared using the TruSeq RNA Sample Prep Kit v2 (Illumina) following the manufacturer’s protocol. (b) Strand-specific library preparation used same kit with modifications [84]. Equal concentrations of barcoded libraries were pooled for 50 bp or 100 bp paired-end sequencing by Illumina HiSeq 2000 (Biomedical Sequencing Facility http://biomedical-sequencing.at/). After base-calling and sample de-multiplexing, the RNA-seq data were provided as archived .fastq or unmapped .bam files. RNA sequencing reads were aligned using STAR aligner with adjusted default parameters [56] (details in Additional file 1: Supplemental Methods).

### RNA-seq read number

Three stranded samples were sequenced per flow cell lane generating 22 to 79 million 100 bp PE reads per sample. Unstranded PolyA+ RNA-seq samples varied from 24 to 38 million 100 bp PE and 64 to 91 million 50 bp PE reads. In total we obtained 17 PolyA+ RNA-seq datasets and 21 total RNA-seq dataset totaling 2.13 billion reads (Additional file 2B).

### Annotating mRNAs and lncRNAs in primary granulocytes

A total of 784 million PolyA+ RNA-seq reads from 10 donors were used to *de novo* annotate lncRNA and mRNA transcriptomes in granulocytes (see details in Additional file 1: Supplemental Methods). The final *de novo* annotation of human primary granulocytes was 132,864 mRNAs forming 10,092 genomic loci (average 13.2 transcripts per locus) and 6,249 lncRNAs forming 1,591 genomic loci (average 3.9 transcripts per locus). Assembly quality was assessed by inspecting *de novo* annotation of well-known lncRNAs like XIST (Additional file 1: Figure S2A) and by analyzing completeness of assembly of RefSeq (Additional file 1: Figure S2B) and GENCODE-v19 (Additional file 1: Figure S2C) annotated mRNAs.

### Positional classification of lncRNAs

lncRNA loci and transcripts were divided into three classes based on their relative position to protein-coding genes. We combined *de novo* mRNA annotation with public protein-coding gene annotations by GENCODE-v19 and RefSeq to obtain the most comprehensive annotation of protein-coding genes in granulocytes. We then called lncRNA loci/transcripts bidirectional if they shared or overlapped a promoter (defined as TSS +/- 1.5 kb) with a protein-coding gene. LncRNA loci/transcripts overlapping a protein-coding gene in the antisense direction were called ‘antisense’ (sense direction overlaps were removed from the annotation). The third position-based class ‘intergenic’, had no overlap with a protein-coding gene.

### Cloning of full-length lncRNA transcripts

RT-PCR was performed on granulocyte cDNA to amplify full-length lncRNA transcripts prior to cloning. PCR primers (http://biotools.umassmed.edu/bioapps/primer3_www.cgi) spanned the transcript from first to the last exon and the PCR product length limited to 1.5 kb (Additional file 2F). Isolated plasmid DNA was Sanger Sequenced and aligned to the human genome using BLAT. Cloned sequences are displayed as a UCSC screen shot with the *de novo* lncRNA annotation, primers, and BLAT alignment (Additional file 1: Figures S4-S8). Seventy-five cloned sequences were submitted to GENBANK (Additional file 2G).

### Public RNA-seq data mining

We downloaded publicly available raw strand-specific RNA-seq data (fastq files) from various cell types/tissues produced by the ENCODE project and Illumina Human Body Map Project (see list in Additional file 2H), processed it as for other sequencing data in the study (see: RNA-seq read alignment).

### RPKM

This was calculated using RPKM_count.py (RSEQC package). Expression of a transcript is the RPKM of exons of a one transcript, expression over a locus is RPKM of the whole locus including intronic signal.

### Splicing efficiency analysis

We estimated splicing efficiency for each splice site of each multiexonic transcript in our ribosomal-depleted granulocyte RNA-seq from seven donors with three time points pooled at the alignment stage to increase coverage. Splicing efficiency of each splice site was calculated separately in each donor. We calculated RPKM of the exonic and intronic boundaries of the splice site (45 bp each, leaving out 5 bp directly at the splice site to allow for imprecision of splice site identification), calculated the ratio of intronic to exonic signal, and by that estimated how efficiently this splice site was used (Additional file 1: Figure S13A). A splice site was discarded if exonic RPKM was below the cutoff (RPKM = 0.2) in any of the seven donors. We then introduced a value ‘Splicing efficiency’ (S), ranging from 0 for completely unused splice sites (intronic signal equal or higher than exonic signal) to 100 for optimally used spliced splice sites (no intronic signal detected). S = 100*(1-RPKMintronic/RPKMexonic). We replaced all the negative S values (when intronic signal was higher than exonic signal) with 0, defining such cases as full absence of splicing. We averaged the splicing efficiency value calculated from seven donors for each splice site. Splicing efficiency of a transcript was then defined as the maximal splicing efficiency achieved by the most efficiently spliced site of that transcript. Splicing efficiency of a locus was similarly defined by the maximal splicing efficiency among all transcripts (all splice sites) in the locus.

### Assigning *P* value to boxplot comparisons

Every boxplot was plotted using values for all the transcripts/loci analyzed (number of transcripts/loci indicated in the boxplot). The difference in population sizes of compared transcript/loci types was accounted for by performing statistical tests on equalized population sizes. Namely, the larger population was randomly subsampled to match the size of smaller population and Mann–Whitney U test was applied to estimate significance of the difference between the populations with equalized sizes. Subsampling and statistical tests were performed three times for each comparison and the three *P* values obtained were averaged to give the resulting *P* value to be indicated on the boxplot.

### Inter-individual expression variability analysis

Inter-individual expression variability was estimated by calculating standard deviation of expression between analyzed donors then normalizing it to the mean expression of the locus/transcript among all analyzed donors. For granulocytes we assessed variability between seven donors (expression of a locus/transcript in each donor was calculated as a mean of expression of the three time points of this donor). For LCL we assessed variability between 462 donors.

### GEUVADIS project RNA-seq data analysis

We downloaded and aligned using a common pipeline all 462 PolyA+ 75 bp paired end RNA-seq raw sequencing datasets provided by GEUVADIS RNA-seq project (http://www.ebi.ac.uk/ena/data/view/ERR188021-ERR188482). The data contained donors from five populations (http://www.1000genomes.org/category/frequently-asked-questions/population). We picked two female and two male unrelated donors from each population and used RNA-seq from these 20 donors to assemble the LCL *de novo* lncRNA and mRNA transcriptome. We pooled the samples into five groups with a similar number of aligned spliced reads (Additional file 2I) and performed transcriptome assembly following the pipeline described for granulocytes. As the RNA-seq datasets were not strand-specific we used strand-specific PolyA+ RNA-seq of GM12878 from the ENCODE project (Additional file 2H) in the pipeline where needed. Quality assembly (Additional file 1: Figure S24B) was assessed as for granulocytes.

### GTEx RNA-seq data analysis

Aligned (as described in [55]) RNA-seq data from the GTEx project (http://www.gtexportal.org/home/) were downloaded from dbGaP (https://dbgap.ncbi.nlm.nih.gov/) as described in (http://www.gtexportal.org/static/misc/GTEx_Poster_CommunityMeeting_TY.pdf) after we applied and were granted data access. We downloaded RNA-seq data for nine tissues (namely lymphoblastoid cell line (LCL), adipose, artery, cerebellum, heart, lung, muscle, nerve, and thyroid), from 10 male and 10 female individuals each (Additional file 2J). The aligned RNA-seq datasets were unstranded and ranged from 14.8 to 85.4 (average 52.1) million paired-end reads each. We calculated RPKM of MiTranscriptome annotated multi-exonic lncRNAs and mRNAs in all samples and performed variability analysis between 20 individuals per tissue.

### Donor saturation curve

One hundred and twenty out of 462 GEUVADIS RNA-seq samples containing more than 25 million reads were picked for the analysis from 12 unrelated women and men from each of the five population groups. A total of 25 million reads were randomly sampled from each RNA-seq sample using DownsampleSam.jar (Picard tools http://broadinstitute.github.io/picard/command-line-overview.html#DownsampleSam). Donors were grouped into 30 groups each with two women plus two men from the same population and the reads from the four donors were pooled using MergeSamFiles.jar (Picard tools http://broadinstitute.github.io/picard/command-line-overview.html#MergeSamFiles) to produce 30 × 100 million read pools. Cufflinks was used to assemble a transcriptome from each pool (Additional file 1: Supplemental Methods) resulting in 30 transcriptome assemblies. Of these 30 assemblies, 1, 2, 3, 4, 5, 6, 8, 10, 15, 20, 25, or 30 assemblies were used to annotate *de novo* LCL transcriptomes from different number of donors (4, 8, 12, 16, 20, 24, 32, 40, 60, 80, 100, and 120, respectively) and to define the relation between the number of loci (Y axis) and the number of donors/assemblies (X axis). We randomly picked the needed number of assemblies from the list of 30. The random picking was performed three times for each number of assemblies (Additional file 1: Figure S31B), except when all 30 assemblies were used for the last point. The picked assemblies were then merged with Cuffmerge and underwent the previously established *de novo* annotation pipeline (Additional file 1: Supplemental Methods).

## Ethics statement

Peripheral blood samples were collected from healthy volunteers after written informed consent at the Vienna General Hospital (Allgemeines Krankenhaus der Stadt Wien, Klinische Abteilung für Hämatologie und Hämostaseologie). The study was approved by the local Ethics committee of the Medical University of Vienna (‘Ethik Kommission der Medizinischen Universität Wien’) and experimental methods comply with the Helsinki Declaration.

## Availability of data

Raw granulocyte RNA-seq data, RPKM, and variability values for granulocyte *de novo* lncRNAs and mRNAs as well as their BED12 annotation files were deposited in NCBI's Gene Expression Omnibus [85] and are accessible through GEO Series accession number GSE70390 (http://www.ncbi.nlm.nih.gov/geo/query/acc.cgi?acc=GSE70390). LncRNA annotations in granulocytes and LCL created in the study are available to directly download as Additional files in bed12 format. Genbank accession numbers for sequenced lncRNAs are listed in Additional file 2G.

## Additional files

Additional file 1:
**Supplemental Figures (S1-S35) with legends and Supplemental Methods.** (PDF 8255 kb) Additional file 2:
**A** **Human granulocyte samples sequenced in this study.** **B** List of human granulocyte RNA-seq datasets produced in the study. **C** Pools used for human granulocyte transcriptome assembly. **D** Well-known lncRNAs used to adjust RNAcode and CPC pipeline output. **E** Validation of *de novo* granulocyte lncRNA splice junctions by means of exon spanning RT-PCR. **F** Validation of *de novo* granulocyte lncRNA transcripts not supported by public annotations by means of cloning and Sanger Sequencing: overview. **G** Validation of *de novo* granulocyte lncRNA transcripts not supported by public annotations by means of cloning and Sanger Sequencing: sequencing results and Genbank accession numbers. **H** Overview of the publicly available RNA-seq datasets used in the study. **I** Pools used for human LCL transcriptome assembly (GEUVADIS raw RNA-seq data used - [50]). **J** Overview of the GTEx RNA-seq datasets used in the study. (XLSX 127 kb) Additional file 3:
**Granulocyte** ***de novo*** **lncRNA loci annotation (1,561 loci): BED12 formatted file can be directly uploaded into UCSC browser.** Column 5 indicates number of transcripts in the locus. (BED 119 kb) Additional file 4:
**Granulocyte** ***de novo*** **lncRNA transcript annotation (6,249 transcripts): BED12 formatted file can be directly uploaded into UCSC browser.** (BED 655 kb) Additional file 5:
**Granulocyte** ***de novo*** **mRNA loci annotation (10,092 loci): BED12 formatted file can be directly uploaded into UCSC browser.** Column 5 indicates number of transcripts in the locus. (BED 765 kb) Additional file 6:
**Granulocyte** ***de novo*** **mRNA transcript annotation (132,864 transcripts): BED12 formatted file can be directly uploaded into UCSC browser.** (BED 23458 kb) Additional file 7:
**A** **List of robust lncRNA transcripts in granulocytes (2,825 transcripts): columns are formatted as a BED12 file.** **B** List of robust well expressed (RPKM >1) lncRNA transcripts in granulocytes (931 transcripts): columns are formatted as a BED12 file. (XLSX 250 kb) Additional file 8:
**Annotation of granulocyte** ***de novo*** **lncRNA transcripts showing significantly variable expression (1,069 transcripts): BED12 formatted file can be directly uploaded into UCSC browser.** (BED 117 kb) Additional file 9:
**LCL** ***de novo*** **lncRNA loci annotation (2,611 loci): BED12 formatted file can be directly uploaded into UCSC browser.** Column 5 indicates number of transcripts in the locus. (BED 197 kb) Additional file 10:
**LCL** ***de novo*** **lncRNA transcript annotation (8,560 transcripts): BED12 formatted file can be directly uploaded into UCSC browser.** (BED 884 kb) Additional file 11:
**Donor saturation curve samples and pools: overview with list of donors, assemblies, and number of loci identified using different number of donors.** **A** List of 120 donors used in the donor saturation study with corresponding population and pool it was grouped into. **B** List of randomly picked pools for each data point. **C** Number of *de novo* lncRNA and mRNA loci annotated using different number of transcriptome assemblies (donors) – data for plotting Fig. 7b, S32C-E and S34. **D** Number of *de novo* lncRNA and mRNA loci from ‘120 donors’ annotation identified using less transcriptome assemblies (donors) - data for plotting donor saturation curve - Figure S35A. **E** Number of *de novo* lncRNAs from different expression bins identified from increasing number of donors - data for plotting Figure S33. (XLSX 34 kb)
