# Supplementary material for: Long non-coding RNAs display higher natural expression variation than protein-coding genes in healthy humans
Source: Genome Biol. 2016 Jan 29;17:14. doi: 10.1186/s13059-016-0873-8 (PMC4731934; doi:10.1186/s13059-016-0873-8)
Supplement: Additional file 1: — Supplemental Figures (S1-S35) with legends and Supplemental Methods. (PDF 8255 kb) [file 13059_2016_873_MOESM1_ESM.pdf]

ADDITIONAL FILE 1 (Kornienko et al.)

Long non-coding RNAs display higher natural expression variation than protein-coding genes in healthy humans

Aleksandra E. Kornienko*, [1]Christoph P. Dotter, Philipp M. Guenzl, [2]Heinz Gisslinger, [2]Bettina Gisslinger, [3]Ciara Cleary, Robert Kralovics, Florian M. Pauler, Denise P. Barlow*

CeMM Research Center for Molecular Medicine of the Austrian Academy of Sciences, Lazarettgasse 14, AKH BT 25.3, 1090 Vienna, Austria

[1]Present address: Institute of Science and Technology Austria, Lab Building East, Am Campus 1, A-3400 Klosterneuburg, Austria

[2]Department of Hematology, Medical University of Vienna, Vienna, Austria

[3]Present address: Piso 23, Av. Santa Fe No 481, Lomas de Santa Fe, 05349, Mexico D.F.

*Corresponding authors (akornienko@cemm.oeaw.ac.at, dbarlow@cemm.oeaw.ac.at)

TABLE OF CONTENTS

Data supporting Figure 1:

Figure S1. *De novo* lncRNA and mRNA annotation in granulocytes 4

Figure S2. Quality of *de novo* transcriptome annotation in granulocytes 6

Figure S3. Validation of granulocyte *de novo* lncRNAs by overlap with other annotations 7

Figures S4-S8. Validation of granulocyte *de novo* lncRNAs by cloning 8

Data supporting Figure 2:

Figure S9. Granulocyte specificity of granulocyte *de novo* and GENCODE annotated lncRNAs and mRNAs 14

Figure S10. Illustration of expression calculation per transcript and over whole locus............ 15

Figure S11. Features that distinguish granulocyte lncRNAs from mRNAs also distinguish novel granulocyte lncRNAs from publicly annotated lncRNAs 16

Figure S12. Difference between mRNAs and lncRNAs is persistent independently of expression level 18

Figure S13. Splicing efficiency calculation 20

Figure S14. Analyzing features of MiTranscriptome lncRNAs and mRNAs confirms that difficult to identify lncRNAs are more different from mRNAs than publicly annotated lncRNAs 22

Figure S15. Additional features that distinguish MiTranscriptome lncRNAs from mRNAs, and newly identified from publicly annotated lncRNAs.. 24

Data supporting Figure 4:

Figure S16. Intra-individual variability is significantly lower than inter-individual variability for both lncRNAs and mRNAs in granulocytes 26

Figure S17. *De novo* lncRNAs are more variable than mRNAs independently of generally lower lncRNA expression level 27

Figure S18. Plotting variability against mean RPKM level confirms increased lncRNA expression variability compared to mRNAs independent of expression level........................... 28

Figure S19. Percentage of *de novo* granulocyte lncRNA transcripts/loci significantly variable among seven donors is higher than that of mRNAs in all expression bins 29

Figure S20. Bidirectional lncRNAs show reduced variability: controls 30

Figure S21. Increased expression variability is observed for RefSeq and GENCODE lncRNAs, however to a lesser extent 31

Figure S22. MiTranscriptome analysis confirms increased lncRNA expression variation....... 32

Figure S23. Granulocyte lncRNA transcripts not present public annotations (PA) show increased variability: controls 33

Data supporting Figure 5:

Figure S24. Defining the lncRNA transcriptome of LCL (Lymphoblastoid cell line).............. 34

Figure S25. Identification of novel lncRNA transcripts and novel isoforms of known lncRNA loci in LCL cells 36

Figure S26. Analyzing features of LCL lncRNAs confirms that difficult to identify lncRNAs are more different from mRNAs than publicly annotated lncRNAs 38

Figure S27. Higher LCL *de novo* lncRNA expression variability is not caused by lower lncRNA expression level. 40

Figure S28. Bidirectional lncRNAs annotated in LCL show reduced variability 41

Figure S29. New lncRNAs are more variable than known lncRNAs from LCL de novo annotation: controls 42

Data supporting Figure 6:

Figure S30. Confirmation of increased lncRNA expression variability in multiple human tissues using GTEx project data: expression level control 43

Data supporting Figure 7:

Figure S31. *De novo* identification of lncRNA and mRNA loci in LCL using variable number of donors. 44

Figure S32. Increasing donor number does not tend to identify only marginally expressed lncRNAs 46

Figure S33. Increasing donor number identifies increased numbers lncRNAs in all expression bins. 47

Figure S34. Number of new lncRNA increases more dramatically with donor number increase compared to known lncRNAs. 48

Figure S35. Donor saturation curve analysis of 120-donor lncRNA and mRNA identification using less donor in the identification pipeline 49

SUPPLEMENTAL METHODS 50

1. Granulocyte isolation 50
2. RNA isolation using TRI reagent 50
3. Reverse transcription 51
4. Ribosomal RNA depletion 51
5. Polyadenylated RNA enrichment 51
6. Preparation of strand-specific RNA-seq libraries 51
7. RNA-seq read alignment 53
8. Public gene annotations used in the study 53
9. Calculation of GC content 54
10. RT- and qRT-PCR primer design 54
11. Annotating mRNAs and lncRNAs in primary granulocytes 54
12. Calculating exonic coverage 58
13. Creating granulocyte specificity estimation heat maps 58
14. RT-PCR to test splicing efficiency calculation 58

SUPPLEMENTAL REFERENCES 60

SUPPLEMENTAL FIGURES & LEGENDS (S1 – S35)

Figure S1

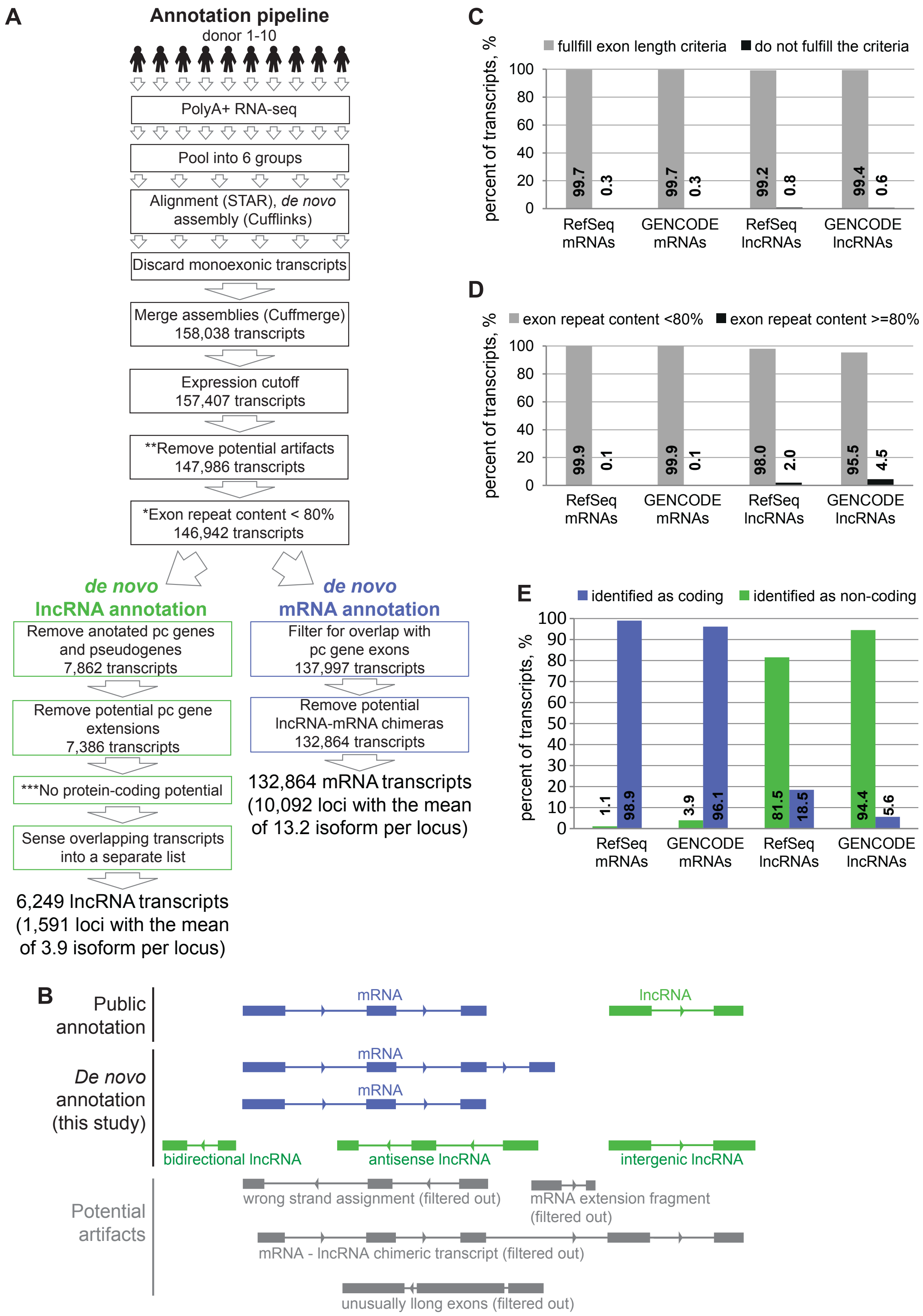

Figure S1. *De novo* lncRNA and mRNA annotation in granulocytes

A. Algorithm for *de novo* lncRNA and mRNA identification showing the number of transcripts identified at different steps (Supplemental Methods). At early steps filtering is performed for all

transcripts (lncRNA and mRNA) together. The filtering splits after the step removing transcripts with high exon repeat content*. The filtering pipeline identifies 6,249 lncRNA transcripts (corresponding to 1,591 lncRNA loci) and 132,864 mRNA transcripts (corresponding to 10,092 loci) in granulocytes.

B. Schematic representation of potential artifacts to be removed from the transcriptome assembly during filtering (step **). Top shows an annotated hypothetical mRNA (blue) and lncRNA (green) gene. Underneath is shown mRNA and lncRNA transcripts typically annotated in this study (alternative start or end sites of annotated transcripts was a frequent finding). Bottom shows transcripts (gray) annotated in this study that were filtered out as potential artifacts. These include wrong strand assignment of mRNAs (arising from poor strand-specificity of the RNA-seq), mRNA extension fragments, mRNA - lncRNA chimeric transcripts, and transcripts with unusually long exons (Supplemental Methods).

C. Validation of the filtering step that removes unusually long exons (part of step**). The bar plot shows the percentage of RefSeq and GENCODE v19 multi-exonic mRNAs (left) and lncRNAs (right) that fulfill (grey) or do not fulfill (black) the exon length filtering criteria. The vast majority of annotated multi-exonic mRNA and lncRNA transcripts pass this filtering step.

D. Validation of the step* to filter out repeat-rich transcripts. The bar plot shows the percentage of RefSeq and GENCODE v19 multi-exonic mRNAs (left) and lncRNAs (right) with <80% (pass the cut off - grey) or >=80% (do not pass the cut off - black) exonic content of repeats (http://www.repeatmasker.org/ [1]). The vast majority of annotated multi-exonic transcripts pass this filtering step.

E. Validation of the protein-coding potential estimation step***. The bar plot shows the percentage of RefSeq and GENCODE v19 multi-exonic mRNAs (left) and lncRNAs (right) identified as protein-coding (blue) and non-protein-coding (green). Nearly all mRNAs are identified as protein-coding, while most but not all, lncRNAs are identified as non-protein-coding.

Figure S2

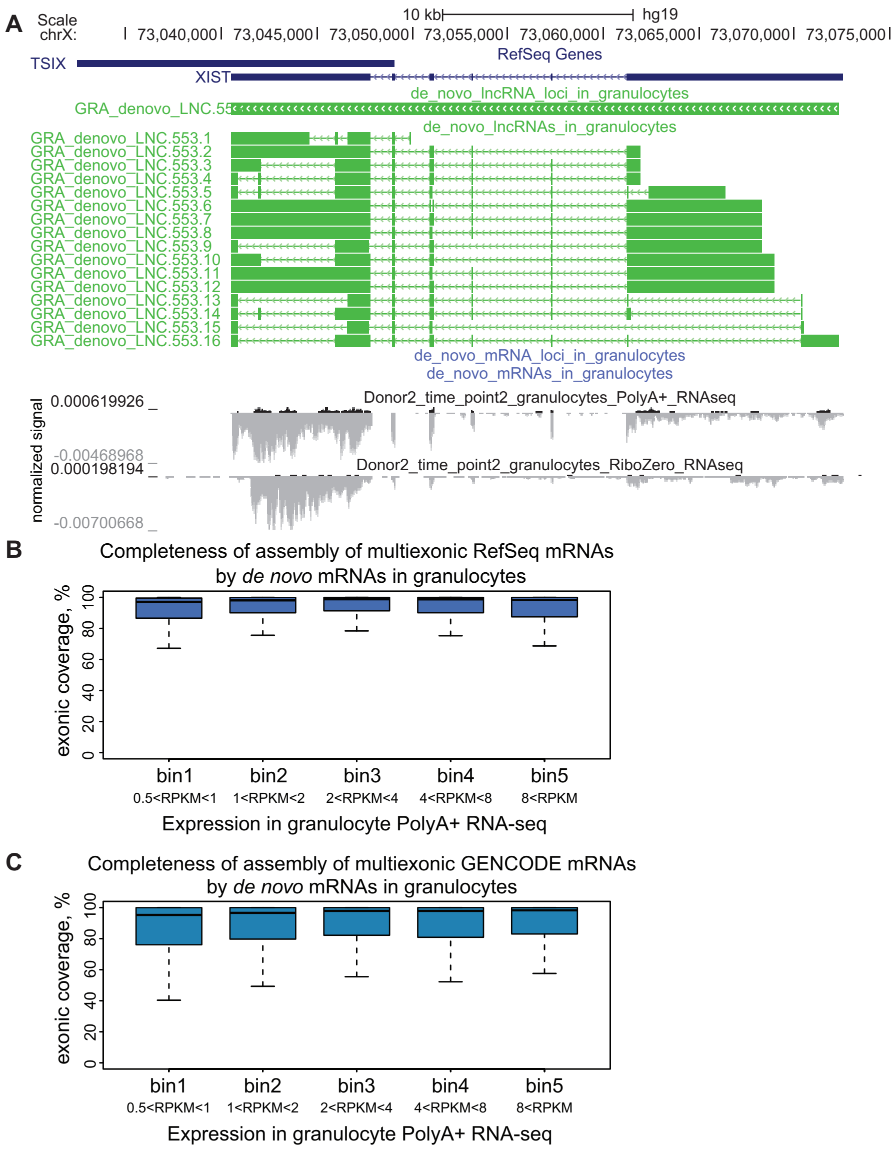

Figure S2. Quality of *de novo* transcriptome annotation in granulocytes

A. Assembling known lncRNAs - UCSC browser screen shot of an example of complete coverage of a well-known *XIST* lncRNA by *de novo* lncRNA annotation in granulocytes. From top to bottom: RefSeq gene annotation (the antisense *TSIX* lncRNA is cropped at the 5' end), *de novo* lncRNA loci annotation, *de novo* lncRNA transcript annotation (the pipeline annotates various isoforms of *XIST), de novo* mRNA loci and transcript annotation (empty in this genomic region), normalized PolyA+ RNA-seq signal for granulocytes from donor n2 (time point 2) and ribosomal depleted (Ribo Zero) RNA-seq of the same granulocyte sample (Additional File 2B). Both RNA-seq tracks show the presence of *XIST* and an absence of *TSIX* in granulocytes of Donor 2 (Additional File 2A).

B and C. Completeness of *de novo* assembly. Exonic coverage (Methods) of RefSeq (**B**) and GENCODE v19 (**C**) annotated mRNAs by *de novo* mRNA annotation in granulocytes. Genes are split into 5 bins, according to their average expression level in PolyA+ granulocyte RNA-seq samples used for transcriptome assembly (Additional File 2B), in order to account for the bias between expression level and assembly success. Median levels from left to right: **B**: 97.2, 98.1, 98.8, 98.7, 98.4; **C**: 95.3, 96.6, 97.9, 97.9, 98.2. Outliers are not displayed in the boxplots.

Figure S3

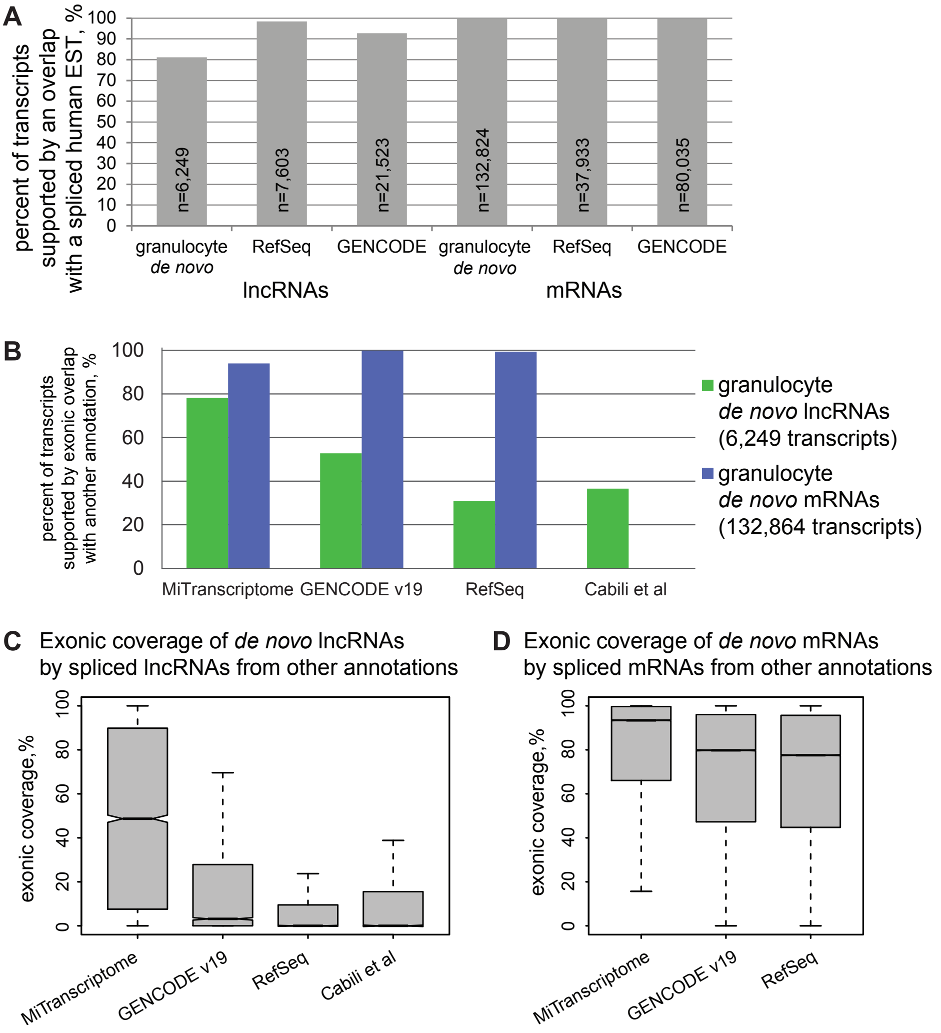

Figure S3. Validation of granulocyte *de novo* lncRNAs by overlap with other annotations

A. The majority of *de novo* lncRNAs annotated in granulocytes are supported by an overlap with an EST. The bar plot shows the percentage of transcripts that have a sense exonic overlap with a spliced EST (human EST database downloaded from UCSC) for (from left to right): *de novo* lncRNAs annotated in this study in granulocytes, lncRNAs annotated by RefSeq and GENCODE v19, *de novo* mRNAs annotated in this study in granulocytes, mRNAs annotated by RefSeq and GENCODE v19. Numbers inside bars indicate total number of transcripts in each annotation.
B. The majority of *de novo* lncRNAs in granulocytes are supported by an overlap with MiTranscriptome lncRNAs. The bar plot shows percentage of granulocyte *de novo* lncRNA (green) and mRNA (blue) transcripts supported by an exonic overlap with a multi-exonic lncRNA or mRNA respectively from MiTranscriptome, GENCODE v19, RefSeq and Cabili et al (only lncRNAs) annotations [2-5].
C. MiTranscriptome provides best exonic coverage for granulocyte *de novo* lncRNAs. The box plot shows percent exonic coverage (Methods) of granulocyte *de novo* lncRNAs by multi-exonic lncRNAs from MiTranscriptome, GENCODE v19, RefSeq and Cabili et al annotations. Number of granulocyte *de novo* lncRNA transcripts examined in each box – 6,249. Median values left to right: 48.7, 3.2, 0, 0.
D. Granulocyte *de novo* mRNAs are nearly fully exonically covered by MiTranscriptome and public annotations. The box plot shows percent exonic coverage of granulocyte *de novo* mRNAs by multi-exonic mRNAs from MiTranscriptome, GENCODE v19 and RefSeq annotations. Number of granulocyte *de novo* mRNA transcripts examined in each box – 132,864. Median values left to right: 93.4, 79.8, 77.6. Remarks: Outliers are not displayed in the box plots.

Figures S4-S8. Validation of granulocyte *de novo* lncRNAs by cloning

(N.B., Figures S4-S8 have a common legend)

Cloning result for each lncRNA locus *de novo* annotated in granulocytes is represented by a UCSC browser screen shot. Each screen shot contains (from top to bottom): chromosome scale, chromosome coordinates, RefSeq gene annotation, granulocyte *de novo* mRNA loci annotation obtained in the study (blue), granulocyte *de novo* lncRNA loci annotation obtained in the study (green), *de novo* lncRNA transcripts constituting the given lncRNA locus (green), RT-PCR primers used to amplify the targeted full length lncRNA transcript (Additional File 2F), BLAT alignments of Sanger sequences obtained after cloning the targeted lncRNA transcript (black). For the loci containing several isoforms, those used for primer design are marked with (*). RT-PCR and cloning procedure do not preserve strandness of the transcript and, thus, the BLAT alignments' strands do not necessarily match the corresponding lncRNA strand. Sanger sequencing results ("_T7" tag for T7 primer used) from some cloned products could not cover the full-length transcript and these products were sequenced from the other end ("_SP6" tag for SP6 primer used). Red lines in BLAT alignments indicate mismatches between the UCSC reference genome sequence and the sequence of the aligned Sanger sequences, the width of a red line does not scale with the size of the mismatch.

Figure S4 (legend – p.8)

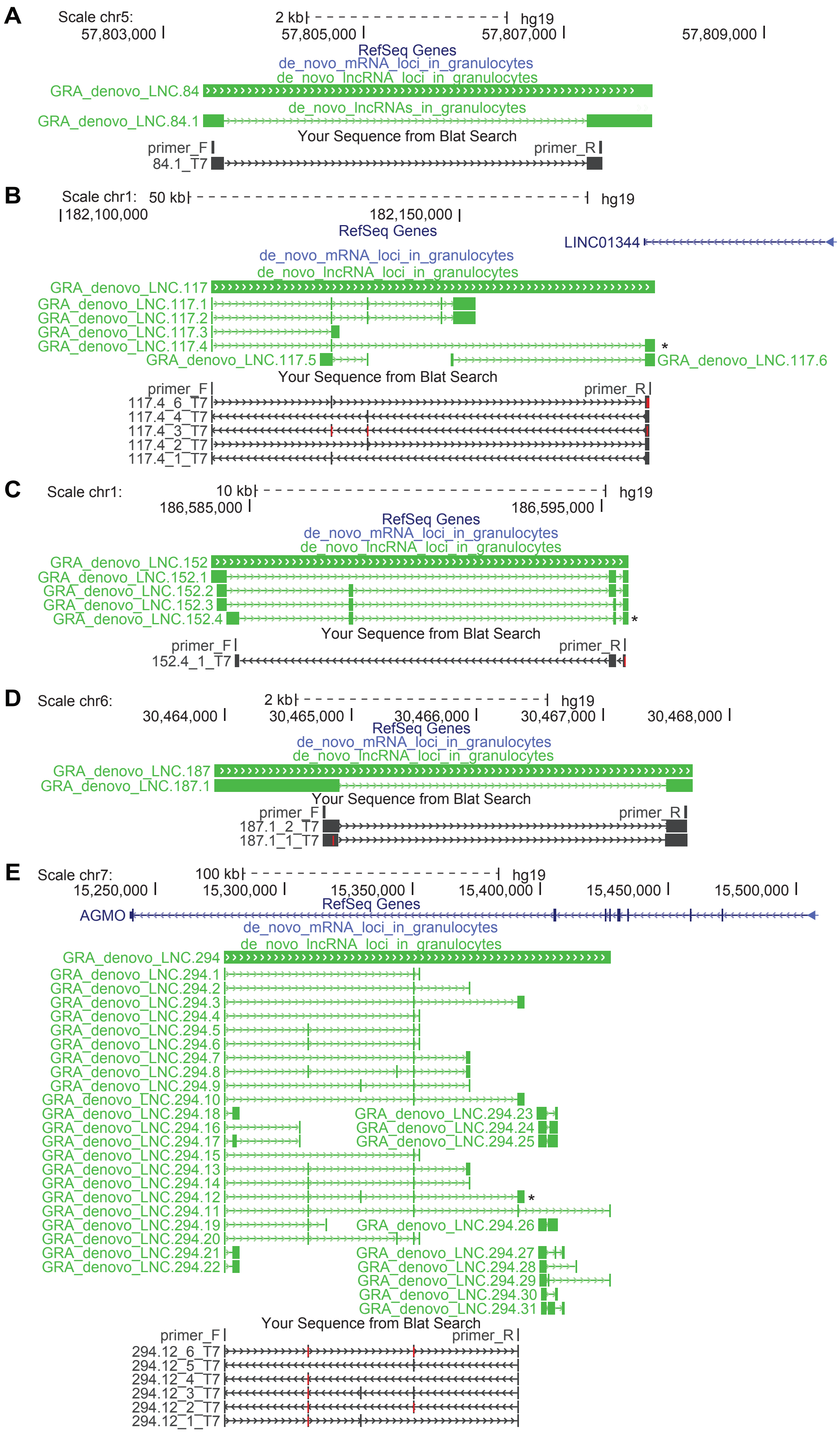

Figure S5 (legend – p.8)

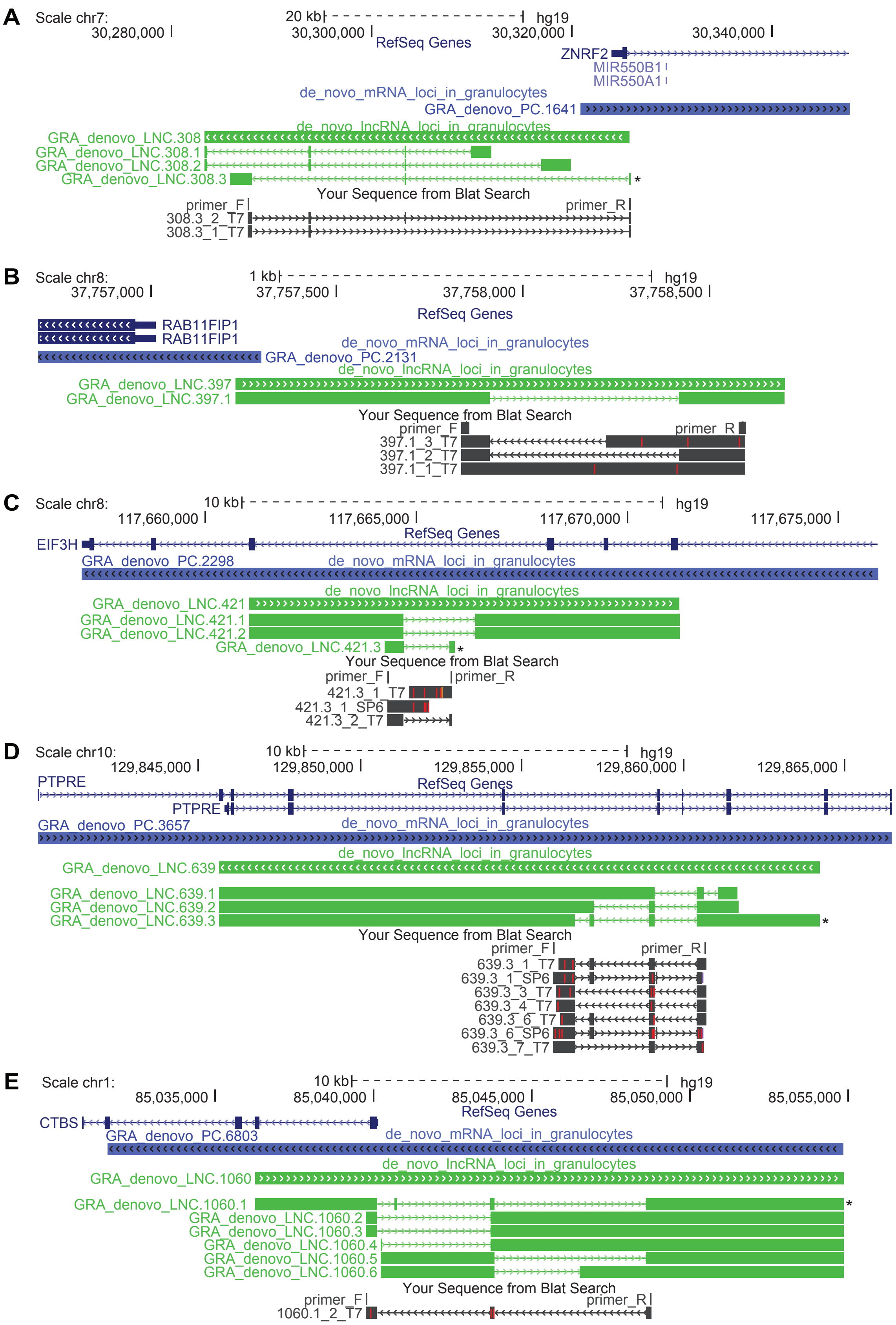

Figure S6 (legend – p.8)

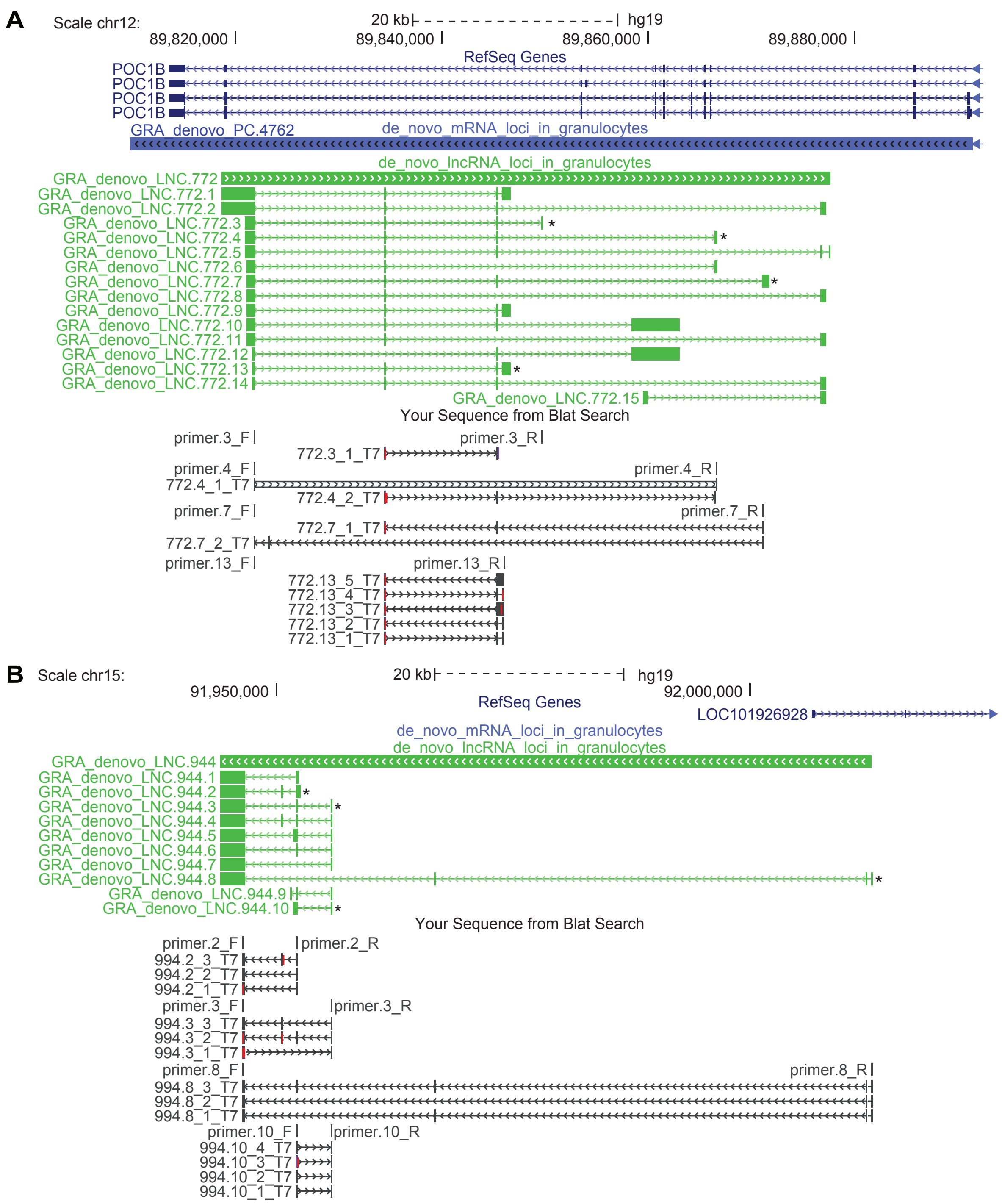

Figure S7 (legend – p.8)

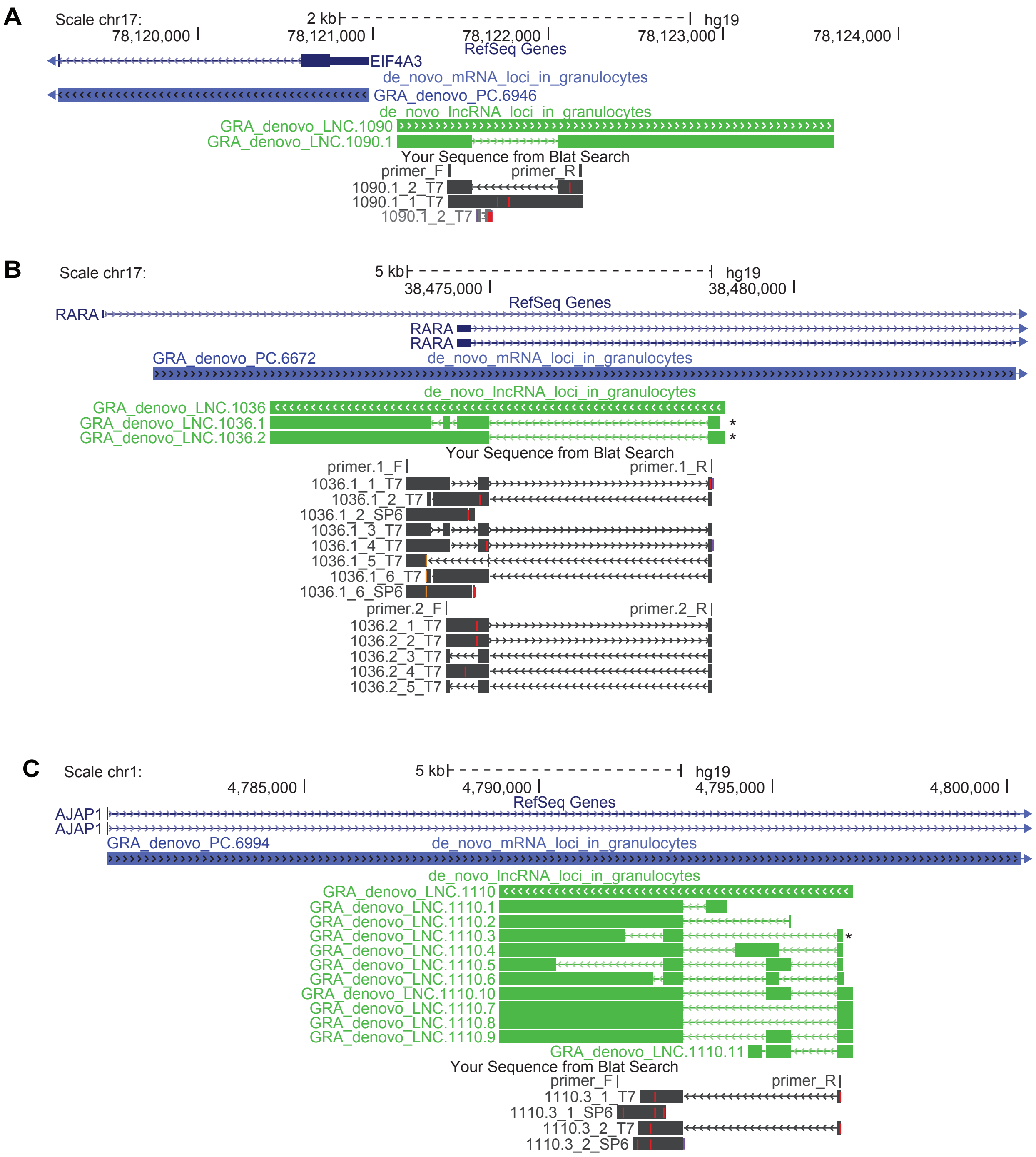

Figure S8 (legend – p.8)

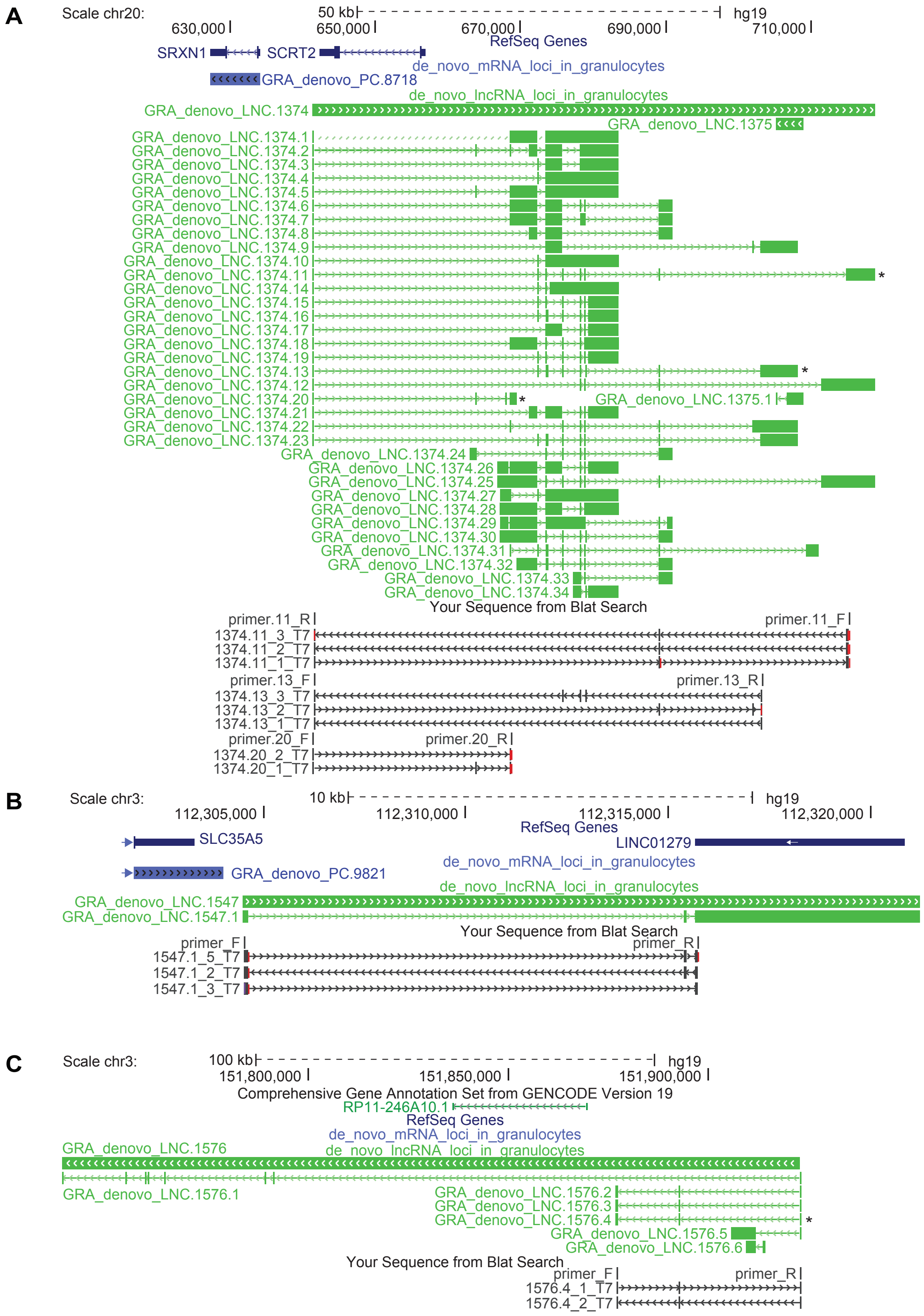

Figure S9

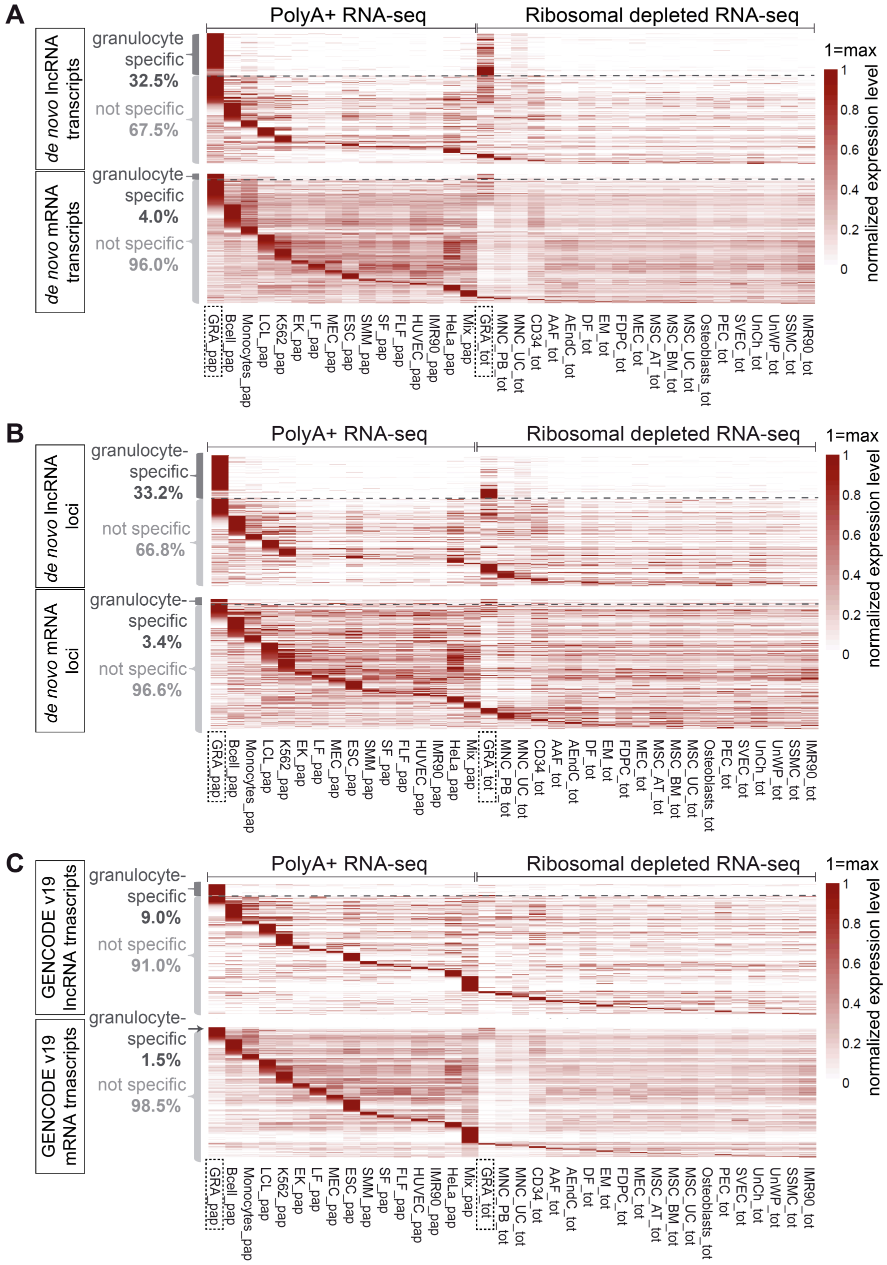

Figure S9. Granulocyte specificity of granulocyte *de novo* and GENCODE annotated lncRNAs and mRNAs

Each heat map represents expression levels of **(A)** *de novo* granulocyte transcripts - top: lncRNAs (5,936 transcripts) in the heat map), bottom: mRNAs (132,813 transcripts in the heat map) **(B)** *de novo* granulocyte loci - top: lncRNA loci (1,218 loci in the heat map), bottom: mRNA loci (9,946 loci in the heat map), **(C)** GENCODE v19 multi-exonic transcripts - top: lncRNAs (13,489 transcripts in the heat map), bottom: mRNAs (77,529 transcripts in the heat map). Heat maps show expression level RPKM (reads per kilobase of transcript per million reads mapped) of each transcript/locus in 34 public strand-specific human RNA-seq samples (Additional File 2H) normalized by maximal expression among all samples (maximum is set to 1). GRA_pap (dotted box): average expression level among 17 PolyA+ RNA-seq samples from 10-donors, GRA_tot (dotted box): average expression level among 21 ribosomal depleted RNA-seq samples from 7-donors (Additional File 2B). pap: polyA+ RNA (15 samples), tot: ribosomal depleted RNA (19 samples). Above dashed line: transcripts/loci defined as "granulocyte-specific" (dark grey brace) show maximal expression in the GRA_tot or GRA_pap samples and ≥3-fold lower expression in all other samples. Below dashed line: transcripts/loci not meeting these criteria and called not specifically expressed in granulocytes (light grey brace). Only transcripts/loci expressed (RPKM>0.2) in at least one of the samples were analyzed.

Figure S10

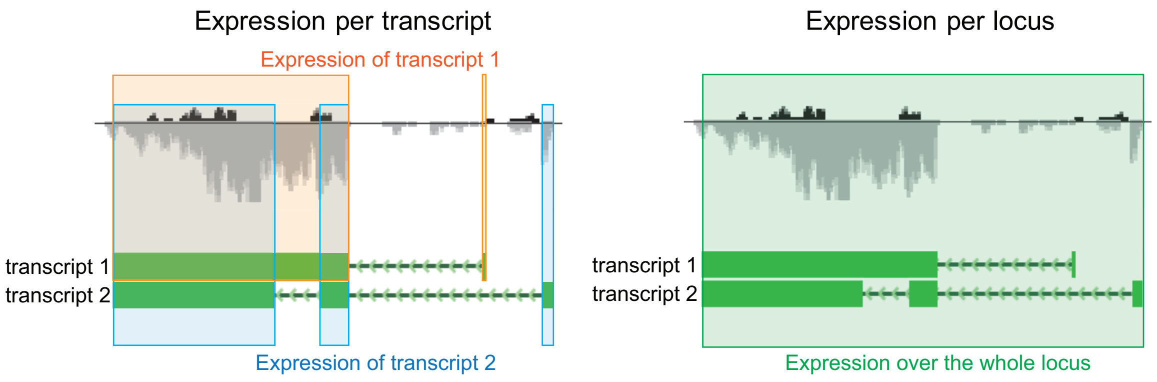

Figure S10. Illustration of expression calculation per transcript and over whole locus

Throughout the study expression of a transcript is calculated as RPKM of only its exons using BED12 transcript annotation which provides information on position of exons (left), thus only the reads that map to exons are taken into account. Locus expression is calculated for the whole locus, thus all the reads, both exonic and intronic, are counted (right).

Figure S11

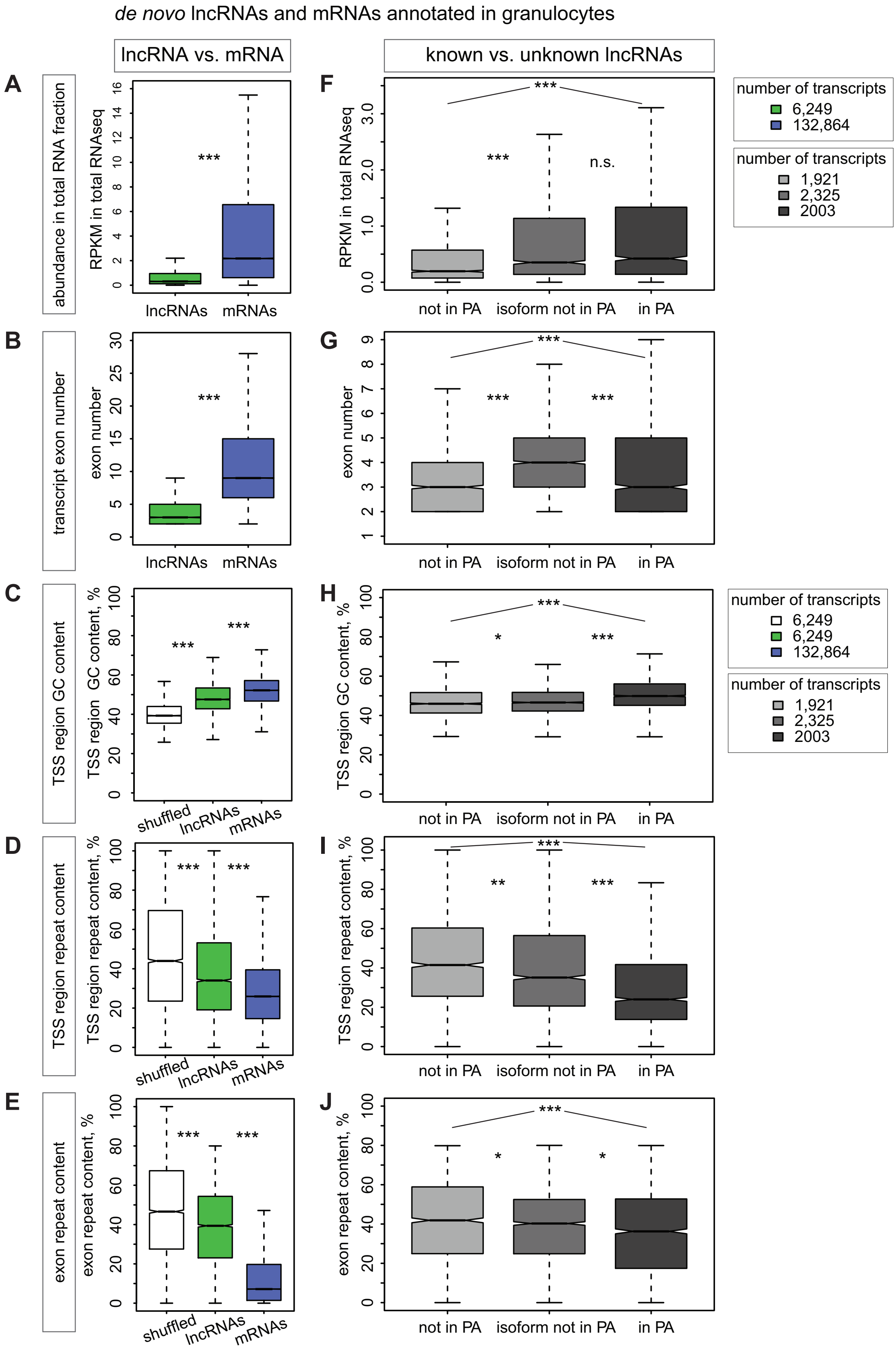

Figure S11. Features that distinguish granulocyte lncRNAs from mRNAs also distinguish novel granulocyte lncRNAs from publicly annotated lncRNAs

A. Abundance in total RNA fraction of granulocyte *de novo* lncRNA and mRNA transcripts was calculated as an average from all 21 available Ribosomal Depleted RNA-seq samples. Exon number (**B**), percent GC content of transcription start site (TSS) region (TSS +/- 1.5 kb) (**C**), percent repeat coverage of TSS region (**D**) and exons (**E**) of *de novo* annotated lncRNAs (green) and mRNA (blue) transcripts. Abundance in total RNA fraction (**F**), exon number (**G**), percent GC content of TSS region (**H**), percent repeat coverage of TSS region (**I**) and exons (**J**) of the three classes of *de novo* lncRNAs (described in Fig. 2A). **C**, **D** and **E**: "shuffled" control (white) is added to the box plots. Shuffled control represents random regions in the genome using *bedtools shuffle*. C and D: granulocyte *de novo* lncRNA TSS regions (n=6,249) were shuffled across the genome, E: granulocyte *de novo* lncRNA transcripts (n=6,249) were shuffled across the genome.
Remarks to boxplots: green: all *de novo* lncRNAs, blue: all de novo mRNAs, light gray: 'not in PA' lncRNA transcripts, medium gray: 'isoform not in PA' lncRNA transcripts, dark gray: 'in PA' lncRNA transcripts (PA: public annotations). The numbers of transcripts in each box are indicated top right. *** - $p<10^{-10}$, ** - $p<10^{-5}$, * - $p<0.01$, n.s. – $p>0.01$. The box plots display the full population but p-values are calculated using Mann–Whitney U test with equalized sample size (Methods). Median values from left to right: **A**: 0.31, 2.18; **B**: 3, 9; **C**: 47.6, 52.2; **D**: 34.0, 25.9; **E**: 39.4, 7.2; **F**: 0.20, 0.35, 0.42; **G**: 3, 4, 3; **H**: 46.0, 46.6, 49.9; **I**: 41.5, 35.1, 24.0; **J**: 41.9, 40.3, 36.3. Outliers are not displayed in the box plots.

Figure S12

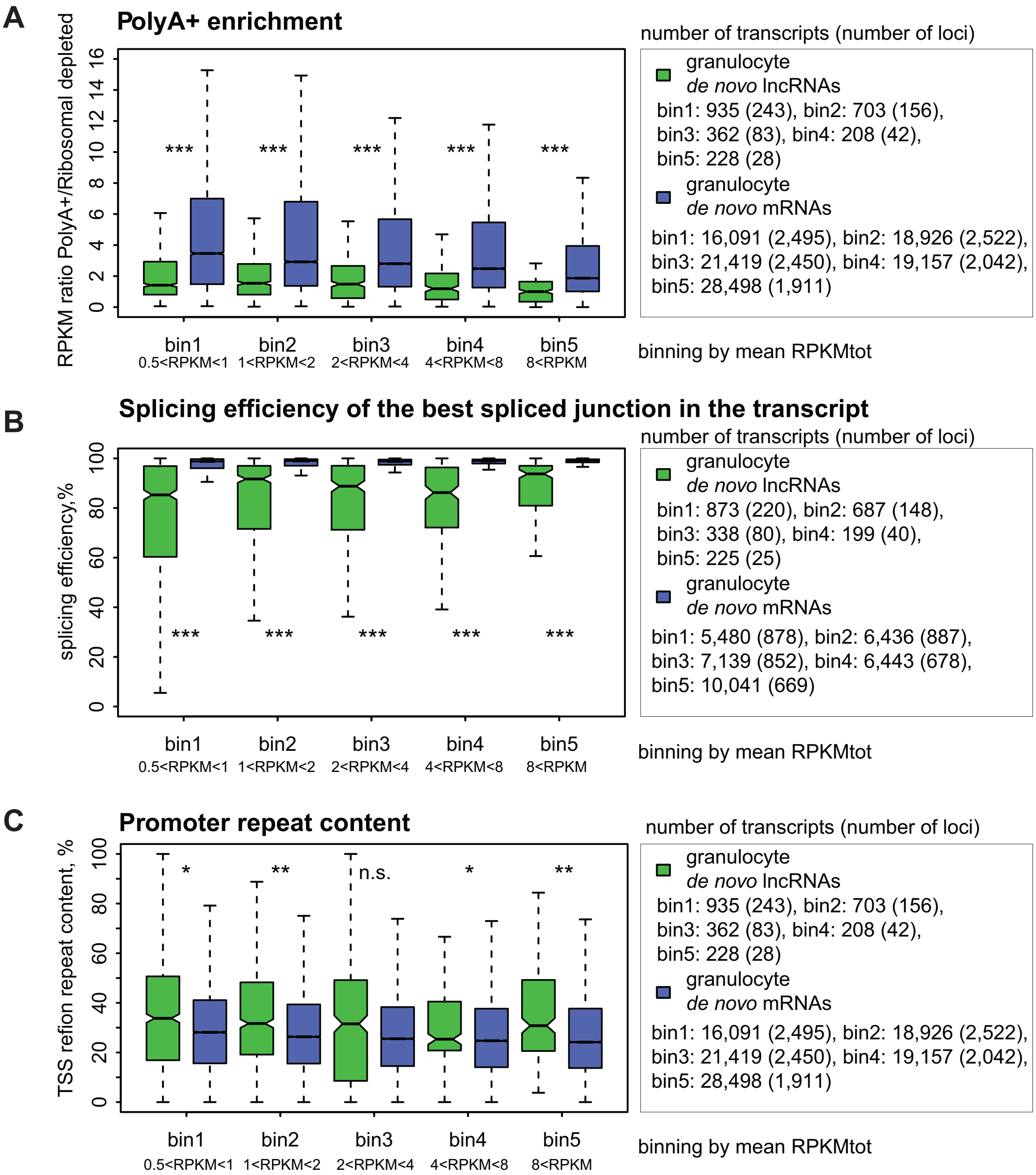

Figure S12. Difference between mRNAs and lncRNAs is persistent independently of expression level

A. PolyA+ enrichment (calculated as in Figure 2E) of granulocyte *de novo* lncRNA and mRNA transcripts split into expression bins. Median values in boxes from left to right: 1.4, 3.5, 1.5, 2.9, 1.5, 2.8, 1.2, 2.5, 1.0, 1.9.

B. Splicing efficiency (calculated as in Figure 2F) of granulocyte *de novo* lncRNA and mRNA transcripts split into expression bins. Median values in boxes from left to right: 85.2%, 98.8%, 91.7%, 99.0%, 88.8%, 98.8%, 86.2%, 99.0%, 93.8%, 99.2%.

C. Percent repeat coverage of TSS region (TSS +/- 1.5 kb) of granulocyte *de novo* lncRNAs and mRNA transcripts split into expression bins. Median values in boxes from left to right: 33.8%, 28.1%, 31.7%, 26.3%, 31.5%, 25.5%, 25.4%, 24.7%, 30.8%, 24.2%.

Remarks to boxplots: The numbers of transcripts in each box are indicated on the right. Number in brackets indicates number of loci the transcripts in each box initiate from. *** - $p<10^{-10}$, ** - $p<10^{-5}$, * - $p<0.01$, n.s. – $p>0.01$. The box plots display the full population but p-values are calculated using Mann–Whitney U test with equalized sample size (Methods). Outliers are not displayed in the box plots. “mean RPKMtot” – average transcript RPKM in 21 ribosomal depleted granulocyte RNA-seq samples.

Figure S13

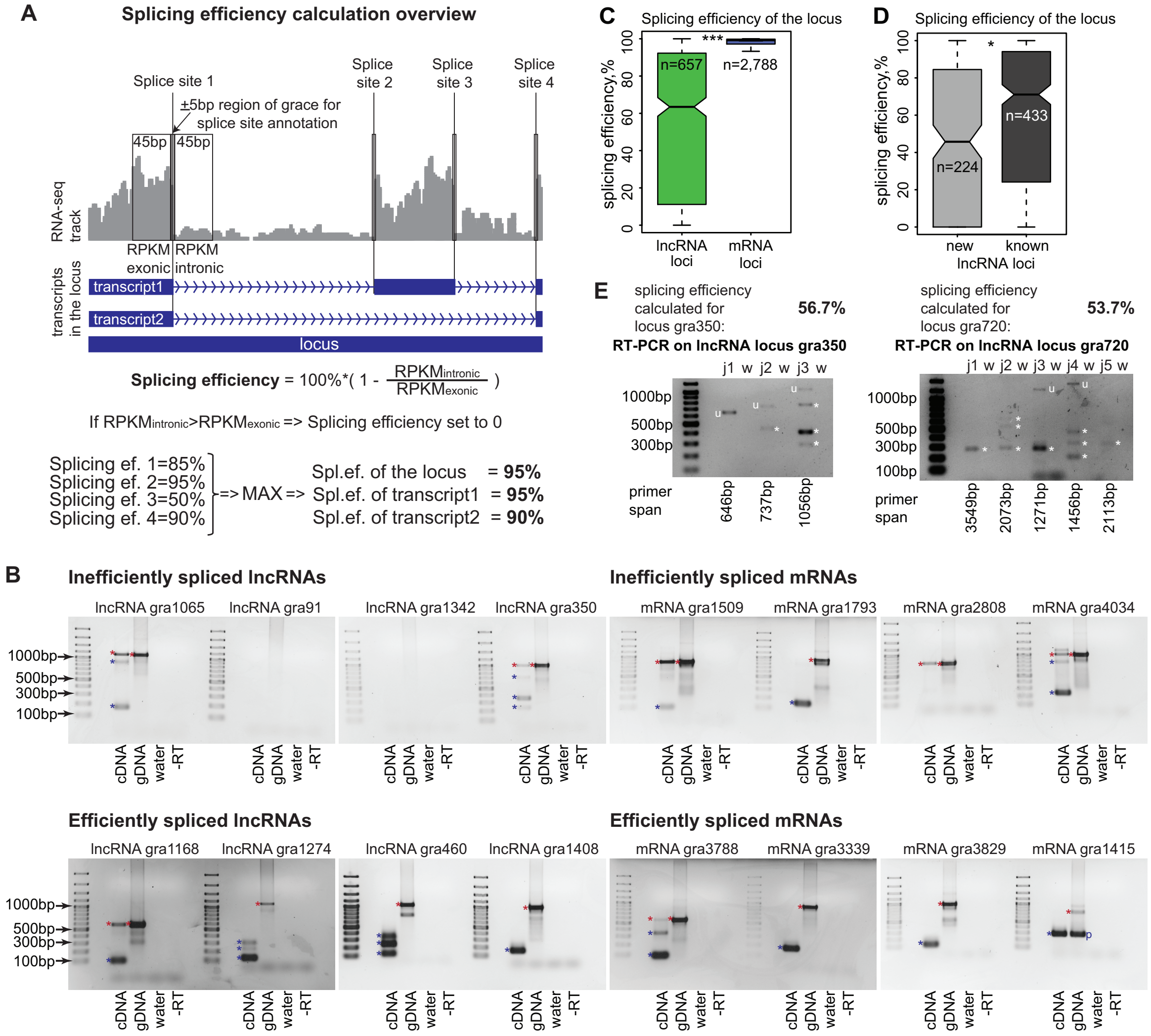

spliced	type	locus	Forw ard primer seq 5'-3'	Reverse primer seq 5'-3'	unspliced product size
inefficiently	lncRNA	GRA_denovo_LNC.1065	TTCTCATGGGAGCAGCTGTA	AGGGGGAGATGTCACCTTTT	1098bp
inefficiently	lncRNA	GRA_denovo_LNC.91	TTCCGTGATGTTTGCATTTC	AAAGGCAAACTGCTGAATCC	1014bp
inefficiently	lncRNA	GRA_denovo_LNC.1342	CAGTGTTTCCCATTTCCTGA	TTGCAATACATTCCACTCTGTG	883bp
inefficiently	lncRNA	GRA_denovo_LNC.350	CCAACCGAGGATGTAAGAGG	TGGAAGATTCAACTGGCAGA	1002bp
inefficiently	mRNA	GRA_denovo_PC.1509	ACGGCCTGGATTCAGAGACT	ACAGACACCCTTTGGACCAC	821bp
inefficiently	mRNA	GRA_denovo_PC.1793	GTCCTCGAAAGTCACCATCC	AGCAGTTCCTCACCATCCTG	944bp
inefficiently	mRNA	GRA_denovo_PC.2808	CTCTGCAGCAAGAAATGAGG	CTCTTTCTTTGAGGGAACACC	767bp
inefficiently	mRNA	GRA_denovo_PC.4034	CAGCCTACCTGTCCATGTCC	ACGGTAATCCAGCGAGGACT	1024bp
efficiently	lncRNA	GRA_denovo_LNC.1168	TCAAAAGCCGTTGAGACACA	GGAGGCATCCTCTGTCAAGA	572bp
efficiently	lncRNA	GRA_denovo_LNC.1274	CGCAGGGAAACAGTTCTCAT	TCCAGCTGACTTGCATTCTG	1099bp
efficiently	lncRNA	GRA_denovo_LNC.460	AACTCAAGCAGAAGAGCAGTTG	GCACCGGAGACTGAAAGAAG	1043bp
efficiently	lncRNA	GRA_denovo_LNC.1408	GAGGCTGAGCAAGGGACA	TCTCAGAAATCTGTGAGCTGTG	993bp
efficiently	mRNA	GRA_denovo_PC.3788	CTCGCCAAAAACTTCACCTT	AGGAGAGCAAGACACACTTCC	562bp
efficiently	mRNA	GRA_denovo_PC.3339	GATACTGCTGCTTGCCTTCC	GAAGGATTGACACCAACCTCA	902bp
efficiently	mRNA	GRA_denovo_PC.3829	ACTGCTCCAAGCACCTCCT	TAGACCCAAGGCTGTCACAC	973bp
efficiently	mRNA	GRA_denovo_PC.1415	CCTCTTCGGGGAGATGTAGC	TCCACTGCCTTACGTTTTGG	772bp

Figure S13. Splicing efficiency calculation

A. Overview of splicing efficiency calculation (Methods). Shown is an illustrative RNA-seq signal track of a genomic locus. Splicing efficiency of each splice site in the locus is calculated based on the ratio between intronic and exonic RNA-seq signal at this splice site. RNA-seq signal is calculated as RPKM of 45bp exonic and intronic regions surrounding the splice site. 45bp are positioned 5bp away from the precise splice site position to accommodate for potential imprecise splice site annotation. Splicing efficiency is calculated with the given formula. In case of intronic signal exceeding exonic

signal, splicing efficiency is set to 0. Maximal splice site splicing efficiency is taken to estimate splicing efficiency of each transcript, as well as of the whole locus.

B. RT-PCRs to test splicing efficiency calculation (see Supplemental Methods). RT-PCR result for one junction (junction length<1500bp) of eight inefficiently spliced (upper row gel pictures, see Supplemental Methods) and efficiently spliced (lower row gel pictures, see Supplemental Methods) *de novo* granulocyte transcripts (4xlncRNAss and 4xmRNAs). List of assayed lncRNA and mRNA transcripts and primers to amplify their short junction is given in the table below. Size of the expected PCR product amplifying the unspliced junction is given in the right-most column of the table. Each primer pair was used with granulocyte cDNA sample and three control samples: genomic DNA (from the same granulocyte sample) - to test efficiency of the primers when amplifying the long unspliced isoform, water - to test PCR reaction contamination, -RT (no reverse transcriptase added when preparing cDNA) control – to test for genomic DNA contamination in the cDNA sample. Red stars on the left from the band indicate the expected band corresponding to the unspliced product. Blue stars on the left from the band indicate spliced products. lncRNA gra91 and lncRNA gra1342 – absence of a band in gDNA indicates inefficiency of primers. mRNA gra1415 – band marked “p” might indicate the presence of a pseudogene corresponding to the assayed protein-coding gene somewhere in the genome. Overall, the RT-PCR test validates our splicing efficiency calculation with transcripts identified as unspliced showing an abundant unspliced isoform (5 out of 6 with one (mRNA gra1793) exception) and transcripts identified as spliced showing no unspliced isoform (6 out of 8 with two exceptions (lncRNA gra1168 and mRNA mRNA gra3788) showing some unspliced product signal, however it is much fainter than the band formed by the spliced products). Note that, as described in (**A**), we assign a transcript with the splicing efficiency of its best spliced splice site, whereas the RT-PCR assay allows to assess splicing efficiency of only the short (<1.5kb) splice sites, which might explain the slight discrepancy in the results presented in (**B**).

C. Analysis of splicing efficiency of the whole locus confirms reduced splicing efficiency of granulocyte de novo lncRNAs compared to mRNAs. Boxplot shows splicing efficiency (as described in A, Methods) of *de novo* lncRNA (green) and mRNA (blue) loci annotated in granulocytes. Splicing efficiency of each splice site was calculated (Methods) and the efficiency of the most efficiently spliced transcript (i.e. most efficiently spliced site of all the transcripts) in each locus is plotted. Median values: lncRNAs: 63.46%, mRNAs: 99.07%.

D. New lncRNA loci are less efficiently spliced than known lncRNA loci. Boxplot shows splicing efficiency (as described in A, Methods) of new (light grey) and known (dark grey) (as described in Fig. 2A) granulocyte *de novo* lncRNA loci. Splicing efficiency calculated as in (**C**). Median values: new loci: 45.75%, known loci: 71.06%.

E. Two illustrative examples of exon spanning RT-PCR amplifying a continuous unspliced isoform. Gel electrophoresis of RT-PCR over splice junctions in granulocyte *de novo* lncRNA loci 350 and 720 (See Additional File 2E for primer sequence and junction genomic position and Additional File 3 for locus annotation). Above the gel picture, splicing efficiency calculated from RNA-seq data as described in (**A**) is shown. **j**: junction number, **w**: PCR water control. *: bands corresponding to spliced products, **u**: bands corresponding to the unspliced product. Primer span: the genomic span of PCR primers corresponding to the length of the unspliced PCR product.

Remarks to boxplots: Numbers inside boxes indicate number of loci displayed in each box. The box plots display the full population but p-values are calculated using Mann–Whitney U test with equalized sample size (Methods). *- p<0.01, *** - $p<10^{-16}$. Outliers are not displayed.

Figure S14

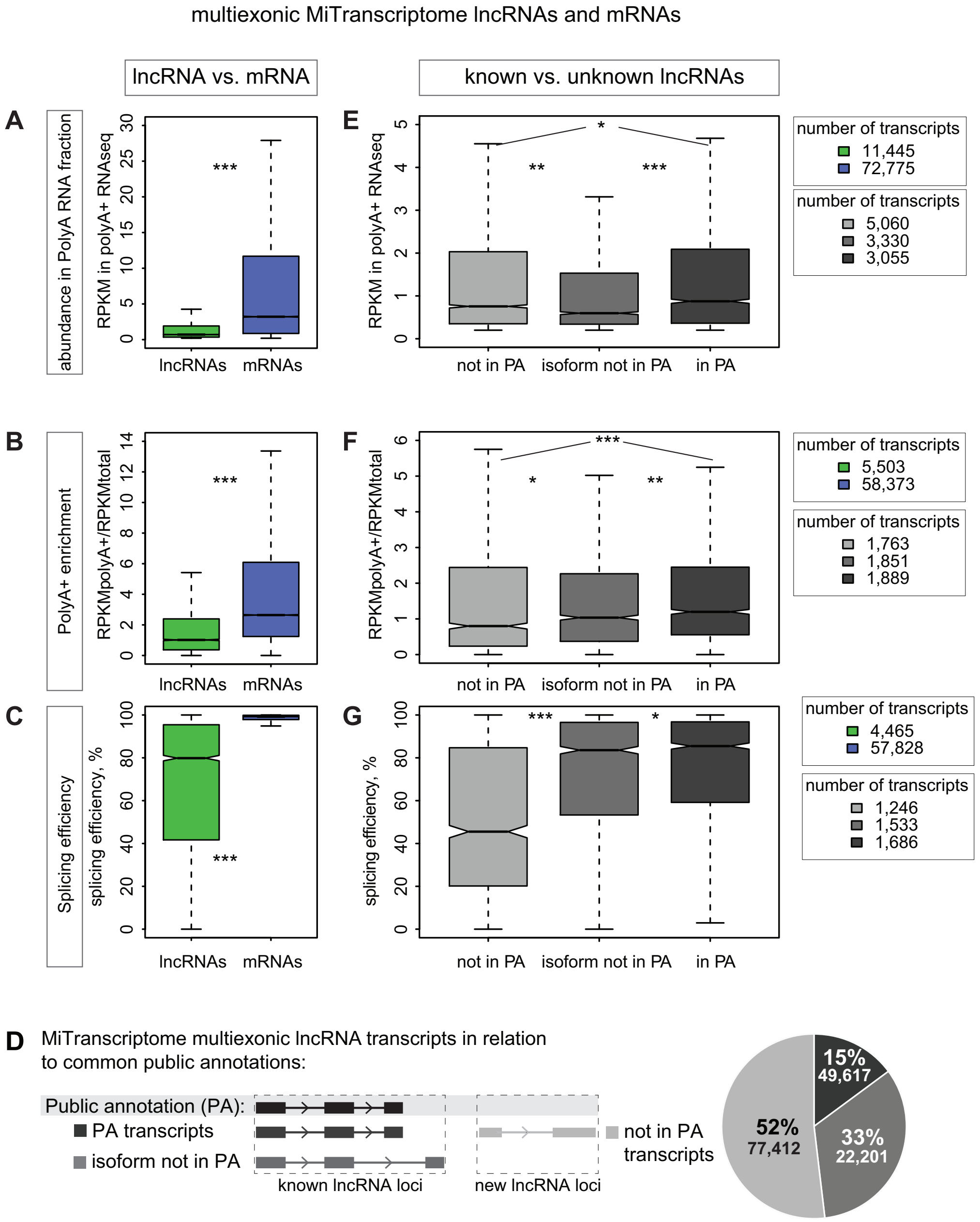

Figure S14. Analyzing features of MiTranscriptome lncRNAs and mRNAs confirms that difficult to identify lncRNAs are more different from mRNAs than publicly annotated lncRNAs

A. Average expression level (RPKM) of multi-exonic MiTranscriptome lncRNA (green) and mRNA (blue) transcripts in granulocyte PolyA+ RNA-seq samples produced in the study. Only transcripts with detectable expression are plotted (average RPKM>0.2).

B. PolyA+ enrichment of multi-exonic MiTranscriptome lncRNA (green) and mRNA (blue) transcripts as described in Fig. 2E. Only transcripts detected in total granulocyte RNA-seq data (average RPKM among 21 samples >0.2) are analyzed.

C. Splicing efficiency of multi-exonic MiTranscriptome lncRNA (green) and mRNA (blue) transcripts expressed in granulocytes. Splicing efficiency was calculated using ribosomal-depleted

RNA-seq from 7 donors (time points pooled to increase the coverage) (Methods). The splicing efficiency of the most efficiently spliced site in each transcript is plotted.

D. Distribution of multi-exonic MiTranscriptome lncRNA transcripts according to their coverage in the 3 commonly used public annotations as described in Fig. 2A for granulocyte *de novo* lncRNAs.

E. Expression level of the 3 types of multi-exonic MiTranscriptome lncRNA transcripts in granulocyte PolyA+ RNA-seq. Publicly annotated transcripts show the highest expression level.

F. PolyA+ enrichment of the 3 types of multi-exonic MiTranscriptome lncRNA transcripts.

G. Splicing efficiency of 3 types of multi-exonic MiTranscriptome lncRNA transcripts described in (**D**). The splicing efficiency of the most efficiently spliced site in each transcript is plotted.

Remarks to boxplots A, B, C, E, F and G: Numbers on the right indicate the numbers of transcripts analyzed in each boxplot. The box plots display the full population but p-values are calculated using Mann–Whitney U test with equalized sample size (Methods). *** - $p<10^{-10}$, ** - $p<10^{-5}$, * - $p<0.01$, n.s. – $p>0.01$. Median values from left to right: **A**: 0.71, 3.21; **B**: 1.02, 2.64; **C**: 79.9, 99.2; **E**: 0.76, 0.60, 0.87; **F**: 0.80, 1.04, 1.20; **G**: 45.5, 83.6, 85.5. Outliers are not displayed in the box plots. Numbers on the right represent the number of transcripts in each box.

Figure S15

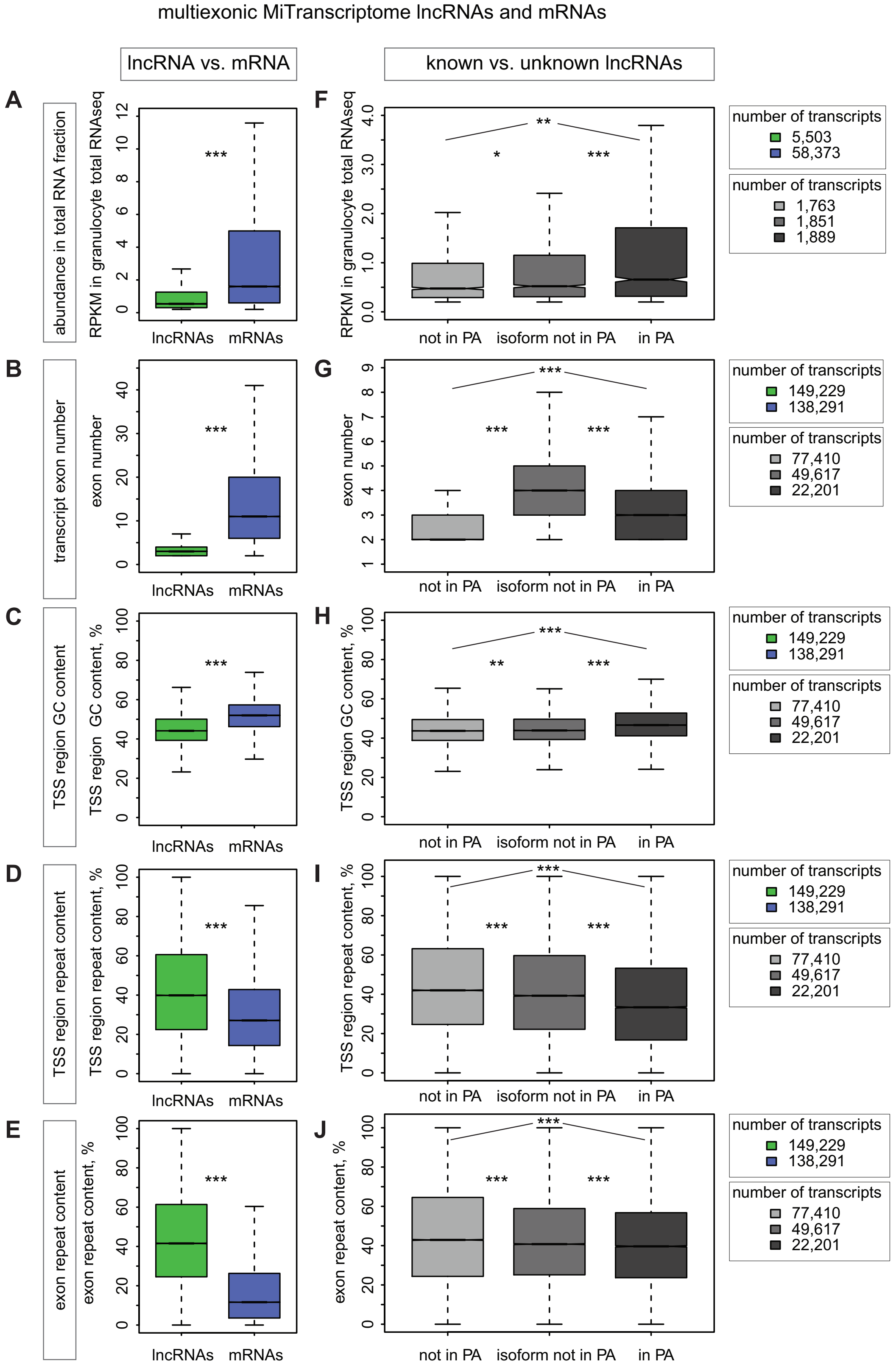

Figure S15. Additional features that distinguish MiTranscriptome lncRNAs from mRNAs, and newly identified from publicly annotated lncRNAs

A. Abundance in total RNA fraction of multi-exonic MiTranscriptome lncRNA and mRNA transcripts was calculated as average from all 21 available Ribosomal Depleted RNA-seq samples.

Only MiTranscriptome transcripts expressed in granulocyte total RNA-seq dataset (average RPKM>0.2) are plotted. Exon number (**B**), percent GC content of TSS region (TSS +/- 1.5 kb) (**C**), percent repeat coverage of TSS region (**D**) and exons (**E**) of multi-exonic MiTranscriptome lncRNAs and mRNA transcripts. Abundance in total RNA fraction in granulocytes (**F**), exon number (**G**), percent GC content of TSS region (**H**), percent repeat coverage of TSS region (**I**) and exons (**J**) of the three classes of MiTranscriptome lncRNA as described in Figure S14D. Remarks: green - multi-exonic MiTranscriptome lncRNAs, blue – multi-exonic MiTranscriptome mRNAs, light gray – “not in PA” lncRNA transcripts, medium gray – “isoform not in PA” lncRNA transcripts, dark gray – “PA” lncRNA transcripts. Numbers of transcripts in each box of the boxplots are indicated on the right. *** - $p<10^{-10}$, ** - $p<10^{-5}$, * - $p<0.01$, n.s. – $p>0.01$. The box plots display the full population but p-values are calculated using Mann–Whitney U test with equalized sample size (Methods). Median values from left to right: **A**: 0.54, 1.60; **B**: 3, 11; **C**: 44.13, 52.00; **D**: 39.87, 27.10; **E**: 41.55, 11.63; **F**: 0.48, 0.52, 0.66; **G**: 2, 4, 3; **H**: 43.70, 43.90, 46.63; **I**: 42.00, 39.27, 33.37; **J**: 42.91, 40.77, 39.63. Outliers are not displayed in the box plots.

Figure S16

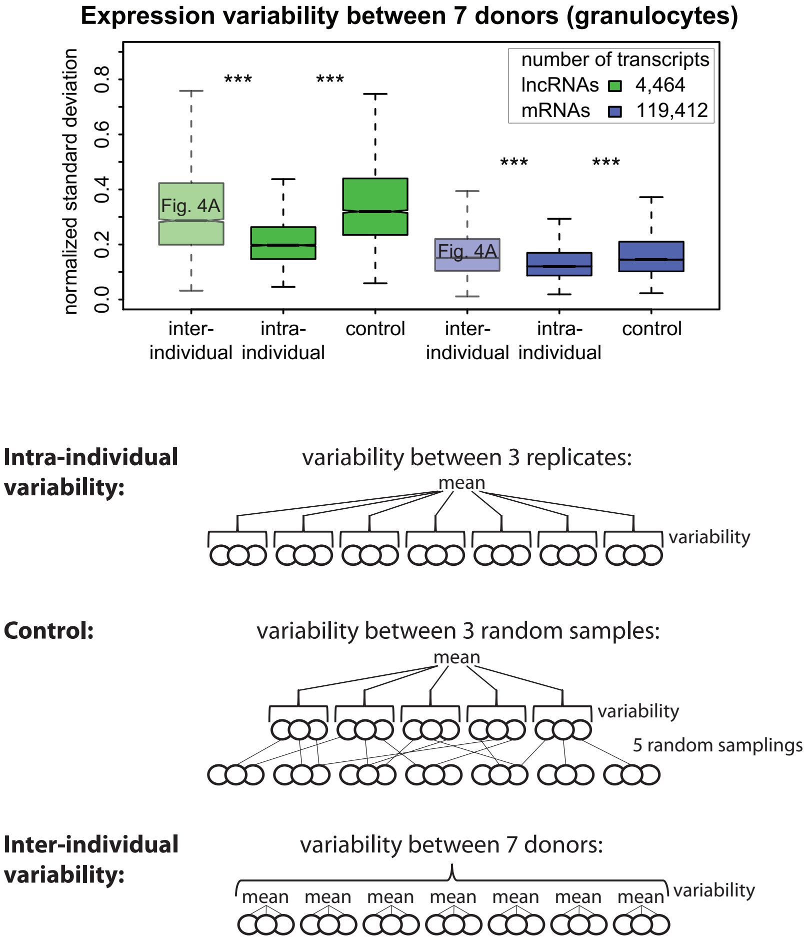

Figure S16. Intra-individual variability is significantly lower than inter-individual variability for both lncRNAs and mRNAs in granulocytes

The boxplot shows values of different types of expression variability of granulocyte *de novo* annotated lncRNA (green) and mRNA (blue) transcripts in granulocyte total RNA-seq dataset. We calculated intra-individual variability (between 3 replicates of each donor), compared it to inter-individual variability (between 7 donors, data displayed in Fig. 4A, here indicated as transparent boxes). We then controlled for the reduced sample size in intra-individual variability calculation (3 samples for intra-individual vs. 7 samples for inter-individual variability) by randomly sampling 3 replicates and asking if the inter-individual variability observed is reduced with the reduced sample size. Graphical representation of calculation of the three variability types plotted is displayed below the boxplot. From top to bottom: Intra-individual variability (variability "between 3 replicates"): standard deviation was calculated for each donor by calculating standard deviation of 3 replicates and normalized by the mean of the 3 replicates, 7 normalized standard deviation values from 7 donors were then averaged to give intra-individual variability for each transcript. Control (variability between 3 random replicates): 3 replicates were randomly picked from the 21 (7 donors x 3 replicates) samples, normalized standard deviation was calculated for the 3 samples, the random sampling was performed 5 times and the average of 5 normalized standard deviations was calculated. Inter-individual variability (variability between 7 donors): calculated as described for Fig. 4A (Results).
Remarks: Chr. X and Y were discarded from the analysis. *** - $p<10^{-16}$. P-values are calculated using Mann–Whitney U test. Numbers in the boxplot legend indicate number of transcripts analyzed. Median values of boxes left to right: lncRNAs: 0.29, 0.20, 0.32, mRNAs: 0.15, 0.12, 0.15. Outliers are not displayed in the box plot.

Figure S17

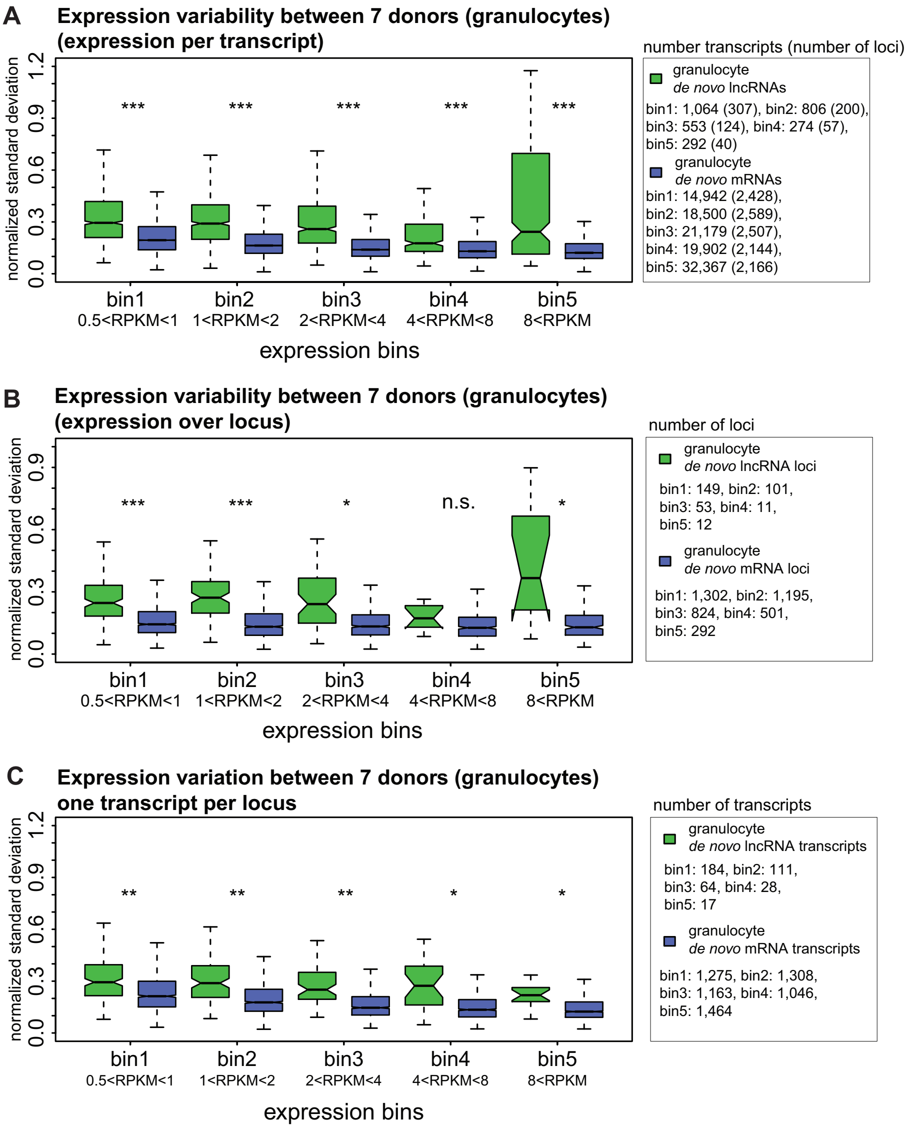

Figure S17. *De novo* lncRNAs are more variable than mRNAs independently of generally lower lncRNA expression level

The phenomenon of increased expression variability of lncRNAs compared to mRNAs is not biased to the difference in absolute expression level between lncRNAs and mRNAs.

A. and **B.** Normalized standard deviation of *de novo* granulocyte lncRNA (green) and mRNA (blue) transcripts **(A)** or loci **(B)** expression between granulocytes from 7 donors, split into 5 expression bins according to their maximal expression level (RPKM) among 7 donors. Median values from left to right: **A**: 0.29, 0.19; 0.29, 0.16; 0.26, 0.14; 0.18, 0.13; 0.24, 0.12; **B**: 0.25, 0.14; 0.27, 0.13; 0.24, 0.13; 0.17, 0.13; 0.37, 0.13. **C.** Normalized standard deviation of *de novo* granulocyte lncRNA (green) and mRNA (blue) transcripts – one transcript per locus was picked for the analysis (using *!duplicated* function in R on locus name). Median values from left to right: 0.29, 0.21, 0.29, 0.18, 0.25, 0.15, 0.27, 0.13, 0.22, 0.12.

Remarks to the boxplots: *** - $p<10^{-10}$, ** - $p<10^{-5}$, * - p<0.01, n.s. – p>0.01. The box plots display the full population but p-values are calculated using Mann–Whitney U test with equalized sample size (Methods). Numbers on the right show number of transcripts/loci in each box of the boxplots. Number in brackets (in **A**) indicate the number of loci the transcripts in each box initiate from. Outliers are not displayed in the box plots. Chr. X and Y were discarded from the variability analysis.

Figure S18

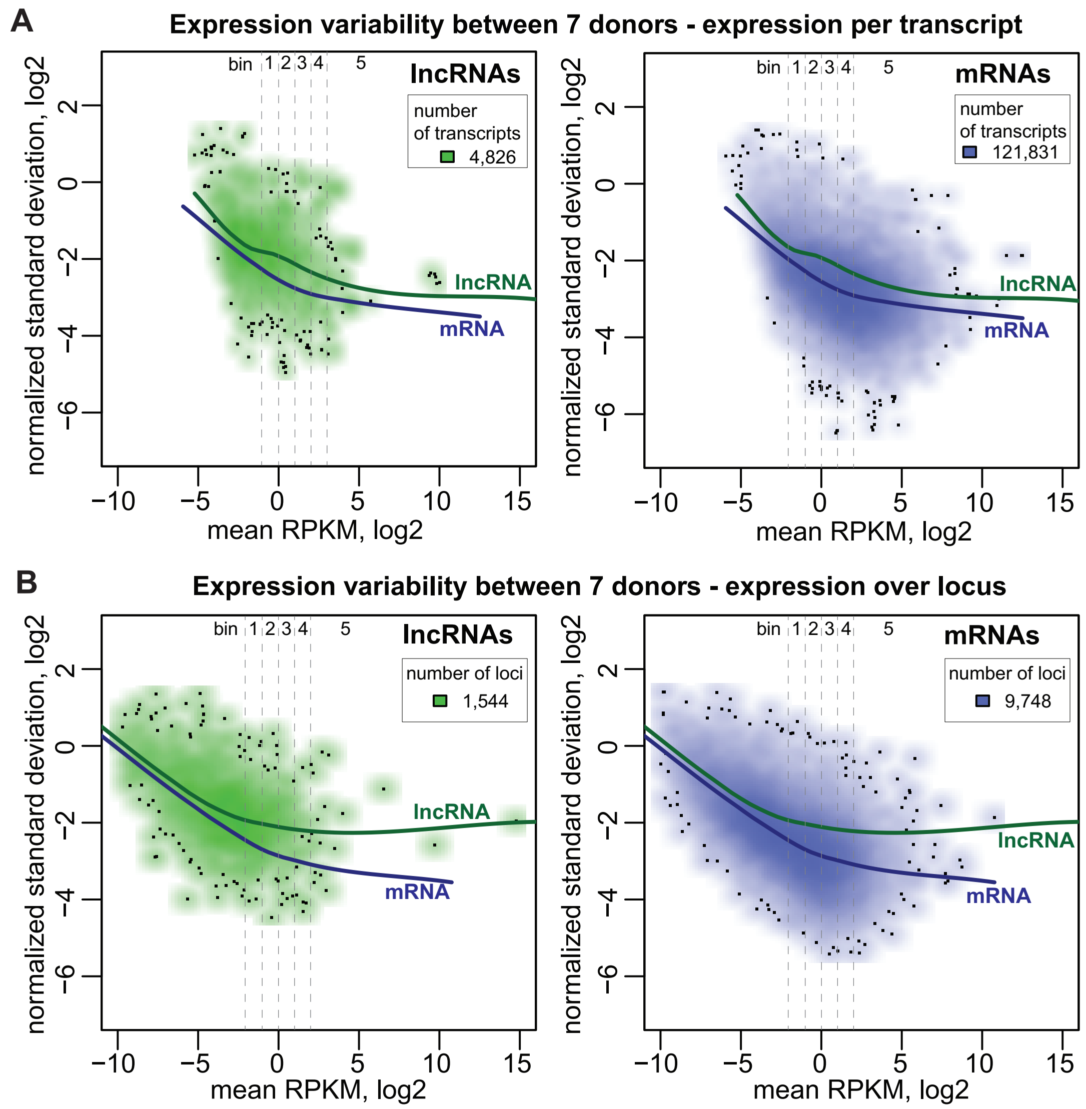

Figure S18. Plotting variability against mean RPKM level confirms increased lncRNA expression variability compared to mRNAs independent of expression level.

A. and **B.** Normalized standard deviation of *de novo* granulocyte lncRNA (green) and mRNA (blue) transcripts (**A**) and loci (**B**) expression between granulocytes from 7 donors plotted against mean expression level (RPKM) among the 7 donors. Scatter plots were built using *smoothScatter* function in R. Fitted curves were built using *loess.smooth* function in R. Both lncRNA (dark green) and mRNA (dark blue) fitted curves are displayed on each scatter plot for facilitating comparison. Dashed lines indicate the expression bins used in Figure S17. Logged (log2) values are plotted. Chr. X and Y were discarded from the variability analysis.

Figure S19

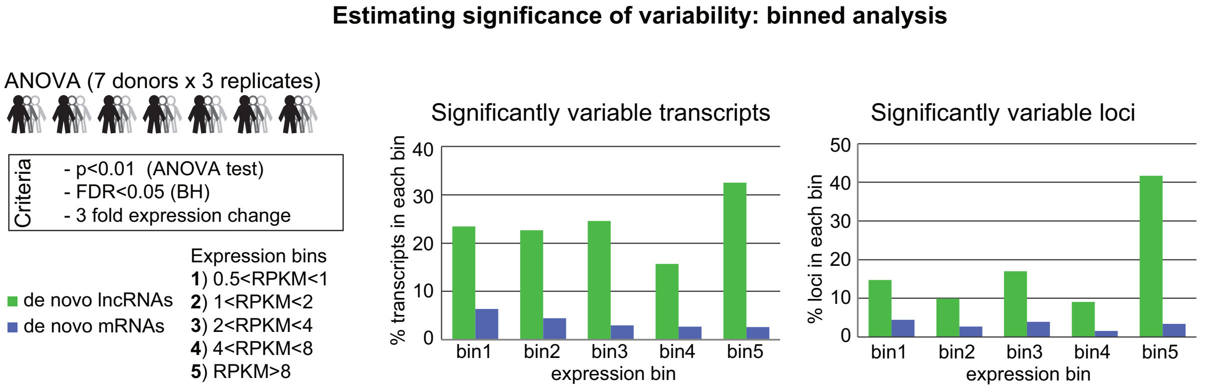

Figure S19. Percentage of *de novo* granulocyte lncRNA transcripts/loci significantly variable among seven donors is higher than that of mRNAs in all expression bins

Significance of expression variability by ANOVA test of expression variability of lncRNA (green) and mRNA (blue) transcripts (middle bar plot), and loci (right bar plot) in granulocytes from 7 donors (the 3 time points are used as replicates). Criteria for calling a transcript/locus "significantly variable": ANOVA test p value <0.01, FDR (Benjamini-Hochberg correction) <0.05, fold change between highest and lowest expression in 7 donors >3. Bar plots show percentage of significantly variable transcripts within each expression bin. LncRNA/mRNA transcripts per bin: bin1:1,064/14,942, bin2:806/18,500, bin3:553/21,179, bin4:274/19,902, bin5:292/32,367. LncRNA/mRNA locus per bin: bin1:149/1,302, bin2:101/1,195, bin3:53/824, bin4:11/501, bin5:12;/292 Chromosomes X and Y were discarded.

Figure S20

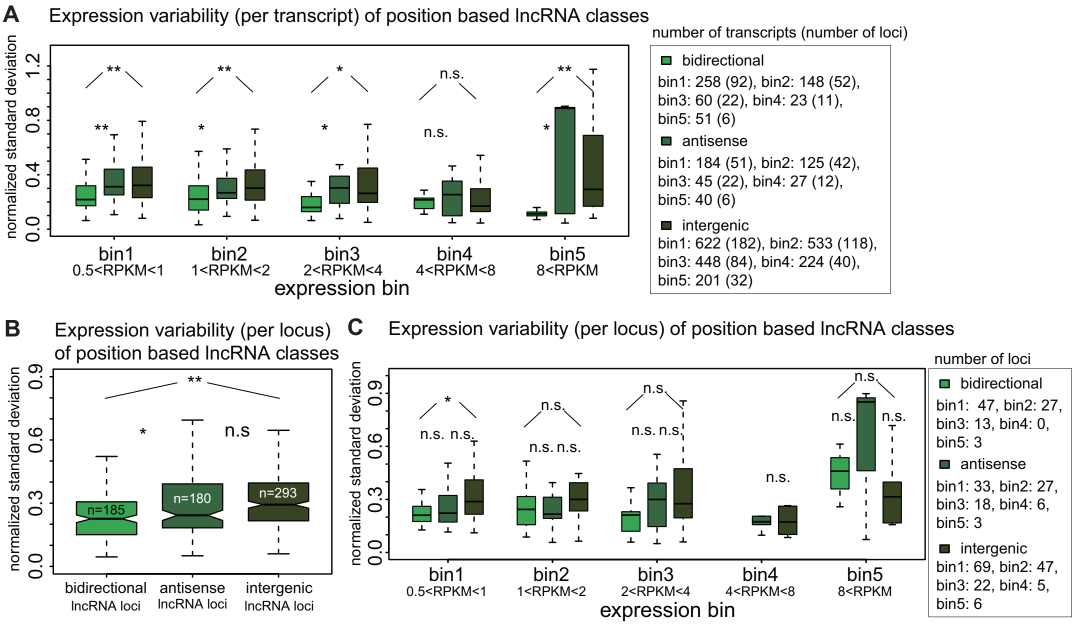

Figure S20. Bidirectional lncRNAs show reduced variability: controls

The boxplots show expression variability (normalized standard deviation) of 3 position-based classes of *de novo* annotated granulocyte lncRNAs (shades of green) between granulocyte samples from 7 donors.

A. Reduced variability of bidirectional lncRNA transcripts is persistent in all expression bins. Shown is expression variability for bidirectional, antisense and intergenic lncRNA transcripts. Median values from left to right: 0.22, 0.31, 0.32; 0.22, 0.27, 0.30; 0.16, 0.30, 0.26; 0.21, 0.25, 0.17; 0.11, 0.89, 0.29. Numbers on the right show number of transcripts in each box of the boxplot. Number in brackets indicate the number of loci the transcripts in each box initiate from. The lack of significance in bin 4 most likely arises from the low number of bidirectional and antisense transcripts in this bin.

B. Expression variability analysis over loci confirms reduced variability of bidirectional lncRNAs compared to antisense and intergenic lncRNAs. Shown is expression variability of bidirectional, antisense and intergenic lncRNA loci (shades of green). Median values from left to right: 0.23, 0.24, 0.29. The difference between antisense and bidirectional lncRNA variability is reduced compared to analysis per transcript (Fig. 4F) likely because of the bias of antisense lncRNA locus expression calculation to the bias of its highly expressed antisense protein-coding pair. This bias is not present when calculating transcript expression over exons.

C. Binned analysis of expression variability per locus does not give a meaningful confirmation for (**B**) caused by very low numbers of loci in each bin. Shown is expression variability of variability of bidirectional, antisense and intergenic lncRNA loci (shades of green). Median values from left to right: 0.21, 0.22, 0.29, 0.24, 0.22, 0.30, 0.21, 0.30, 0.28, -, 0.17, 0.17, 0.46, 0.85. 0.31. Numbers on the right show number of loci in each box of the boxplot. Absent bidirectional box in bin 4 means there were no bidirectional loci in this expression bin.

Remarks: Transcripts/loci were split into 5 bins according to their maximal expression of among 7 donors. chr X and Y were discarded from the analysis. *** - $p<10^{-10}$, ** - $p<10^{-5}$, * - p<0.01, n.s. – p>0.01. The box plots display the full population but p-values are calculated using Mann–Whitney U test with equalized sample size (Methods). Outliers are not displayed in the box plot.

Figure S21

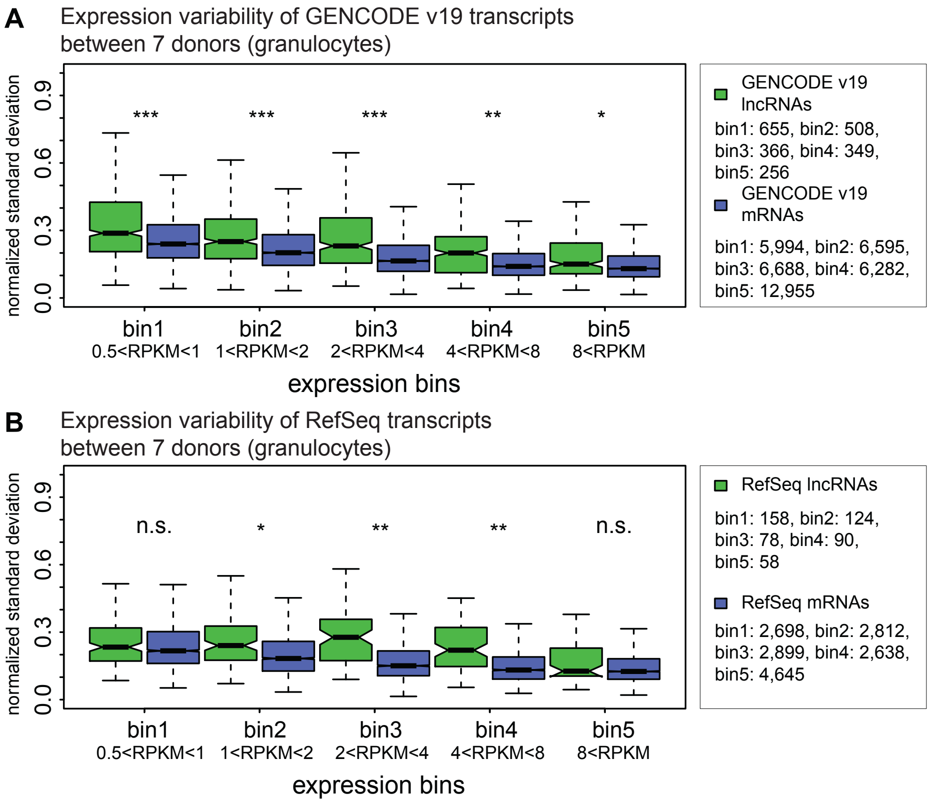

Figure S21. Increased expression variability is observed for RefSeq and GENCODE lncRNAs, however to a lesser extent

Normalized standard deviation of expression level of multi-exonic GENCODE v19 (**A**) and RefSeq (**B**) lncRNA (green) and mRNA (blue) transcripts in granulocytes between 7 donors. Transcripts are split into 5 expression bins according to their maximal expression among 7 donors. Remarks: Chr. X and Y were discarded from the analysis. *** - $p<10^{-10}$, ** - $p<10^{-5}$, * - $p<0.01$, n.s. – $p>0.01$. The box plots display the full population but p-values are calculated using Mann–Whitney U test with equalized sample size (Methods). Numbers on the right show number of transcripts in each box of the boxplot. Median values from left to right: **A**: 0.29, 0.24, 0.25, 0.20, 0.23, 0.16, 0.20, 0.14, 0.15, 0.13; **B**: 0.23, 0.22, 0.24, 0.18, 0.28, 0.15, 0.22, 0.13, 0.13, 0.13. Outliers are not displayed in the box plots.

Figure S22

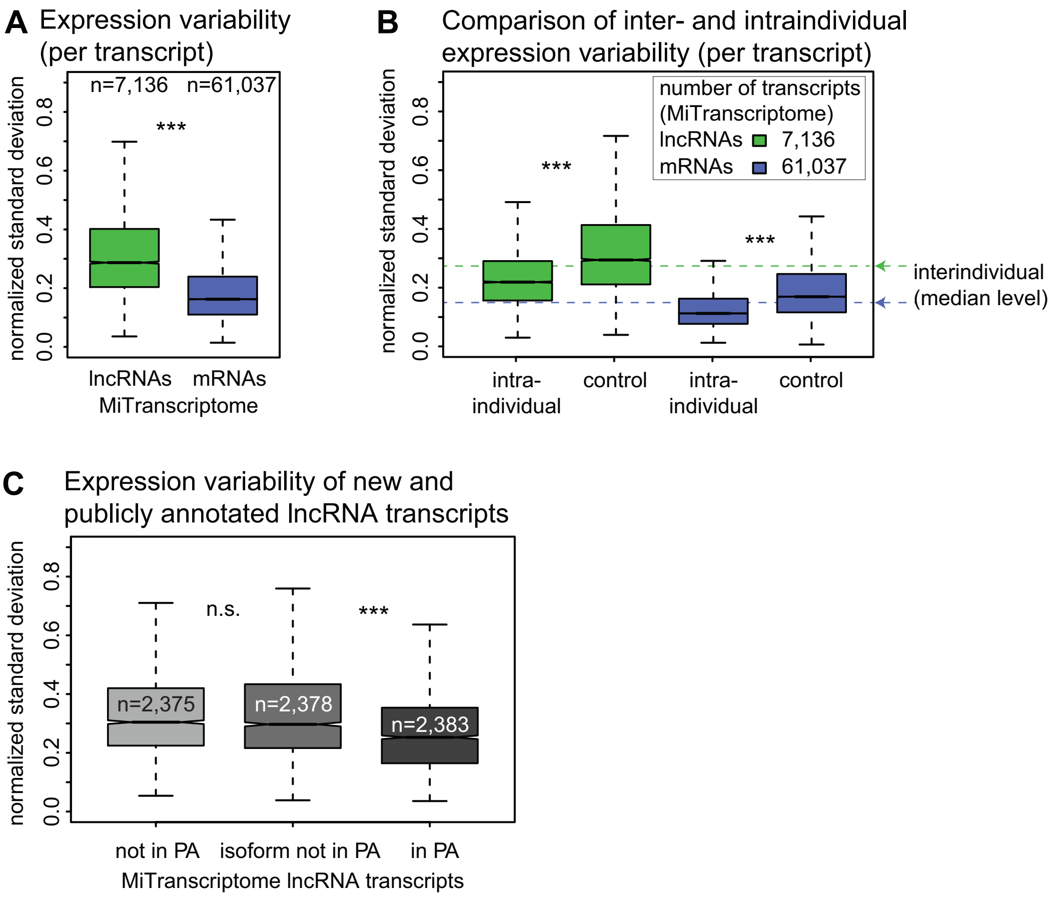

Figure S22. MiTranscriptome analysis confirms increased lncRNA expression variation

A. Genome wide inter-individual variability of multi-exonic MiTranscriptome lncRNA (green) and mRNA (blue) transcripts analyzed in the granulocyte RNA-seq data set obtained in the study. Inter-individual variability is estimated by calculating normalized (by mean) standard deviation between expression of each transcript in granulocytes from 7 donors. Expression level in each donor is averaged from three replicates. Numbers above boxes indicate number of transcripts analyzed.
B. The boxplot shows expression variability (normalized standard deviation) of MiTranscriptome lncRNA (green) and mRNA (blue) transcripts (as done for *de novo* granulocyte lncRNAs and mRNAs in Figure S16). The level of inter-individual variability between 7 donors (**A**) is indicated with green and blue dashed lines. The boxes show intra-individual variability between 3 replicates and 3 random replicates as described for Figure S16. Numbers in the legend indicate number of transcripts analyzed.
C. Novel MiTranscriptome lncRNA transcripts are more variable in our granulocyte dataset than MiTranscriptome lncRNA transcripts already present in public annotations. The boxplot shows inter-individual variability of 3 classes of multi-exonic MiTranscriptome lncRNA transcripts split according to their coverage by public annotations (Figure S14D). Numbers inside boxes indicate number of transcripts analyzed.
Remarks to boxplots: Only multi-exonic MiTranscriptome lncRNA and mRNA transcripts were analyzed. Transcripts not expressed (RPKM<0.2) in any of the 7 donors (total RNA-seq data) and transcripts from chromosomes X and Y were discarded from the analysis. The box plots display the full population but p-values are calculated using Mann–Whitney U test with equalized sample size (Methods). *** - $p<10^{-10}$, ** - $p<10^{-5}$, * - $p<0.01$, n.s. – $p>0.01$. Median values from left to right: **A**: 0.29, 0.16; **B**: 0.23, 0.31, 0.13, 018; **C**: 0.30, 0.30, 0.25. Outliers are not displayed in the boxplots.

Figure S23

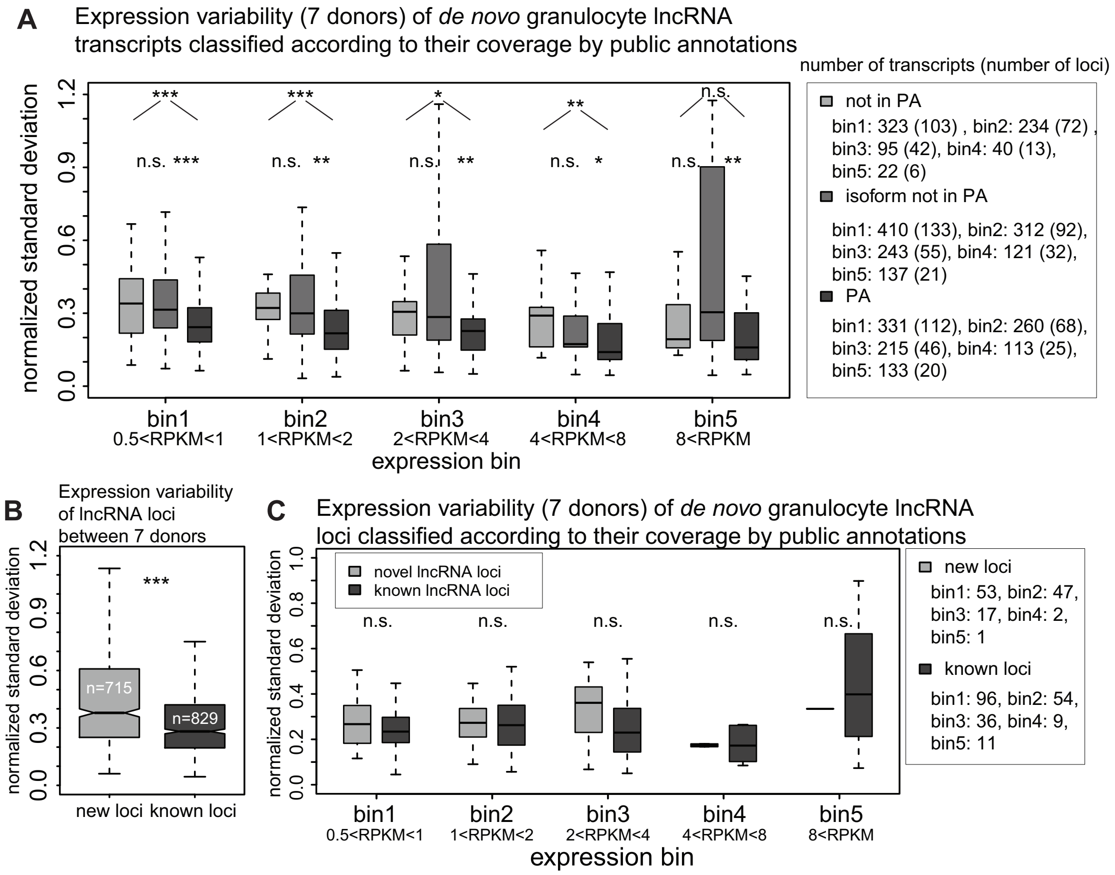

Figure S23. Granulocyte lncRNA transcripts not present public annotations (PA) show increased variability: controls

The boxplots show expression variability (normalized standard deviation) between 7 donors in granulocytes of *de novo* granulocyte lncRNA transcripts/loci classified according to their presence in public annotations.

A. Increased variability of “not in PA” and “isoform not in PA” lncRNA transcripts is persistent in all expression bins. Shown is expression variability for “not in PA” transcripts (light gray), “isoform not in PA” transcripts (medium gray) and “PA” transcripts (dark gray). Median values from left to right: 0.34, 0.31, 0.24; 0.32, 0.30, 0.22; 0.31, 0.28, 0.23; 0.29, 0.17, 0.14; 0.19, 0.30, 0.16. Numbers on the right show number of transcripts in each box of the boxplot. Number in brackets indicate the number of loci the transcripts in each box initiate from.

B. Expression variability analysis over loci confirms increased variability of lncRNAs not covered by public annotations. Shown is expression variability of “new” (light gray) and “known” loci. Median values from left to right: 0.38, 0.28.

C. Binned analysis of expression variability per locus does not give a meaningful confirmation for (**B**) caused by very low numbers of loci in each bin. Shown is expression variability of “new” (light gray) and “known” (dark grey) loci. Median values from left to right: 0.27, 0.23, 0.27, 0.26, 0.36, 0.23, 0.17, 0.17, 0.33, 0.40. Numbers on the right show number of loci in each box of the boxplot.

Remarks: Transcripts/loci were split into 5 bins according to their maximal expression of among 7 donors. chr X and Y were discarded from the analysis. *** - $p<10^{-10}$, ** - $p<10^{-5}$, * - $p<0.01$, n.s. – $p>0.01$. The box plots display the full population but p-values are calculated using Mann–Whitney U test with equalized sample size (Methods) Outliers are not displayed in the box plot.

Figure S24

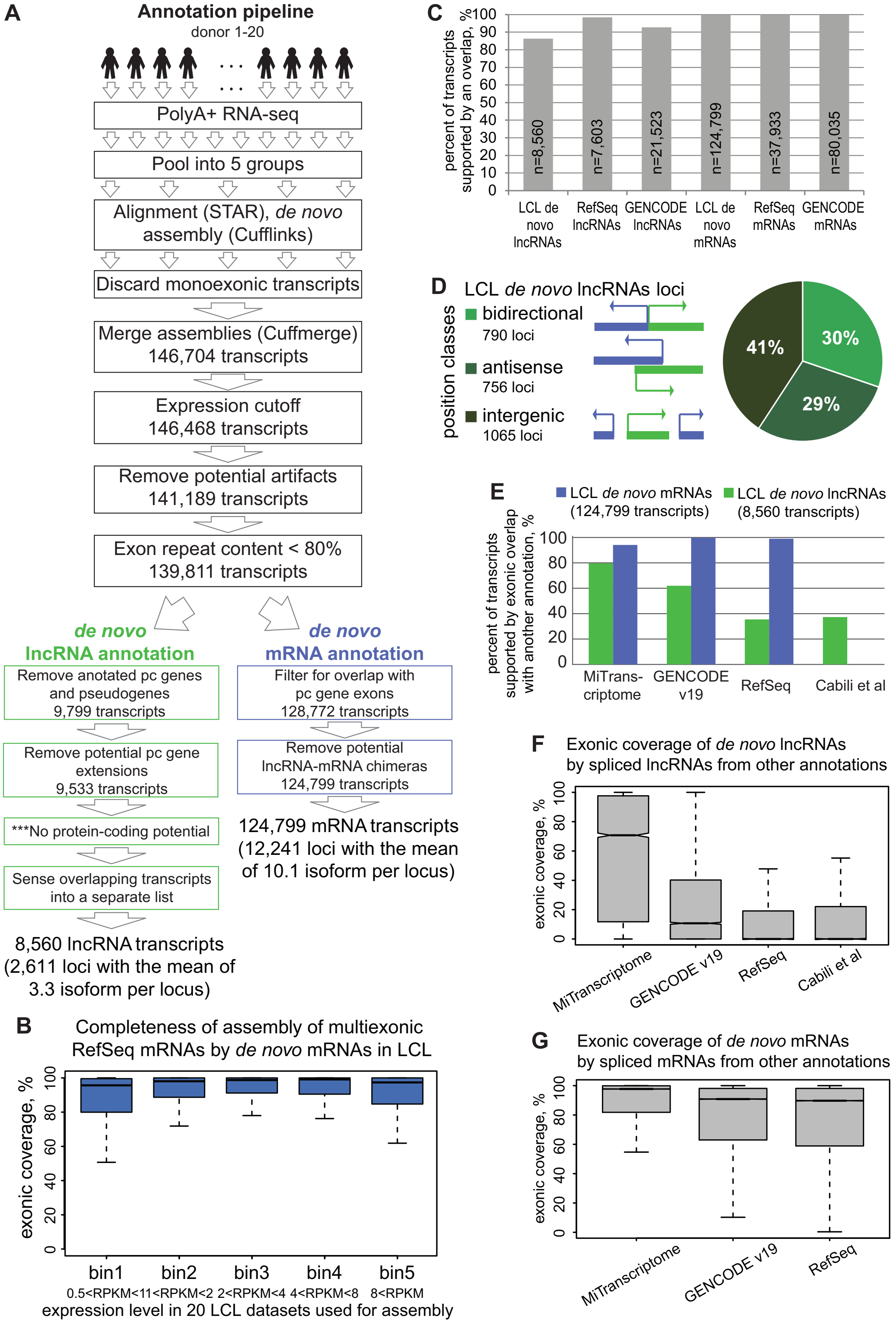

Figure S24. Defining the lncRNA transcriptome of LCL (Lymphoblastoid cell line)

A. Pipeline for *de novo* lncRNA and *de novo* mRNA identification in LCL with transcript numbers at

different stages.

B. Completeness of assembly. Exonic coverage of multi-exonic RefSeq mRNAs by *de novo* mRNA annotation in LCL. Genes are split into 5 bins according to their average expression in 20 donors LCL PolyA+ RNA-seq datasets used for the transcriptome assembly in order to account for the bias between expression level and assembly success. Median values from left to right: 95.7, 98.0, 98.8, 99.1, 97.4.

C. The majority of *de novo* lncRNAs in LCL are supported by an overlap with an EST. The bar plot shows the percentage of transcripts that have a sense exonic overlap with a spliced EST (human EST database downloaded from UCSC) for (from left to right): *de novo* lncRNAs, annotated in this study in LCL, lncRNAs annotated by RefSeq and GENCODE v19, *de novo* mRNAs, annotated in this study in LCL, mRNAs annotated by RefSeq and GENCODE v19. Numbers inside bars indicate total number of transcripts in each annotation.

D. Distribution of the *de novo* annotated lncRNA loci in LCL according to their position relative to protein-coding genes (*de novo*, GENCODE v19 and RefSeq mRNAs). The pie chart shows that 30% (790) of the lncRNA loci are bidirectional (light green), 29% (756) are antisense (medium green) and 41% (1,065) are intergenic (dark green) relative to protein-coding genes.

E. The majority of *de novo* lncRNAs in LCL are supported by an overlap with MiTranscriptome lncRNAs. The bar plot shows percentage of LCL *de novo* lncRNA (green) and mRNA (blue) transcripts supported by an exonic overlap with a multi-exonic lncRNA or mRNA respectively from MiTranscriptome, GENCODE v19, RefSeq and Cabili et al (only lncRNAs) annotations.

F. MiTranscriptome provides best exonic coverage for LCL *de novo* lncRNAs. The box plot shows percent exonic coverage of LCL *de novo* lncRNAs by multi-exonic lncRNAs from MiTranscriptome, GENCODE v19, RefSeq and Cabili et al annotations [2-5]. Number of LCL *de novo* lncRNA transcripts examined in each box – 8,560. Median values from left to right: 70.7, 10.7, 0, 0.Outliers are not displayed in the boxplot.

G. LCL *de novo* mRNAs are nearly fully exonically covered by MiTranscriptome and public annotations. The box plot shows percent exonic coverage of LCL *de novo* mRNAs by multi-exonic mRNAs from MiTranscriptome, GENCODE v19 and RefSeq annotations. Number of LCL *de novo* mRNA transcripts examined in each box – 124,799. Median values from left to right: 97.7, 90.9, 89.7. Outliers are not displayed in the boxplot.

Figure S25

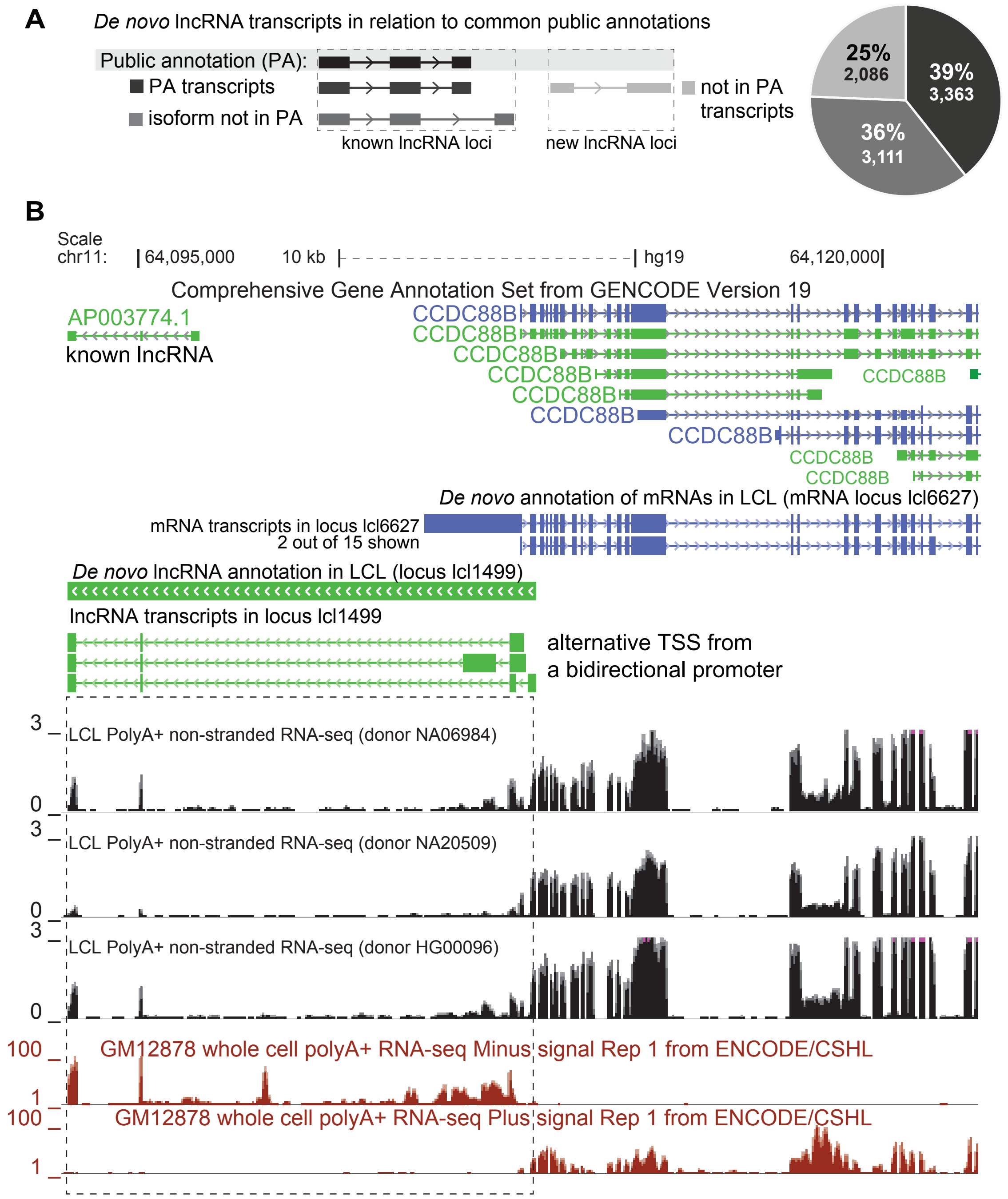

Figure S25. Identification of novel lncRNA transcripts and novel isoforms of known lncRNA loci in LCL cells

A. Distribution of 8,560 *de novo* LCL lncRNA transcripts annotated in the study according to their coverage by the 3 commonly used public annotations as described in Fig. 2A. Publicly annotated ("PA") transcripts constitute 39% of all LCL lncRNA transcripts (3,363 transcripts, black), "isoform not in PA" - 36% (3,111 transcripts, dark grey), "not in PA" – 25% (1,921 transcripts, light grey).

B. An example of a known lncRNA locus (AP003774.1 annotated by GENCODE v19) on chromosome 11 which was extended by our *de novo* annotation in LCL (locus lcl1499) with three new isoforms with an alternative TSS. From top to bottom: GENCODE v19 annotates a lncRNA AP003774.1 and a protein coding gene CCDC88B in this region; *de novo* mRNA annotation in LCL; *de novo* lncRNA annotation in LCL: lncRNA locus 1499 is formed by 3 novel lncRNA isoforms

bidirectional to the CCDC88B protein coding gene, the isoforms share two last exons with the GENCODE annotated lncRNA; Normalized non-strand-specific LCL PolyA+ RNA-seq signal: donor NA06984 - high expression of the lncRNA (average RPKM per transcript 5.8); donor NA20509 - lower expression (average RPKM per transcript 2.6); donor HG00096 - high expression (average RPKM per transcript 6.1); strand-specific PolyA+ RNA-seq signal for LCL sample from ENCODE (GM12878, RNA-sequencing track displayed from ENCODE RNA-seq public hub in UCSC browser) showing the expression of the extended lncRNA locus from reverse strand (Minus Signal) and expression of the protein coding gene from the forward strand (Plus Signal) (average RPKM of the three lncRNA transcripts - 2.9). Dashed box over RNA-seq signal outlines the area of lncRNA expression.

Figure S26

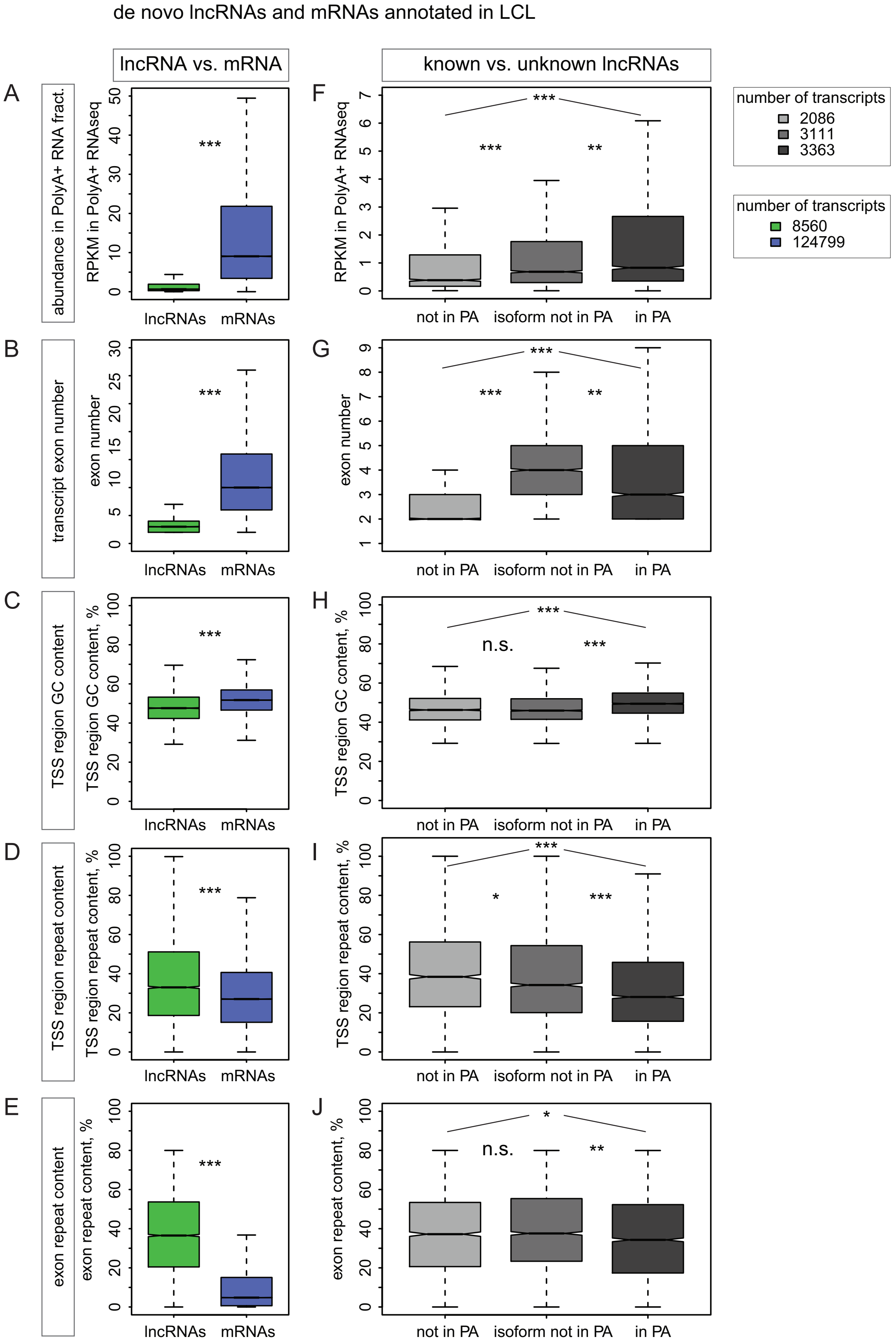

Figure S26. Analyzing features of LCL lncRNAs confirms that difficult to identify lncRNAs are more different from mRNAs than publicly annotated lncRNAs

A. The abundance in the PolyA enriched RNA fraction of LCL *de novo* lncRNA and mRNA transcripts calculated as average from all 462 available PolyA+ RNA-seq samples. Exon number (**B**), percent GC content of TSS region (TSS +/- 1.5 kb) (**C**), percent repeat coverage of TSS region (**D**) and exons (**E**) of *de novo* annotated lncRNAs and mRNA transcripts. Abundance in PolyA enriched RNA fraction (**F**), exon number (**G**), percent GC content of TSS region (**H**), percent repeat coverage of TSS region (**I**) and exons (**J**) of the three classes of *de novo* lncRNA as described in Figure S25A. Remarks: green - all *de novo* lncRNAs, blue – all *de novo* mRNAs, light gray – "not in PA" lncRNA transcripts, medium gray – "isoform not in PA" lncRNA transcripts, dark gray – "PA" lncRNA transcripts. Transcript numbers in each box are indicated on the right. *** - $p<10^{-10}$, ** - $p<10^{-5}$, * - $p<0.01$, n.s. – $p>0.01$. The box plots display the full population but p-values are calculated using Mann–Whitney U test with equalized sample size (Methods). Median values from left to right: **A**: 0.66, 9.05; **B**: 3, 10; **C**: 47.6, 51.7; **D**: 33.0, 27.0; **E**: 36.5, 4.8; **F**: 0.38, 0.68, 0.83; **G**: 2, 4, 3; **H**: 46.3, 45.9, 49.4; **I**: 38.4, 34.2, 28.1; **J**: 37.2, 37.6, 34.3. Outliers are not displayed in the boxplot.

Figure S27

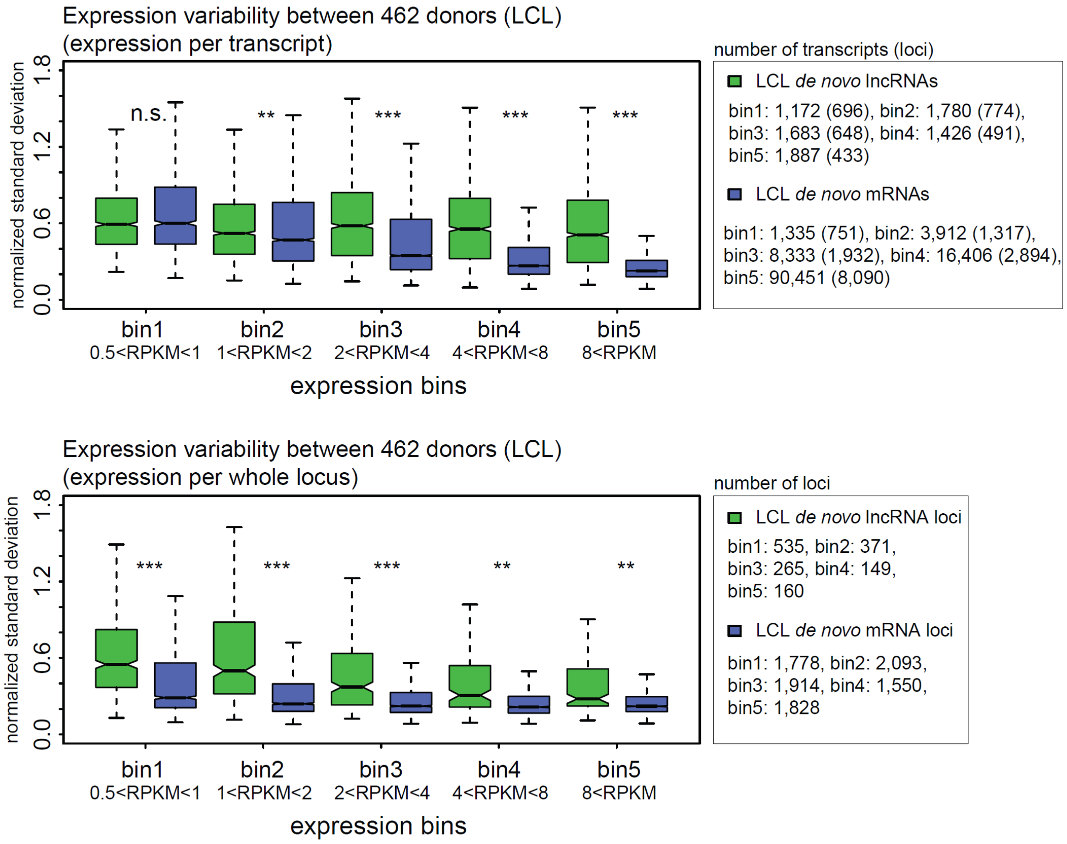

Figure S27. Higher LCL *de novo* lncRNA expression variability is not caused by lower lncRNA expression level.

The phenomenon of increased expression variability of lncRNAs compared to mRNAs is not biased to the absolute expression level of two types of transcripts in the LCL dataset. Normalized standard deviation of *de novo* LCL lncRNA (green) and mRNA (blue) transcripts **(top)** or loci **(bottom)** expression in LCL from 462 donors split into 5 expression bins according to their maximal expression level (RPKM) among 462 donors. Note, that in bin 1 of the upper box plot the difference between lncRNAs and mRNAs is not significant, in contrast to similar analysis performed in granulocytes (Figure S17A). This is likely caused by the increased number of donors (462 - LCL vs. 7 - granulocytes) and the way we split transcripts/loci into bins by their maximal expression among all donors. Thus, bin 1 most likely represents not expressed or marginally expressed transcripts, with an outlier reaching the maximal RPKM of 0.5 to 1, whose variability is strongly affected by detection bias. Such technical bias affects any transcript equally and thus lncRNA and mRNA expression variability is indistinguishable in bin 1. Note that variability of expression calculated over the whole locus (bottom plot) shows consistent lncRNA/mRNA difference in all bins. Remarks: Chr. X and Y were discarded from the analysis. *** - $p<10^{-10}$, ** - $p<10^{-10}$, * - $p<0.01$, n.s. – $p>0.01$. The box plots display the full population but p-values are calculated using Mann–Whitney U test with equalized sample size (Methods). Numbers on the right show number of transcripts/loci in each box of the boxplots. Number in brackets (top boxplot) indicates the number of loci the transcripts in each box initiate from. Median values from left to right: top plot: 0.59, 0.60, 0.51, 0.47, 0.58, 0.35, 0.55, 0.27, 0.51, 0.23; bottom plot: 0.55, 0.29, 0.50, 0.24, 0.37, 0.22, 0.31, 0.21, 0.28, 0.22.Outliers are not displayed in the boxplot.

Figure S28

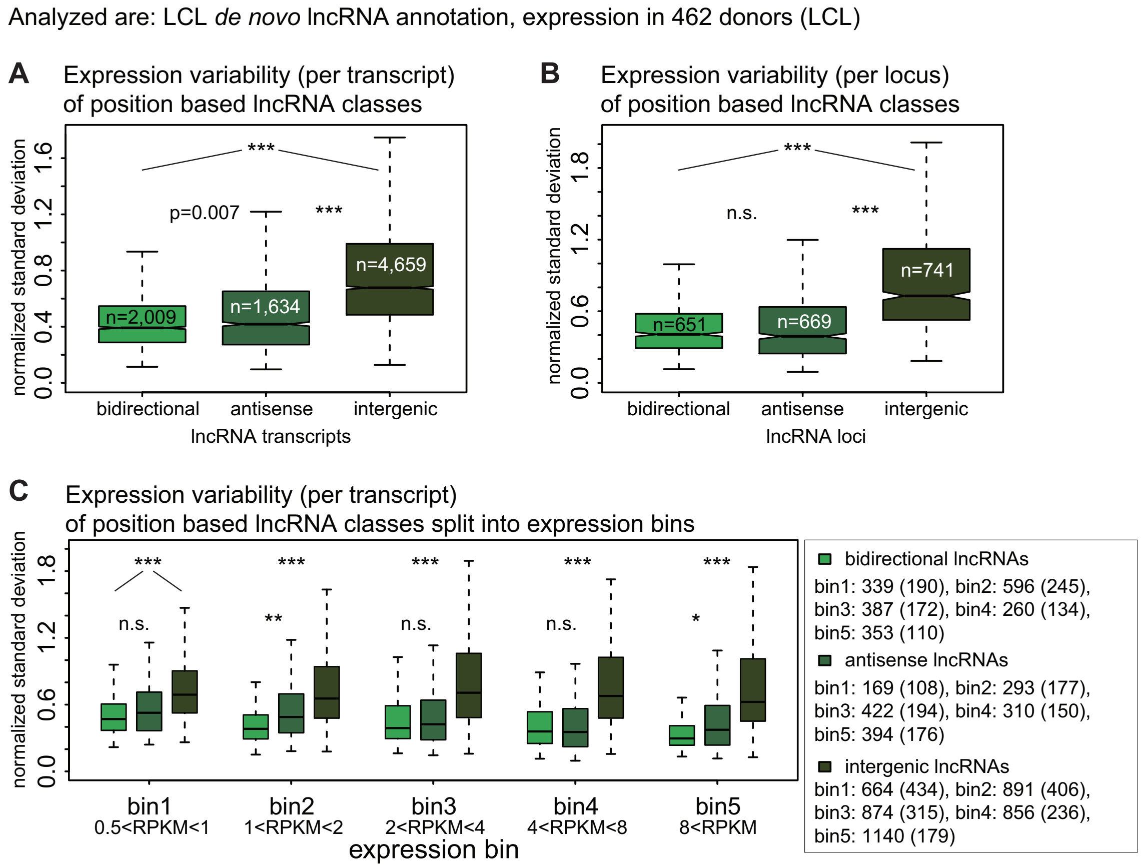

Figure S28. Bidirectional lncRNAs annotated in LCL show reduced variability

A. and **B.** Bidirectional *de novo* lncRNAs annotated in LCL show a decreased level of expression variability. The boxplot shows inter-individual variability of 3 classes of lncRNA transcripts (A)/ loci (B) split according to their position relative to protein coding genes (Figure S24D, Methods). Numbers inside the boxes on the right indicate number of transcripts/loci in each box.

C. Bidirectional LCL lncRNA transcripts are less variable than intergenic lncRNAs independently of expression bin. The boxplot shows inter-individual variability of 3 classes of lncRNAs. Transcripts were split into 5 bins according to their maximal expression among 462 donors. Numbers on the right show number of transcripts in each box of the boxplot. Numbers in brackets indicate the number of loci the transcripts in each box initiate from.

Remarks: Inter-individual variability is calculated as normalized standard deviation of transcripts expression in LCL samples from 462 donors. Remarks: Chr. X and Y were discarded from the analysis. *** - $p<10^{-10}$, ** - $p<10^{-5}$, * - $p<0.01$, n.s. – $p>0.01$. The box plots display the full population but p-values are calculated using Mann–Whitney U test with equalized sample size (Methods). Median values from left to right: **A**: 0.39, 0.42, 0.68; **B**: 0.41, 0.39, 0.73; **C**: 0.47, 0.53, 0.69; 0.38, 0.49, 0.65; 0.39, 0.42, 0.71; 0.36, 0.35, 0.68; 0.30, 0.37, 0.62. Outliers are not displayed in the boxplot. Antisense lncRNAs expression analysis in the LCL dataset (particularly that of expression over the whole locus (**B**)) could be biased to the expression of the overlapped mRNAs since the RNA-seq data was not strand-specific and thus expression variability of antisense lncRNAs is reduced accordant to the reduced mRNA expression variability (Fig. 5D).

Figure S29

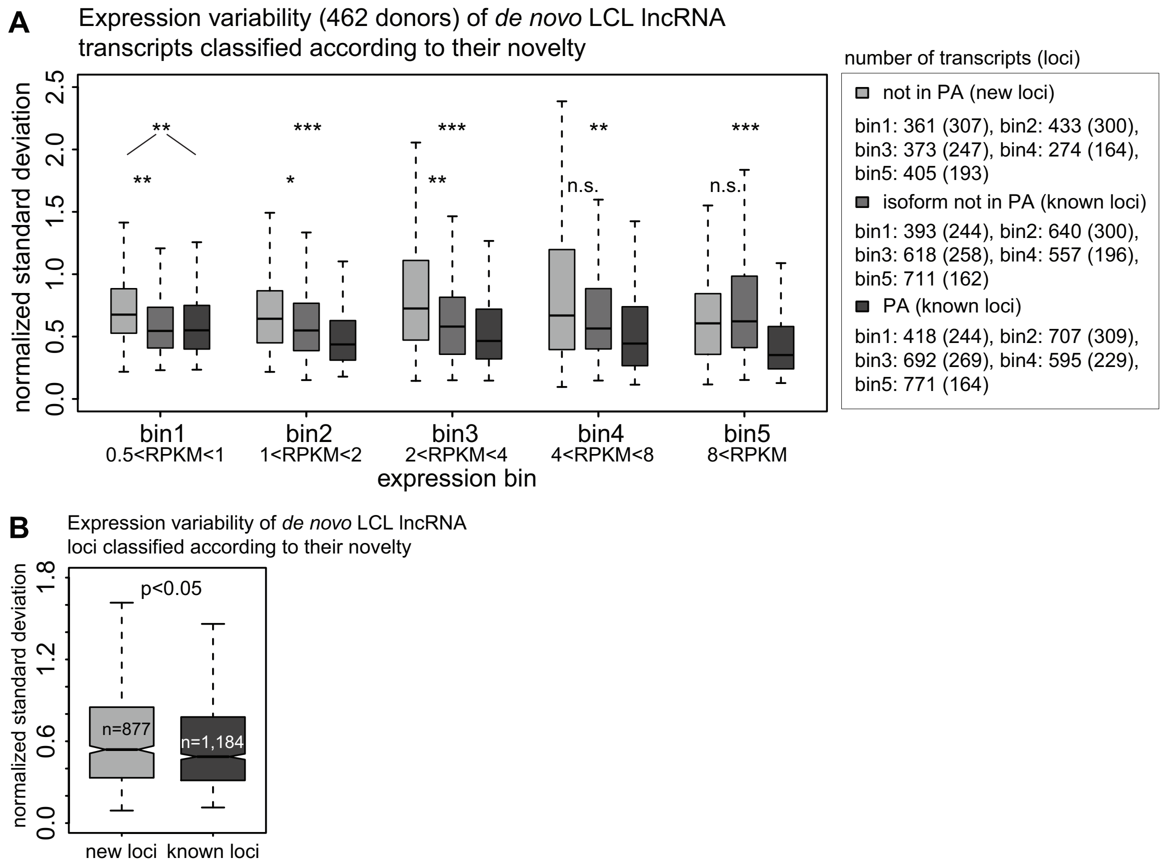

Figure S29. New lncRNAs are more variable than known lncRNAs from LCL de novo annotation: controls

A. Increased variability of "not in PA" and "isoform not in PA" lncRNA transcripts (Figure S25A in Additional File 1 and Fig. 5F) is persistent in all expression bins. The boxplot shows expression variability (normalized standard deviation) of "not in PA" LCL *de novo* lncRNA transcripts (light gray), "isoform not in PA" LCL *de novo* lncRNA transcripts (medium gray) and "PA" LCL *de novo* lncRNA transcripts (dark gray) between LCL samples from 462 donors. Transcripts were split into 5 bins according to their maximal expression of among 462 donors. Median values from left to right: 0.68, 0.55, 0.55; 0.64, 0.55, 0.44; 0.73, 0.58, 0.46; 0.67, 0.56, 0.44; 0.61, 0.62, 0.35. Numbers on the right show number of transcripts in each box of the boxplot. Numbers in brackets indicate the number of loci the transcripts in each box initiate from.

B. Variability of de novo LCL lncRNAs not present in public annotations is slightly increased over annotated lncRNAs when performing "per locus" analysis. The boxplot shows expression variability (normalized standard deviation) of "new" (light gray) and "known" (dark gray) lncRNA loci between LCL samples from 462 donors. Median values from left to right: 0.54, 0.49.

Remarks: chr X and Y were discarded from the analysis. *** - $p<10^{-10}$, ** - $p<10^{-5}$, * - p<0.01, n.s. – p>0.01. The box plots display the full population but p-values are calculated using Mann–Whitney U test with equalized sample size (Methods). Outliers are not displayed in the boxplot.

Figure S30

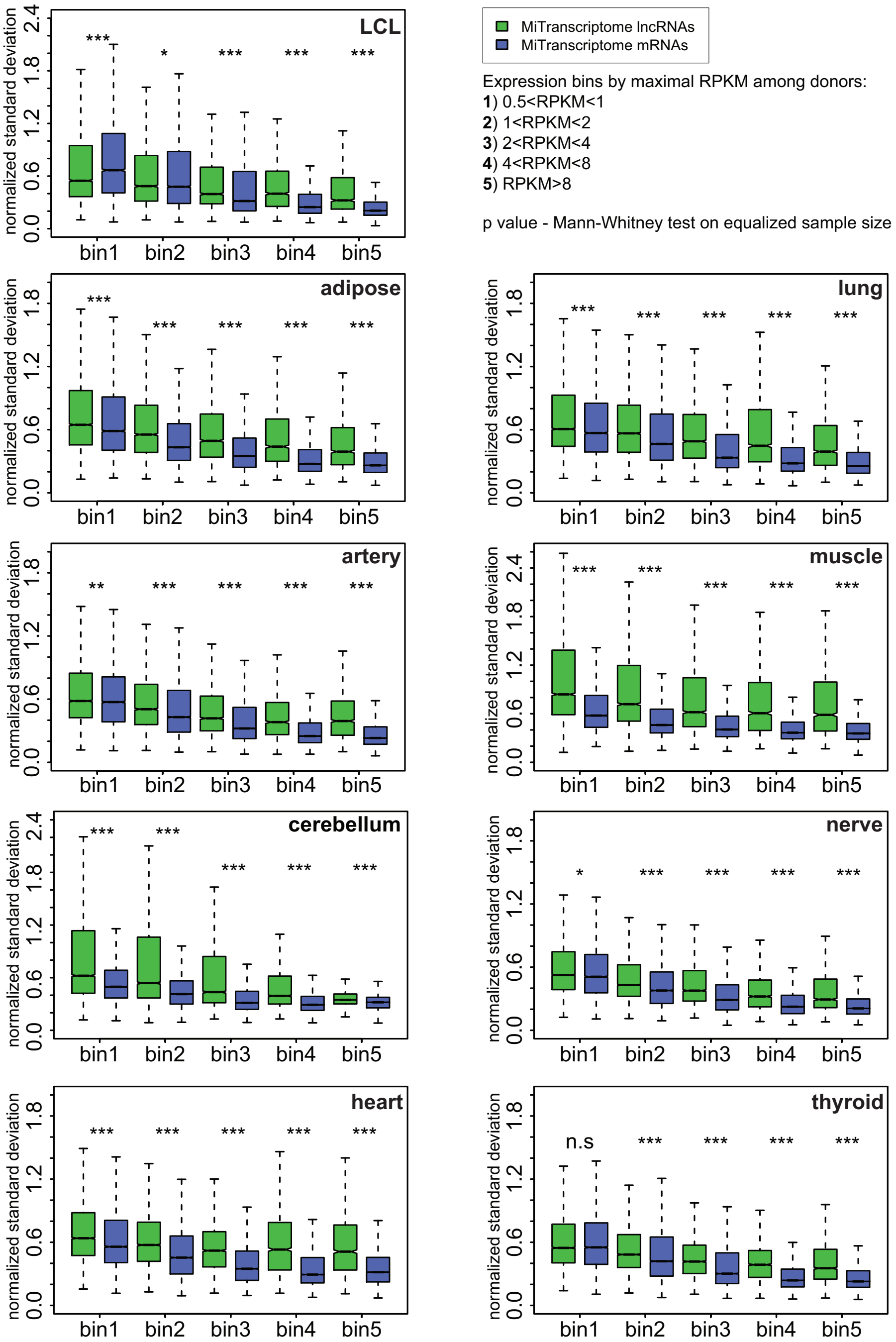

Figure S30. Confirmation of increased lncRNA expression variability in multiple human tissues using GTEx project data: expression level control

Binned normalized standard deviation of MiTranscriptome lncRNA (green) and mRNA (blue) transcripts expression between 20 donors in 9 tissues (as described for Fig. 6, Results, Methods). Transcripts were split into 5 expression bins according to their maximal expression level (RPKM) among 20 donors for each tissue. Chromosomes X and Y were discarded from the analysis. Remarks to the boxplots: *** - $p<10^{-16}$, ** - $p<10^{-10}$, * - p<0.01, n.s. – p>0.01. The box plots display the full population but p-values are calculated using Mann–Whitney U test with equalized sample size (Methods). Outliers are not displayed in the box plots. Tissue name is indicated for each box plot (top right). Median values from left to right: LCL: 0.54, 0.67, 0.49, 0.48, 0.40, 0.32, 0.40, 0.25, 0.32, 0.21 ; adipose: 0.54, 0.55, 0.48, 0.42, 0.42, 0.30, 0.39, 0.24, 0.35, 0.23 ; artery: 0.58, 0.57, 0.50, 0.43, 0.42, 0.32, 0.38, 0.25, 0.39, 0.23; cerebellum: 0.62, 0.50, 0.54, 0.41, 0.44, 0.31, 0.39, 0.29, 0.35, 0.32 ; heart: 0.64, 0.56, 0.57, 0.45, 0.52, 0.35, 0.53, 0.29, 0.51, 0.32; lung: 0.61, 0.57, 0.57, 0.46, 0.49, 0.33, 0.45, 0.28, 0.39, 0.26; muscle: 0.84, 0.58, 0.72, 0.46, 0.62, 0.41, 0.61, 0.37, 0.59, 0.36; nerve: 0.53, 0.51, 0.43, 0.38, 0.38, 0.29, 0.32, 0.22, 0.29, 0.21; thyroid: 0.55, 0.55, 0.48, 0.42, 0.42, 0.30, 0.39, 0.24, 0.35, 0.23.

Figure 31

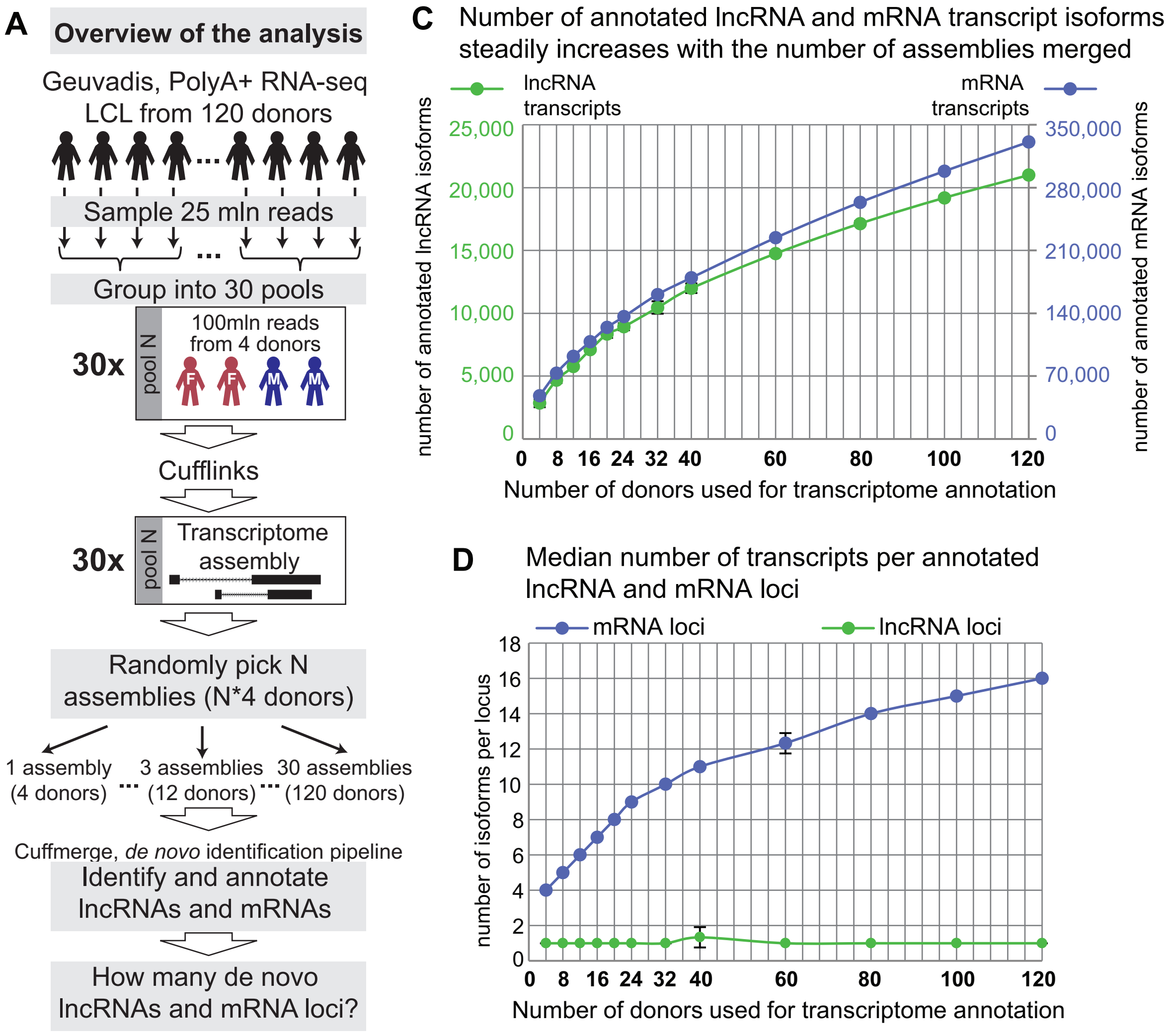

B

number of replicates of random pick	number of assemblies	number of donors
3	1	4
3	2	8
3	3	12
3	4	16
3	5	20
3	6	24
3	8	32
3	10	40
3	15	60
3	20	80
3	25	100
1	30	120

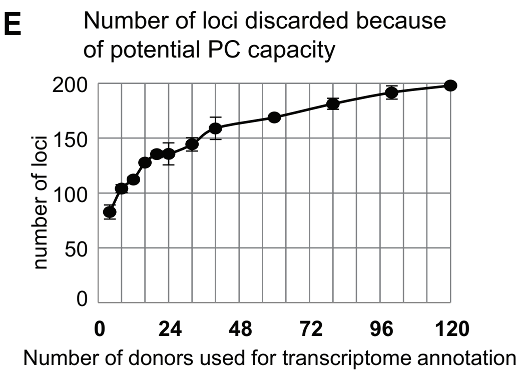

Figure S31. *De novo* identification of lncRNA and mRNA loci in LCL using variable number of donors.

A. Algorithm for investigating the relation between the number of identified lncRNA loci and the number of donors analyzed. 120 donors (out of total 462 donors available from Geuvadis dataset [2]) were picked to be used in the study. Only unrelated samples with > 25 million reads were used. Each of the five population groups was represented by 12 females and 12 males (Additional File 11A). All

the 120 RNA-seq data sets were randomly down-sampled to give 25 million paired-end reads each. 120 donors were grouped into 30 pools (each pool contained 2 females and 2 males from the same population) of 100 (25x4) million reads each. Each pool was used to assemble LCL transcriptome using Cufflinks resulting in 30 transcriptome assemblies. We then used 1, 2, 3, 4, 5, 6, 8, 10, 15, 20, 25, 30 transcriptome assemblies to *de novo* annotate lncRNAs and mRNAs in LCL using the annotation pipeline established in the study and plot the number of annotated loci vs. number of donors as an output of the analysis (see Figure 7C and Additional File 11C).

B. For each number of assemblies (i.e. each data point) we performed random picking from the list of 30 assemblies (Additional File 11B).

C. Number of transcript isoforms increases similarly for lncRNA and mRNA loci with the increase of number of donors used for transcript annotation. The plot shows number of LCL *de novo* lncRNA (green) and mRNA (blue) transcripts annotated using different number of assemblies obtained from different number of donors. The Y-axis corresponding to lncRNAs (green) is placed on the left, the Y-axis corresponding to mRNAs (blue) is placed on the right. The range of values from 0 to 25,000 transcripts for lncRNAs and 14x fold more – from 0 to 350,000 for mRNAs. In spite of differing absolute numbers the dynamics of the increase is the same for lncRNAs and mRNAs. Maximum number of lncRNA transcripts - 20,992, maximum number of mRNA transcripts - 330,811 (data table Additional File 11C). Error bars that represent standard deviation in transcript number between three replicates of random assembly picking (**B**) are present for all data points but mostly not visible due to their low values.

D. Increasing donor numbers allows identification of an increasing number of isoforms per mRNA locus, whereas lncRNAs keep low median number of transcripts per locus while increasing the number of loci annotated in the genome. The plot shows the median transcript number in LCL *de novo* lncRNA (green) and mRNA (blue) loci annotated using different number of assemblies obtained from different number of donors. Error bars represent the standard deviation of loci number between 3 replicates of random picking for each number of assemblies used for identification (data table Additional File 11C). Error bars that represent standard deviation between three replicates are present for all data points but mostly not visible due to their low values.

E. Estimating how many unknown mRNA loci could be assembled and then discarded at the protein-coding capacity filtering step with the increasing number of donors.

Figure S32

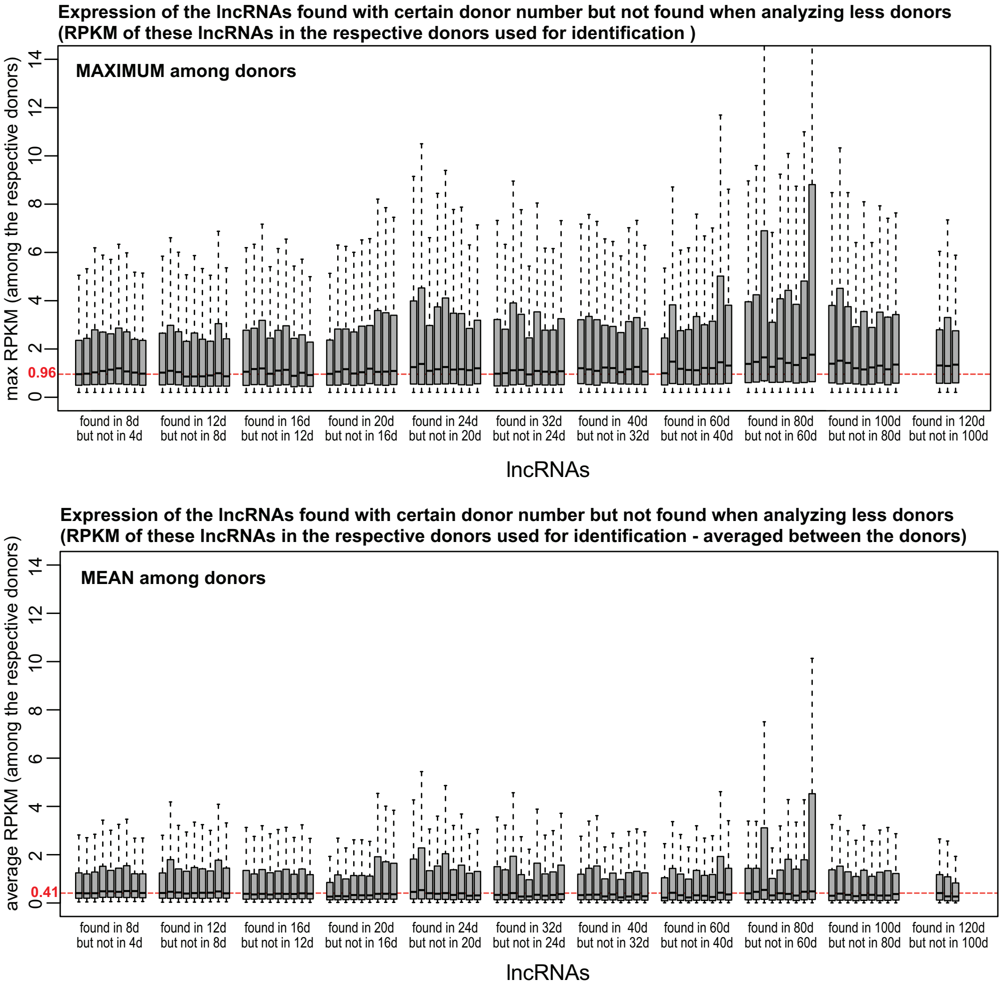

Figure S32. Increasing donor number does not tend to identify only marginally expressed lncRNAs

Overview: Expression level of the identified lncRNAs (Fig. 7C) is not decreasing with the increase of donor number and potential sensitivity of the pipeline. We asked if the lncRNA loci that we identified by adding more donors to the analysis were more lowly expressed than those identified using less donors. If so, this would indicate that the amount of sequencing data rather than number of individuals allows identification of new minimally expressed lncRNA loci. We plotted the expression level of lncRNA transcripts initiating from loci annotated using more donors, that could not be identified (defined as 50% sense overlap) using less donors. We found that expression of lncRNAs that require more and more donors to be identified does not anti-correlate with the donor number. Thus the identification of more lncRNAs in larger donor collections does not specifically identify lowly expressed transcripts.

The boxplot shows the maximal (**top**) and mean (**bottom**) (among the donors used for the annotation) expression level of *de novo* LCL lncRNA transcripts annotated using the indicated number of donors (indicated on the X axis). Only transcripts expressed from loci that have not been identified ("identified"= >50% sense overlap) using less donors (indicated on the X axis) are displayed. 9 boxes for each number of donors show the result for all the nine possible pairwise comparisons between three replicates of each donor-number annotations (e.g. "found in 8d but not in 4d": box1. lncRNA loci in 8-donor annotation replicate 1 not identified in 4-donor annotation replicate 1, box2. lncRNA loci in 8-donor annotation replicate 1 not identified in 4-donor annotation replicate 2, box3. lncRNA loci in 8-donor annotation replicate 1 not identified in 4-donor annotation replicate 3, box4. lncRNA loci in 8-donor annotation replicate 2 not identified in 4-donor annotation replicate 1, etc). 120-donor annotation only has 1 replicate, thus giving just three boxes. Outliers are not displayed in the boxplot. Horizontal dashed red line and red number indicate median level of the first box in the boxplot.

Figure S33

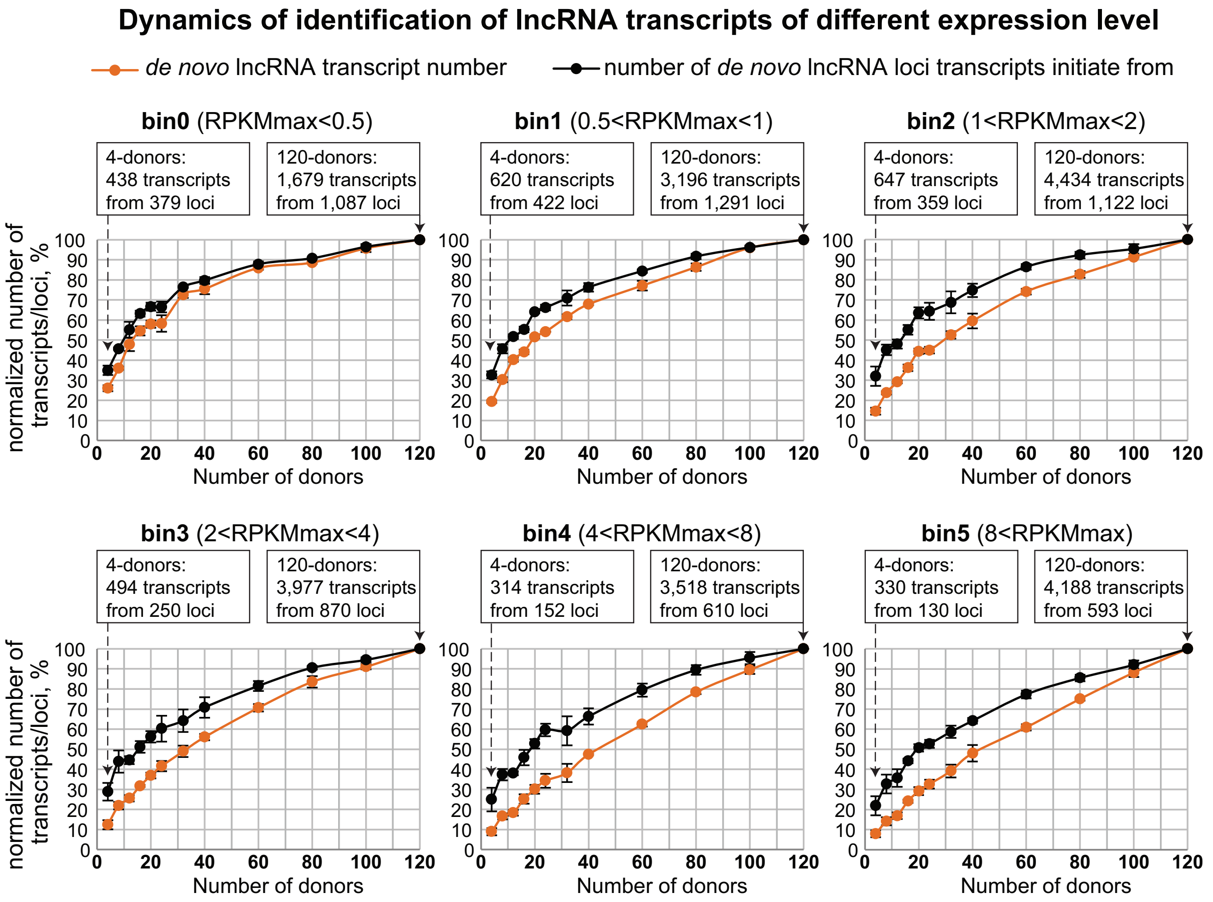

Figure S33. Increasing donor number identifies increased numbers lncRNAs in all expression bins.

Dynamics of identification upon donor number increase of transcripts split into 6 bins according to their maximal expression among donors used for their identification (Additional File 11E). Every plot shows number of transcripts (orange) and loci (black) these transcripts initiate from. Number of transcripts/loci is normalized to the number of transcripts/loci in 120-donor annotation. Absolute number of transcript/loci is given for 4-donor and 120-donor annotations (boxes above the plots). Error bars show standard deviation between 3 replicates for each donor number. Remarks: bin 0 has not been used in other figures and represent marginally expressed transcripts (RPKM<0.5).

Figure S34

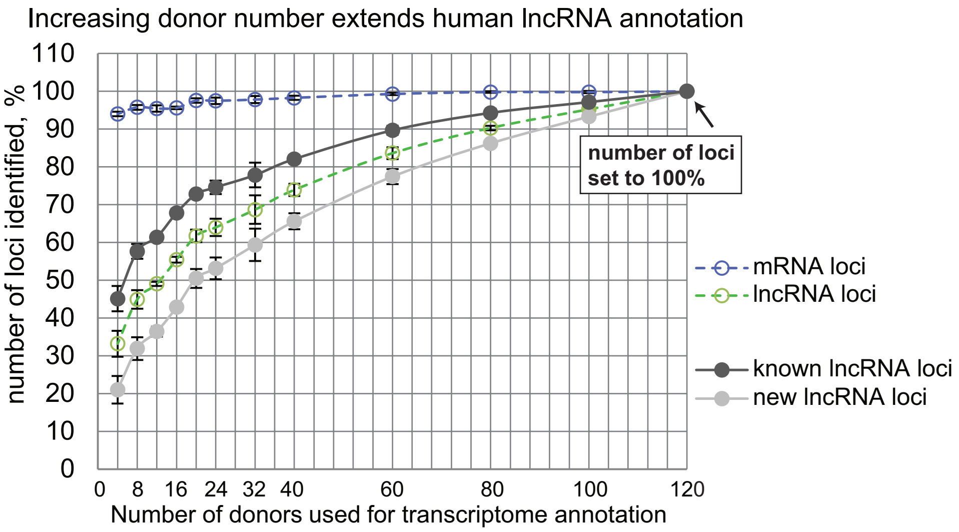

Figure S34. Number of new lncRNA increases more dramatically with donor number increase compared to known lncRNAs.

The dynamics of *de novo* identification of new (light gray) and known (dark gray) lncRNA loci in LCL using an increasing donor number (new – not covered by reference public annotations, known – covered by reference public annotations. As described for Fig. 2A). Dynamics for all lncRNA loci (dashed green line) and mRNA loci (dashed blue line) is indicated for comparison. The loci number is normalized to the total number of loci in the most comprehensive 120-donor annotation and set to 100% for each curve. Maximum number (100%) for new lncRNA loci: 2,063, maximum number (100%) for known lncRNA loci: 2,103. Error bars indicate standard deviation between 3 replicates of random picking for each number of assemblies used (Additional File 11C).

Figure S35

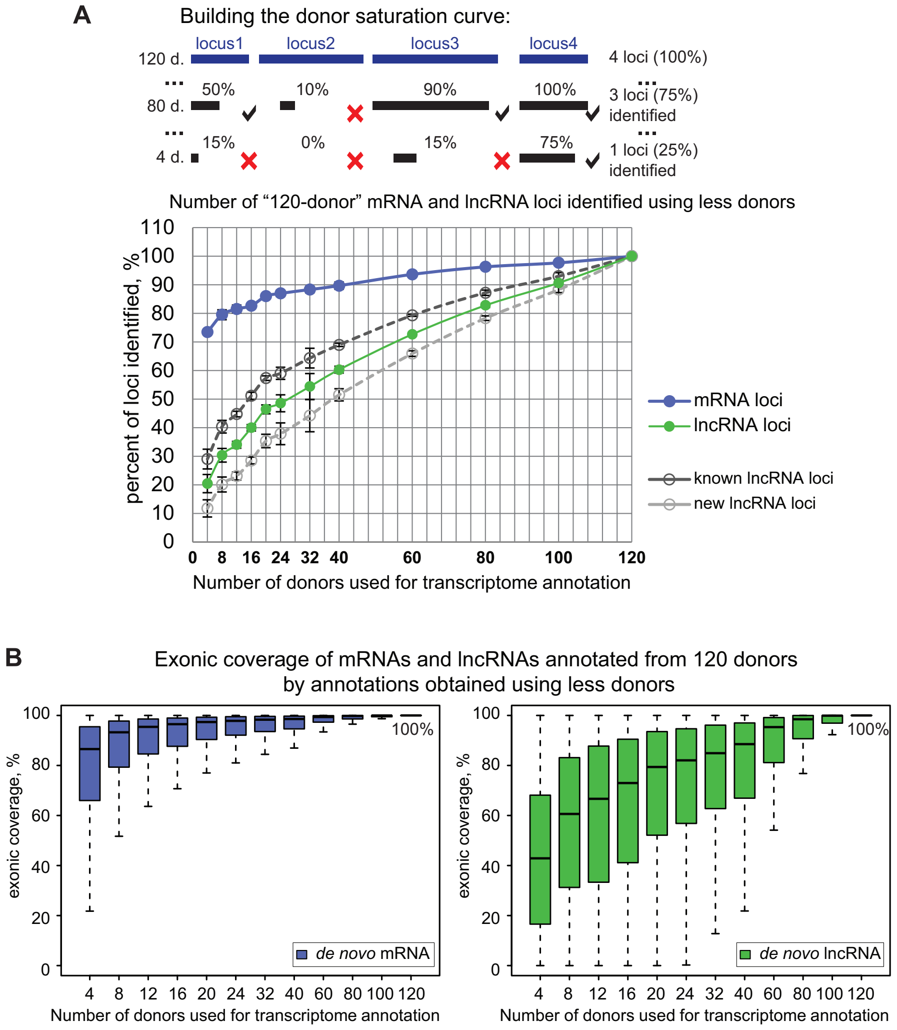

Figure S35. Donor saturation curve analysis of 120-donor lncRNA and mRNA identification using less donor in the identification pipeline

A. Donor saturation analysis. Top: definition of an "identified locus": lncRNA and mRNA annotation obtained using 120 donors (the most comprehensive annotation) is used as a reference for comparison and the number of loci in this annotation is set to 100%. When analyzing annotations obtained from fewer donors, a reference locus is called "identified" if it is covered by the down-sampled annotation to at least 50% of its length (black tick). In case the coverage is less the locus is not considered "identified" (red cross). Below: normalized number of 120-donor reference annotation loci "identified" when running the identification pipeline with fewer donors. Displayed are donor saturation curves for mRNA loci (blue), all lncRNA loci (green), only new lncRNA loci (unfilled light grey circles and dashed line) and only known lncRNA loci (unfilled dark grey circles and dashed line). Number loci in the 120-donor annotation was set to 100% for all the four displayed loci type. Error bars: representing standard deviation between 3 replicates are present for all data points but due to low values are mostly not visible (Additional File 11D).

B. Donor saturation curve of exon structure identification. The plot shows the percent exonic coverage (Supplemental Methods) matching the reference annotation generated from 120 donors (set to 100%), obtained using fewer donors. Left boxplot: *de novo* mRNA transcripts (blue), right boxplot: *de novo* lncRNA transcripts (green). Outliers are not displayed. Median exonic coverage values (from left to right): mRNAs – 86.6%, 93.3%, 95.4%, 96.5%, 97.4%, 97.8%, 98.3%, 98.6%, 99.4%, 99.8%, 100.0%, **100%**, lncRNAs – 42.9%, 60.6%, 66.7%, 73.0%, 79.4%, 82.0%, 84.9%, 88.5%, 95.3%, 98.5%, 99.9%, **100%**

SUPPLEMENTAL METHODS

1. Granulocyte isolation
2. RNA isolation using TRI reagent
3. Reverse transcription
4. Ribosomal RNA depletion
5. Polyadenylated RNA enrichment
6. Preparation of strand-specific RNA-seq libraries
7. Public gene annotations used in the study
8. RNA-seq read alignment
9. Calculation of GC content
10. RT and qRT-PCR primer design
11. Annotating mRNAs and lncRNAs in primary granulocytes *de novo*
    11.1 Filtering steps
        11.1.1 Filtering for mRNAs
        11.1.2 Filtering for lncRNAs
    11.2 Combining *de novo* lncRNA and mRNA transcripts into genomic loci
    11.3 Protein-coding potential calculation pipeline
12. Calculating exonic coverage
13. Creating granulocyte specificity estimation heat maps
14. RT-PCRs to control splicing efficiency calculation

1. Granulocyte isolation

Granulocytes and mononuclear cells (MNCs) were isolated from freshly collected blood using gradient density centrifugation (Ficoll-Plaque PREMIUM 1.078 g/ml, GE Healthcare Life Sciences). Briefly, 45 ml of fresh blood was centrifuged at 100g for 10 minutes at room temperature and the yellowish supernatant was discarded to remove the platelet rich plasma. The remainder was diluted approximately four fold with room temperature (RT) PBS (+2 mM EDTA) to 144 ml. 35 ml of diluted blood was carefully layered on top of 15 ml of Ficoll (equilibrated at RT) in a 50 ml Falcon tube and four such tubes were centrifuged at RT at 400g for 33 minutes with acceleration/brake at minimum. After centrifugation the upper layer was carefully removed and discarded, the MNC layer immediately on top of the Ficoll separation layer was collected into a new tube and washed in ice cold PBS (+2mM EDTA) by centrifugation at 300g for 10 minutes. The upper Ficoll layer was carefully removed and the underlying remaining layer containing the granulocyte population was depleted for erythrocytes using Cell Lysis Solution (Promega) in two 5 minute incubation steps followed by 5 minute 300g centrifugation at RT. Granulocytes were then washed in ice cold PBS (+2 mM EDTA) by centrifugation at 300g for 5 minutes. Both MNCs and granulocytes underwent one further ice-cold PBS (+2 mM EDTA) washing step (8 minutes at 200g) to remove residual platelets and to create a pellet for immediate RNA isolation

2. RNA isolation using TRI reagent

Pelleted cells were lysed in 1 ml of TRI reagent (Sigma-Aldrich T9424) per 10^7 cells by active pipetting / vortexing and incubated for 5 minutes at room temperature (RT) and the lysate frozen and stored at -80ºC for later RNA isolation. After thawing on ice, 0.1 ml BCP (Molecular Research Center, Inc.) was added per 1 ml of TRI reagent, followed by intensive shaking and 10 minute incubation at RT, and then centrifuged for 12 minutes at 12000g at 4ºC. The upper aqueous phase was transferred to a new tube with 0.5 ml isopropanol, vortexed and incubated for 10 minutes at RT to allow RNA precipitation. The RNA precipitate was pelleted by centrifugation at 12000g for 12 minutes at 4ºC, the supernatant was removed and the pellet washed with 1 ml of 70% ethanol (7500g, 5 minutes, 4ºC). After ethanol removal, the pellet was air-dried for few minutes and dissolved in RNA Storage Solution (RSS) (Ambion) and stored at 80ºC. DNaseI treatment was performed for 10 μg RNA per 50μl reaction, using the DNA-free kit (Ambion).

3. Reverse transcription

DNase I treated RNA was reversely transcribed into cDNA using RevertAid First Strand cDNA Kit (Fermentas). 0.6 μg of RNA per 20 μl was used and –RT control (lacking Reverse Transcriptase enzyme) was performed for each set of RT reactions.

4. Ribosomal RNA depletion

Total RNA was depleted for ribosomal RNA using the RiboZero rRNA removal kit Human/Mouse/Rat (Epicentre). 2-4 μg of DNase I treated RNA was used for each reaction. At the end of the protocol the volume of RiboZero treated RNA was adjusted to 180 μl and RNA was precipitated by adding 18 μl of 3M sodium acetate (Ambion), 2 μl of Glycogen (10mg/ml) and 600 μl of ice-cold 96% ethanol. Following overnight incubation at -20ºC the RNA precipitate was recovered by centrifugation (50 minutes, 16000g, 4ºC). The RNA pellet was washed as described above, dissolved in 1 μl nuclease-free water and diluted with 19.5 μl of Elute, Prime, Fragment Mix (TruSeq RNA Sample Prep Kit v2, Illumina). After incubation at 94ºC for 3 minutes to allow priming and fragmentation of RNA, 17 μl was transferred to DNA LoBind Tubes (Eppendorf) for immediate stranded library preparation.

5. Polyadenylated RNA enrichment

PolyA enriched RNA was prepared using the TruSeq RNA Sample Prep Kit v2 (Illumina) and 2-4 μg of DNase I treated RNA. At the end of the protocol the magnetic bead-bound RNA (Agencourt AMPure XP - PCR Purification, Beckman Coulter) was incubated with 19.5 μl of Elute, Prime, Fragment Mix (TruSeq RNA Sample Prep Kit v2, Illumina) at 94ºC for 3 minutes and the magnetic beads were pelleted using a magnetic stand. 17 μl of supernatant was transferred to a DNA LoBind Tube (Eppendorf) for immediate stranded or non-stranded library preparation.

6. Preparation of strand-specific RNA-seq libraries

First-strand cDNA synthesis: 8 μl of First Strand Master Mix (TruSeq RNA Sample Prep Kit v2, Illumina), supplemented with 1 μl SuperScript II per 9 μl First Strand Master Mix, was added to 17 μl of either PolyA+ or total RNA, followed by vortexing and incubation consequently at 25ºC for 10 minutes, 42ºC for 50 minutes, 70ºC for 15 minutes to perform reverse transcription reaction. First-strand cDNA was then cleaned using G-50 columns (Illustra ProbeQuant G-50 Micro Columns, GE

Healthcare). G-50 columns were preliminarily washed twice with 500 μl of 1 mM Tris-HCl pH 8.0 by centrifugation at 700g for 2 minutes at RT. First-strand cDNA was diluted to 30 μl by adding 5 μl of Elution Buffer (TruSeq RNA Sample Prep Kit v2, Illumina) and then added to the G-50 column and centrifuged at 700g for 2 min at RT. The eluate was then adjusted to 52 μl by adding nuclease-free water.

Second-strand cDNA synthesis: Second Strand master mix was prepared freshly as follows: 1 μl of 10 x Reverse Transcription Buffer (Invitrogen), 15 μl of 5 x Second Strand Syntheses Buffer (Invitrogen), 1 μl 50 mM $MgCl_2$, 1 μl of 100 mM DL-Dithiothreitol (DTT, Invitrogen), 2 μl of dUNTP mix (10 mM dATP, 10 mM dCTP, 10 mM dGTP, 10 mM dUTP) (Thermo Scientific), 0.5 μl of 10 U/μl E.coli DNA ligase (Invitrogen), 2 μl of 10 U/μl DNA Polymerase (Invitrogen) and 0.5 μl 2U/μl RNase H (Invitrogen). 23 μl of the master mix was added to the cleaned first-strand cDNA sample and incubated at 16ºC for 2 hours. The cDNA was then cleaned using magnetic beads (Agencourt AMPure XP - PCR Purification, Beckman Coulter). 135 μl of RT pre-vortexed beads was added to the reaction, the mixture was incubated for 15 minutes at RT to allow binding and put on the magnetic stand for 5 minutes to collect the beads. Supernatant was discarded and the bead pellet was washed twice with 200 μl freshly prepared 80% ethanol and air-dried for 15 minutes. The beads were then resuspended in 52.5 μl Resuspension Buffer (TruSeq RNA Sample Prep Kit v2, Illumina), incubated for 2 minutes at RT and put on magnetic stand for 2 minutes to collect the beads. 50 μl of the supernatant containing cleaned double stranded cDNA were transferred to a new DNA LoBind Tube.

End repair procedure: 40 μl of End Repair Mix (TruSeq RNA Sample Prep Kit v2, Illumina) was added to 50 μl of double stranded cDNA. The mixture was vortexed and incubated at 30ºC for 30 minutes. Afterwards the cDNA was cleaned by the procedure described above using 160 μl of magnetic beads. cDNA was eluted from beads using 20 μl of Resuspension Buffer.

3'end adenylation: After the end repair 3' ends of cDNA were adenylated by adding 12.5 μl of A-Tailing Mix (TruSeq RNA Sample Prep Kit v2, Illumina) and incubating the mix at 37ºC for 30 minutes and then at 70ºC for 5 minutes.

Adapter ligation: The adapters (barcodes) provided by TruSeq RNA Sample Prep Kit v2 were chosen according to the plan of how to pool the libraries to be sequenced on one lane. To ligate a desired adapter to cDNA 2.5 μl of Resuspension Buffer, 2.5 μl of Ligation Mix (TruSeq RNA Sample Prep Kit v2, Illumina) and 2.5 μl of the RNA Adapter Index (TruSeq RNA Sample Prep Kit v2, Illumina) were added to the reaction and incubated at 30ºC for 10 minutes. To stop the ligation we added 5 μl of Stop Ligation Buffer (TruSeq RNA Sample Prep Kit v2, Illumina) to the reaction. Afterwards the cDNA was cleaned and eluted twice by the procedure described above: first time with 42 μl of magnetic beads and 52.5 μl of Resuspension Buffer and second time with 50 μl of magnetic beads and 22.5 μl of Resuspension Buffer.

UDGase treatment: Removal of the second strand cDNA with incorporated dUTPs was performed by adding 2.3 μl of 10 x UNG Buffer (company) and 1 μl of 5 U/μl UDGase (company) to cleaned adapter-ligated cDNA and incubation at 37ºC for 30 minutes.

Library enrichment by PCR**:** To enrich for cDNA fragments PCR was performed by adding 5 μl of PCR Primer Cocktail (TruSeq RNA Sample Prep Kit v2, Illumina), 25 μl of PCR Master Mix, vortexing and running the PCR program: 98ºC 30 seconds, 8 cycles (98ºC 10 seconds, 60ºC 30 seconds, 72ºC 30 seconds), 72ºC 5 minutes). The PCR reaction was then cleaned and eluted twice by the procedure described above: first time with 42 μl of magnetic beads and 52.5 μl of Resuspension Buffer and second time with 53 μl of magnetic beads and 20 μl of Resuspension Buffer.

7. RNA-seq read alignment

Raw RNA-seq reads were aligned with STAR [6]. The default STAR options were optimized in order to give a stringent unbiased alignment. (i) the hg19 genome was built without pre-annotated splice junctions (SJ) to allow non-biased *de novo* splice junction identification: *STAR_2.3 --runMode genomeGenerate --genomeSAindexNbases 13 --genomeChrBinNbits 18 --genomeSAsparseD 2 --genomeDir [hg19genome_for_STAR] --genomeFastaFiles hg19.fa --runThreadN 8.* (ii) to be stringent in novel splice site discovery we only considered canonical SJs using *--outFilterIntronMotifs RemoveNoncanonical* (keeping only canonical SJ was also important for further Cufflinks transcriptome assembly from non-strand-specific data), required the minimum overhang length for SJs on both sides to be >= 16bp by *--outSJfilterOverhangMin 30 16 16 16* , required that a SJ is supported by at least 2 reads by *--outSJfilterCountTotalMin 4 2 2 2*, required the minimum overhang of a spliced read to be >=6 by *--alignSJoverhangMin 6*, required to only keep the spliced reads which passed the previous filtering requirements by *--outFilterType BySJout*. (iii) we required that intron size <=300kb (*--alignIntronMax 300000*) and the maximum gat between two read mate <=500kb (*--alignMatesGapMax 500000*). The alignment was performed using with the following command (all the not shown options were set to default), followed by sorting and indexing of the resulting BAM file: *STAR_2.3 --genomeDir [hg19genome_for_STAR] --readFilesIn READ1.fastq.gz READ2.fastq.gz --readFilesCommand zcat --outSJfilterOverhangMin 30 16 16 16 --outSJfilterCountTotalMin 4 2 2 2 --alignSJoverhangMin 6 --outFilterType BySJout --outSAMstrandField intronMotif --outFilterIntronMotifs RemoveNoncanonical --alignIntronMax 300000 --alignMatesGapMax 500000 --runThreadN 8 --outFileNamePrefix outprefix --outStd SAM --outSAMmode Full | samtools view -bS - > outprefix.bam*

#sort the bam file#: samtools sort outprefix.bam outprefix.sorted

#create indexed bam file#: samtools index outprefix.sorted.bam

Strand specificity of the aligned data was assessed using RSEQC package:

infer_experiment.py –r RefSeq_mRNAs.bed –i alignment.bam

Insert size of the libraries was assessed using RSEQC package:

inner_distance.py –r RefSeq_mRNAs.bed –i alignment.bam

8. Public gene annotations used in the study

We used a public annotation of lncRNAs provided by the GENCODE project (http://www.gencodegenes.org/releases/19.html). GENCODE v19 lncRNA annotation consists of 23,898 transcripts (21,523 multi-exonic transcripts). We also used lncRNA annotation provided by RefSeq (NR_* (>200nt cDNA length) annotation downloaded from the UCSC table browser on 3 June 2014) and lncRNA annotation published by Cabili et al [3]. RefSeq lncRNA annotation consists of 8,236 transcripts (7,603 multi-exonic transcripts). Cabili et al lncRNA annotation consists of 21,630 transcripts (21,595 multi-exonic transcripts). We used two public annotations of protein-coding genes: RefSeq (NM_* (mRNA) annotation downloaded from the UCSC table browser on 27 January 2014) and an annotation provided by the GENCODE project. RefSeq mRNA annotation consists of 39,562 transcripts (37,933 multi-exonic transcripts). GENCODE v19 mRNA annotation consists of 81,814 transcripts (80,035 multi-exonic transcripts). We used a public annotation of pseudogenes provided by the GENCODE project (GENCODE v19 – 17,572 pseudogenes). We used an annotation of repeat elements – RepeatMasker downloaded from the UCSC browser.

9. Calculation of GC content

GC content of selected regions was calculated using bedtools *nuc -fi hg19.fa -bed regions.bed*

10. RT- and qRT-PCR primer design

Primers for RT- and qRT-PCR were designed using Primer3 software (http://biotools.umassmed.edu/bioapps/primer3_www.cgi).

11. Annotating mRNAs and lncRNAs in primary granulocytes

Transcriptome assembly: 17 PolyA+ RNA-seq data sets (comprising ten different healthy donors) with total of 784 M mapped reads were used to create the granulocyte mRNA and lncRNA annotations used for further analysis (Additional File 2A, B). Although it is suggested that each sample's transcriptome should be assembled separately [7], PolyA+ datasets were pooled into 6 parts at the stage of alignment in order to increase the sensitivity of the transcriptome assembly (Additional File 2C). Pools were created so that they contained 24-37 M spliced reads each (85-102 M mapped reads for 4 x 100bp paired-end pools and ~ 220 M reads for 2 x 50 bp paired-end pools). Six alignment .BAM files were created as described in Methods and then used to perform six separate transcriptome assemblies using Cufflinks [7]. In order to prevent a bias towards identification of already annotated transcripts no reference gene annotation was provided to Cufflinks. In order to avoid Cufflinks pausing over problematic regions in the genome annotated pseudogenes (GENCODE v 19) were masked using -- *mask-file* option. Cufflinks was run for 6 .BAM files (6 pools' alignments) with the following options: *cufflinks --multi-read-correct --output-dir [output_dir] -F 0.01 -p 7 --library-type fr-unstranded (for stranded pools --library-type fr-firststrand) --mask-file pseudogenes.gtf PolyA_pool_N.sorted.bam*
Removal of mono-exonic transcripts: All single exon transcripts were removed from each of the 6 transcriptome assemblies using *gffread* tool (part of Cufflinks package): *gffread transcripts.gtf -T -U -o transcripts_multiexon.gtf*. The three main rationales for focusing on multi-exonic transcripts were the following. First, the majority of mono-exonic transcripts assembled by Cufflinks appear to be intronic signals and other artifacts. Second, mono-exonic transcripts assembled by Cufflinks are not continuous unlike spliced transcripts whose continuity is supported by the spliced reads spanning thousands of kilobases. Third, 13 out of 17 PolyA+ datasets we used for the assembly were not strand-specific. However, the presence of a splice site in a transcript allowed Cufflinks to infer the strand it was transcribed from. Previous publications extensively used non-strand-specific data for Cufflinks based annotation of multi-exonic lncRNAs in human and have shown that the error rate of inferring the strand from the canonical splice sites was negligible [3, 8]. Additionally, to be maximally stringent, we also removed potential strand specificity artifacts at later filtering steps.
Merging the annotations: The resulting six multi-exonic transcriptome annotations were merged using Cuffmerge with the following command: *cuffmerge -s hg19.fa --keep-tmp -p 8 --min-isoform-fraction 0 list_of_6_annotation_files.txt*. The resulting merged annotation contained 158,038 transcripts comprising 13,589 loci.

11.1. Filtering Steps

The merged transcriptome annotation was then filtered in order to create granulocyte mRNA and lncRNA annotations for further use in the study. It was necessary to *de novo* annotated mRNA genes as we noticed that comparison of *de novo* annotated lncRNAs to mRNAs annotated by RefSeq or GENCODE, was misleading due to the precision of the *de novo* annotation for granulocytes where only the isoforms actually expressed in granulocytes were annotated, and due to potential artifacts of *de novo* annotation missing from the curated, comprehensive and experimentally supported public mRNA annotations. Thus, in order to avoid potential technical biases, mRNA annotation was created *de novo* in granulocyte using the same pipeline that was used for lncRNAs. The following common filtering steps for both mRNAs and lncRNAs:

Expression cut off: We used 6 pools of RNA-seq data to increase the sensitivity of Cufflinks to lowly expressed spliced isoforms of lncRNAs. However, the increased sensitivity could potentially result in false positive transcripts. We checked if the assembled transcripts could be detected in at least one of the diverse granulocytes RNA-seq samples from 10 individuals used in the study. We used an expression level calculation method independent from Cufflinks (*RPKM_count.py* from RSeQC package) to analyze the expression of all the *de novo* assembled transcripts in all the available granulocyte RNA-seq datasets (17 PolyA+ RNA-seq datasets + 21 total RNA-seq datasets). If a transcript was not expressed (RPKM<=0.2) in any of the datasets, we called it an artifact and removed from the annotation. By this step 0.4% (631) of transcripts was removed resulting in residual 157,407 transcripts.

Filtering out transcripts potentially assigned to the wrong strand: Although Cufflinks infers the strand of the transcript from the direction of spliced junctions within this transcript, we wanted to be stringent and remove potential artifacts assigned to the wrong strand. For that we performed two steps of filtering. First, using a custom script, we checked if the *de novo* annotation contains transcripts that have a "mirror" transcript on the other strand (that is a transcript that has exons with > 30% reciprocal antisense overlap with exons of a transcript on another strand). Such transcripts arising from problems in the strand-specificity step could be potential artifacts and had to be removed in order to create a stringent set of transcripts. In each pair of such transcripts we then kept the one with the higher expression level. As an expression level estimate for each transcript we took the maximum RPKM among all the stranded RNA-seq datasets (four PolyA+ RNA-seq datasets and 21 total RNA-seq datasets). By this step 1.4% (2,273) of transcripts was removed from the annotation, resulting in residual 155,134 transcripts. 107 transcripts that fulfilled the criteria were not expressed in any of the stranded samples (RPKM<=0.2), and therefore could not be filtered out and were kept in the annotation and run through the next stage of filtering. Second, transcripts that had exons with >20% reciprocal antisense overlap with exons of an annotated mRNA (RefSeq or GENCODE v19) or lncRNA (GENCODE v19) expressed in any of the 6 pools were removed from the annotation. By this step 2.0% (3,142) of transcripts was removed from the annotation, resulting in residual 151,992 transcripts.

Size cut off: LncRNA transcripts are by definition longer than 200nt. Therefore we removed all the transcripts whose summary exon length was <200nt. By this step 0.02% (37) of transcripts was removed from the annotation, resulting in residual 151,955 transcripts.

Exon length cut off: To further remove potential artifacts from the annotation, we filtered out the transcripts with unusually long exons and with an unusually high exon/intron length ratio. To set the cutoff we checked the properties of the annotated mRNA and lncRNA genes and found that 99.4 % of GENCODE multi-exonic lncRNAs, 99.7% of GENCODE multi-exonic mRNAs and 99.7% RefSeq multi-exonic mRNAs do not have exons longer than 15kb and their exons constitute less than 90% of total gene length (Figure S1C). We removed all the transcripts not fulfilling these criteria. By this step

3.9% (5,939) of transcripts was removed from the annotation, resulting in residual 146,016 transcripts.
Repeat coverage cut off: lncRNAs are rich in repeat elements [9] and we allowed reads that mapped in several locations in the genome to be aligned (see *RNA sequencing read alignment* above). Therefore, we allowed some repeat elements to be mapped and potentially assembled as transcripts. It was necessary then to remove the transcripts assembled mainly from repeat regions and thus potentially being artifacts. Using the following command containing the custom script - *bed12ToBed6 -i annotation.bed | coverageBed -b stdin -a RepeatMaskUCSC.bed | perl repeat_coveage.pl > coverage_of_genes* - we performed a control check of annotated multi-exonic lncRNA and mRNA genes (lncRNAs by GENCODE v19, mRNAs by GENCODE v19 and RefSeq – see above Public gene annotations used in the study) and found that repeat coverage of exons of 95,5% of GENCODE multi-exonic lncRNAs, 99,87% of GENCODE multi-exonic mRNAs and 99,96% RefSeq multi-exonic mRNAs did not exceed 80% (Figure S1D). By that we set the cutoff to 80% for the filtering and, using the command given above, removed all the *de novo* transcripts whose exons were covered by repeats more than 80%. By this step 0.7% (1,029) of transcripts was removed from the annotation, resulting in residual 144,987 transcripts.

11.1.1. Filtering for mRNAs - Creating *de novo* mRNA annotation in primary granulocytes

Overlap with annotated protein-coding genes: As the protein-coding genome is very well annotated we defined *de novo* mRNAs as transcripts that overlapped exons of protein-coding genes annotated by RefSeq or GENCODE v19 in the sense orientation (we used intersectBed tool with the –split option). This filtering step resulted in 136,482 transcripts being called protein-coding based on their overlap with the annotation.
Filtering out transcripts spanning from mRNAs to annotated lncRNAs: Some *de novo* transcripts spanned over more than one gene, which can be caused by Cufflinks artificially joining spliced transcripts located close to each other. However it is also known that transcription from a gene can run through a downstream gene and use its splice sites to create a chimera transcript [10]. We aimed to remove such chimera transcripts. While it was possible to remove protein-coding transcripts that span more than one protein-coding gene, due to the poor annotation of lncRNAs, we could not exclude transcripts that comprise a chimera of two lncRNA genes merged together by Cufflinks. As our goal was to process *de novo* mRNA transcripts similarly to *de novo* lncRNA transcripts, we did not apply this filtering to *de novo* mRNAs or lncRNAs. However, we could exclude the case when an artifact chimeric transcript combines an mRNA with a lncRNA. D*e novo* lncRNAs were filtered not to share a sense exonic overlap with annotated protein-coding genes (see below) and we similarly removed *de novo* mRNA transcripts that spanned to a GENCODE v19 annotated lncRNA (note, that some annotated lncRNAs do have a sense exonic overlap with annotated mRNAs and we took care of such cases). By this step 3.7% (5,059) of *de novo* mRNA transcripts were removed from the annotation, resulting in residual 131,423 transcripts.

11.1.2. Filtering for lncRNAs - Creating *de novo* lncRNA annotation in granulocytes

Filtering out transcripts: protein-coding genes and pseudogenes: To form a preliminary *de novo* lncRNA annotation the transcripts that passed all the common filtering steps, but had any exonic sense overlap with a protein-coding gene (GENCODE or RefSeq) or a pseudogene (GENCODE) were removed. By this filtering step 94.6% (139,080) of all lncRNA transcripts were removed from the annotation, resulting in residual 7,862 transcripts.

Filtering out extensions of protein-coding genes: While creating the *de novo* mRNA set we also identified 3' or 5' extensions of annotated protein-coding genes. The previous lncRNA filtering step, which excluded all transcripts overlapping the exons of annotated protein-coding genes could leave in transcripts corresponding to these extensions. To exclude this possibility, we removed transcripts that had any exonic sense overlap with the *de novo* annotated mRNAs. By this filtering step 6.0% (476) of lncRNA transcripts were removed from the annotation, resulting in residual 7,386 lncRNA transcripts.

Removing transcripts that overlap protein-coding genes in the sense direction: Out of 7,386 lncRNA transcripts 317 overlapped annotated or *de novo* assembled mRNA genes in the sense direction. We removed these transcripts from the list of *de novo* lncRNAs to avoid confusing their expression with the expression of overlapped protein-coding gene during the expression variation analysis. After removing sense overlapping transcripts we obtained a list of 7,069 *de novo* lncRNA transcripts.

11.2. Combining de novo lncRNA and mRNA transcripts into genomic loci

Transcripts were initially grouped into loci by Cuffmerge. However, after the filtering and artifact removal, many transcripts were removed and the rest had to be slightly regrouped. For example, if a *de novo* transcript spanned both a protein-coding gene and a lncRNA gene, two separate loci would be first grouped into one by Cuffmerge. After the removal of the artifact spanning transcript, the transcripts corresponding to a protein-coding gene would form one locus, and transcripts corresponding to a lncRNA gene would form another. We redefined the locus definition to account for removal of some transcripts using a custom script. 131,423 *de novo* mRNA transcripts formed 10,029 genomic loci with a mean of 13.1 transcripts per locus (median – 10 transcripts per locus). 7,069 *de novo* lncRNA transcripts formed 1,691 genomic loci with a mean of 3.9 transcripts per locus (median – 1 transcript per locus).

11.3. Protein-coding potential calculation pipeline

We based our mRNA *de novo* annotation on filtering for transcripts exonically overlapping annotated protein-coding genes. On the other hand, we based the *de novo* annotation of lncRNAs filtering for transcripts that had no exonic overlap with annotated protein-coding genes. However, although we combined both RefSeq and GENCODE v19 public annotations for protein-coding genes, a possibility remained that within the lncRNA list there were unknown transcripts coding for proteins. To test the coding potential of transcripts remaining in the lncRNA list, we performed an estimation of protein-coding potential of each *de novo* annotated transcript. We used a combination of two previously developed tools: RNAcode [11] and Coding Potential Calculator or CPC [12]. We used a local version of CPC (cpc-0.0-r2) that was modified to work with HMMER 3.0 [13] instead of blastx using UniProtKB/Swiss-Prot database (Jan 2012) (ftp://ftp.uniprot.org/pub/databases/uniprot/current_release/knowledgebase/complete/uniprot_sprot.fasta). cDNA of the transcript for HMMER input was created using the getfasta tool from the bedtools suite: *bedtools getfasta -bed [bed12 file] -fi mm10.fa -s -split -fo [cDNA FASTA file]*. Potential peptides originating from this transcript were determined using the transeq tool from EMBOSS6.5.7: *transeq -frame F [cDNA FASTA file] [translated protein sequence]*. The result of this program is a continuously translated protein sequence for each of the three forward frames. As this sequence contains many stop codons we reduced the runtime of HMMER by extracting those peptide sequences that are between two stop codons using a custom script. The first peptide sequence (before the first

stop) as well as the last peptide sequence (after the last stop) was retained if it was longer than 20 amino acids. All other sequences were retained if they contained a start codon (M) that was located more than 30 amino acids from the next stop. HMMER was used with the following command: *phmmer -E 0.0000000001 --cpu 3 –tblout [output file with alignments]*.

The exact criteria for calling a gene non-protein-coding were determined by analyzing a set of well-studied lncRNAs (H19, XIST, JPX, MALAT, NEAT1, TUSC7, ANRIL, MIAT, HULC, HOTTIP, HOTAIR and HOTAIRM1 - see Additional File 2D). We controlled for false positive results by then applying the pipeline to public annotations of coding and non-coding multi-exonic transcripts. With the chosen criteria 98.9% of RefSeq and 96.1% of GENCODE multi-exonic protein-coding transcripts were identified as protein-coding. Accordingly, 94.4% of multi-exonic GENCODE lncRNA transcripts were identified as non-protein-coding. The final criteria for calling a transcript non-coding were the following: CPC score of a transcript had to be less than 1.6. RNAcode score had to be less than 18 (in case genome alignments for 3 species were available for the transcript and RNAcode score could be calculated). We discarded all the de novo lncRNA transcripts that were identified by the pipeline as having protein-coding potential. Moreover, if more than 15% of transcripts in one locus were called protein-coding, we discarded the whole locus. Thus, we obtained the final de novo lncRNA annotation consisting of 6,249 lncRNA transcripts. We lastly fine-tuned the loci definition (for loci where some isoforms were removed by the protein-coding potential filtering) and obtained the final annotation of 1,591 lncRNA loci.

12. Calculating exonic coverage

Exonic coverage of one ("reference") multiexonic annotation by another ("analyzed") was calculated using a custom Perl script. For each transcript of the reference annotation we looked for transcripts of the analyzed annotation which would exonically overlap it in the sense orientation. From these transcripts the one that covered the highest percentage of the exonic length of the reference transcript was picked and the exonic coverage given by this transcript was used as an output of the analysis for a given reference transcript.

13. Creating granulocyte specificity estimation heat maps

Each heat map was created from a table listing all the transcripts/loci for each annotation and corresponding RPKMs (calculated by RPKM_count.py) in 36 (GRA_pap, GRA_tot and 34 public RNA-seq samples) samples. Prior to building the heat map, an expression cut off was applied filtering for transcripts expressed (RPKM>0.2) in at least one sample, RPKMs were normalized to the maximum RPKM among all the samples for each transcript (row) and the maximum was set to one. The data table was then sorted using a custom script to organize the columns such that the transcripts showing >70% expression level relative to the maximum would be place on the top and then the rest of the table would be sorted the same way for the next column. This procedure was done consequently for all the columns. We then picked the transcripts/loci that fulfill "granulocyte specificity" criteria and placed them in the upper part of the table, and the rest of the transcripts/loci, to the lower part of the table. Then we used *pheatmap* function in R without the clustering option for rows or columns, to create the final heat maps.

14. RT-PCR to test splicing efficiency calculation

To test bioinformatic calculation of splicing efficiency we picked efficiently spliced (defined as mean splicing efficiency per transcript>80%, maximal splicing efficiency per transcript>90%) and inefficiently spliced (defined as mean splicing efficiency per transcript<50%, maximal splicing efficiency per transcript<70%) *de novo* granulocyte transcripts, eight each (4 x lncRNAs, 4 x mRNAs). We preliminary filtered lncRNA and mRNA transcripts to contain at least one junction which could be tested using our assay, i.e. which would be short enough to allow amplification of both spliced and unspliced products by standard PCR (junction length<1500bp). We additionally filtered the transcripts for relatively high expression (RPKM>1) to facilitate RT-PCR amplification. We also did not pick transcripts that were antisense to another gene since RT-PCR is not strand-specific. List of picked lncRNA and mRNA transcripts and primers to amplify the short junction are given in Figure S13B. Size of the expected PCR product amplifying the unspliced junction is given in the right-most column of the table in Figure S13B. RT-PCR program: 95° 3min, (95° 30sec, 59° 30sec, 72° 1 min) for 35 cycles, 72° 7min. Only the 8x2 randomly picked transcripts (one junction each) were tested. Only one primer pair per junction was tested.

SUPPLEMENTAL REFERENCES

1. Smit AFA: **RepeatMasker**. http://wwwrepeatmaskerorg 1996-2005.
2. Lappalainen T, Sammeth M, Friedlander MR, t Hoen PA, Monlong J, Rivas MA, Gonzalez-Porta M, Kurbatova N, Griebel T, Ferreira PG *et al*: **Transcriptome and genome sequencing uncovers functional variation in humans**. *Nature* 2013, **501**(7468):506-511.
3. Cabili MN, Trapnell C, Goff L, Koziol M, Tazon-Vega B, Regev A, Rinn JL: **Integrative annotation of human large intergenic noncoding RNAs reveals global properties and specific subclasses**. *Genes & development* 2011, **25**(18):1915-1927.
4. Harrow J, Frankish A, Gonzalez JM, Tapanari E, Diekhans M, Kokocinski F, Aken BL, Barrell D, Zadissa A, Searle S *et al*: **GENCODE: the reference human genome annotation for The ENCODE Project**. *Genome research* 2012, **22**(9):1760-1774.
5. Pruitt KD, Brown GR, Hiatt SM, Thibaud-Nissen F, Astashyn A, Ermolaeva O, Farrell CM, Hart J, Landrum MJ, McGarvey KM *et al*: **RefSeq: an update on mammalian reference sequences**. *Nucleic Acids Res* 2014, **42**(Database issue):D756-763.
6. Dobin A, Davis CA, Schlesinger F, Drenkow J, Zaleski C, Jha S, Batut P, Chaisson M, Gingeras TR: **STAR: ultrafast universal RNA-seq aligner**. *Bioinformatics* 2013, **29**(1):15-21.
7. Trapnell C, Roberts A, Goff L, Pertea G, Kim D, Kelley DR, Pimentel H, Salzberg SL, Rinn JL, Pachter L: **Differential gene and transcript expression analysis of RNA-seq experiments with TopHat and Cufflinks**. *Nat Protoc* 2012, **7**(3):562-578.
8. Necsulea A, Kaessmann H: **Evolutionary dynamics of coding and non-coding transcriptomes**. *Nat Rev Genet* 2014, **15**(11):734-748.
9. Johnson R, Guigo R: **The RIDL hypothesis: transposable elements as functional domains of long noncoding RNAs**. *Rna* 2014, **20**(7):959-976.
10. Gingeras TR: **Implications of chimaeric non-co-linear transcripts**. *Nature* 2009, **461**(7261):206-211.
11. Washietl S, Findeiss S, Muller SA, Kalkhof S, von Bergen M, Hofacker IL, Stadler PF, Goldman N: **RNAcode: robust discrimination of coding and noncoding regions in comparative sequence data**. *Rna* 2011, **17**(4):578-594.
12. Kong L, Zhang Y, Ye ZQ, Liu XQ, Zhao SQ, Wei L, Gao G: **CPC: assess the protein-coding potential of transcripts using sequence features and support vector machine**. *Nucleic Acids Res* 2007, **35**(Web Server issue):W345-349.
13. Mistry J, Finn RD, Eddy SR, Bateman A, Punta M: **Challenges in homology search: HMMER3 and convergent evolution of coiled-coil regions**. *Nucleic Acids Res* 2013, **41**(12):e121.
